# Furan Derivatives and Polyketides from the Fungus *Irpex lacteus*

**DOI:** 10.1007/s13659-020-00282-w

**Published:** 2020-11-12

**Authors:** Meng Wang, Zheng-Hui Li, Masahiko Isaka, Ji-Kai Liu, Tao Feng

**Affiliations:** 1 School of Pharmaceutical Sciences, South-Central University for Nationalities, Wuhan, 430074 People’s Republic of China; 2 National Center for Genetic Engineering and Biotechnology (BIOTEC), 113 Thailand Science Park, Pathumthani, 12120 Thailand

**Keywords:** *Irpex lacteus*, Furan derivatives, Polyketides

## Abstract

**Abstract:**

Eight new furan derivatives, irpexins A‒H (**1**‒**8**), two new polyketides, irpexins I and J (**9** and **10**), together with nine known compounds were isolated from the fermentation of *Irpex lacteus*. The structures and absolute configurations were elucidated on the basis of extensive spectroscopic methods and Mosher ester reaction. All compounds shows no cytotoxicity to human MCF-7 and Hela cancer cell lines at the concentration of 10 μM.

**Graphic Abstract:**

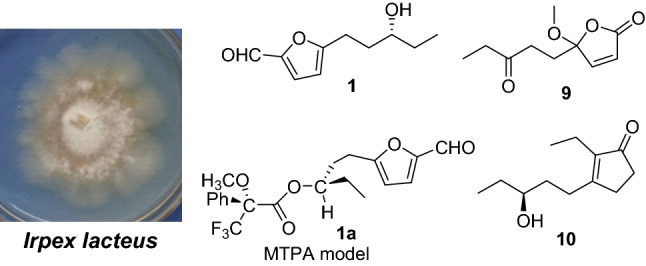

**Electronic supplementary material:**

The online version of this article (10.1007/s13659-020-00282-w) contains supplementary material, which is available to authorized users.

## Introduction

Fungi are important sources of natural products for the discovery of drugs and agricultural chemicals [1‒5], which have attracted great attention from pharmaceutical chemists and pharmacologists. *Irpex lacteus* is a pathogenic wood-decaying fungus belonging to the family Polyporaceae [6]. Its crude polysaccharide fraction has long been used as a traditional Chinese medicine for the treatment of chronic glomerulonephritis in clinic [7]. Chemists have researched the fermentation products of this fungus and the chemical constituents of fruiting bodies. In 1981, Hayashi et al. isolated three new nematicidal metabolites including two furan derivatives from their lactose liquid medium [8]. Our previous chemical investigations on the liquid fermentation of this fungus reported new carbon skeletons irlactins A–D that featured a 6/6 backbone [9], as well as a series of tremulane sesquiterpenoids [10, 11]. Later, studies on the fruiting bodies reported a series of triterpenes, irpeksins A–E, which inhibit the production of NO by RAW264.7 macrophages induced by LPS, and a new skeletal triterpenoid irpexolidal with a 6/5/6/5/6/5-fused polycyclic skeletal system [12, 13]. Recently, Duan et al. isolated four new bioactive metabolites from the endophytic fungus *Irpex lacteus* DR10-1, including one tremulane sesquiterpene irpexlacte A and three new furan derivatives irpexlactes B‒D [14]. In the current study, eight new furan derivatives, irpexins A‒H (**1**‒**8**), two new polyketides, irpexins I and J (**9** and **10**), together with nine known compounds were obtained from cultures of *I. lacteus* in rice medium. Their structures have been established by extensive spectroscopic methods, as well as modified Mosher’s method. All new compounds were evaluated for their cytotoxic activities against two human MCF-7 and Hela cell lines.

## Results and Discussion

**Fig. 1:**
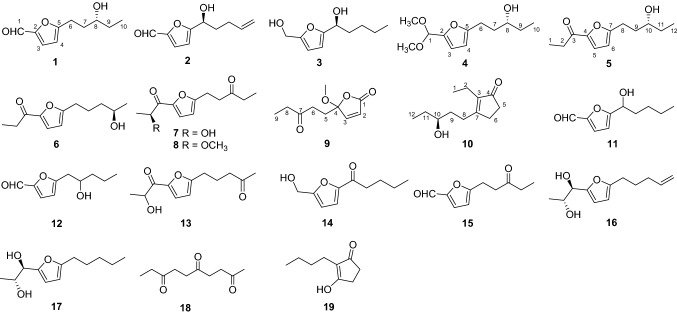
Chemical structures of compounds **1**‒**19**

**Fig. 2:**
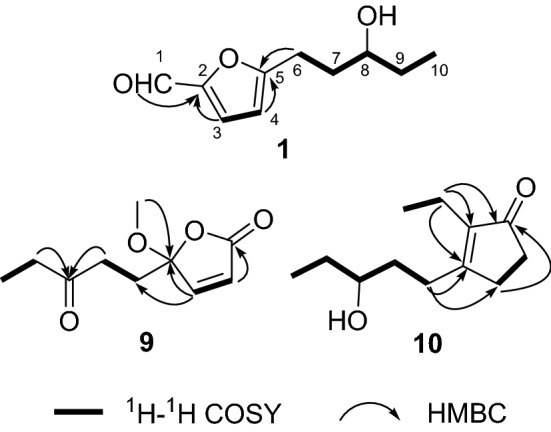
Four types of products of compound **1** reacting with *R-* and *S*-type Mosher reagents

Compound **1** was isolated as a colorless oil. Its molecular formula C_10_H_14_O_3_ was determined on the basis of positive high resolution (HR) ESI–MS at *m/z* 183.10158 [M + H]^+^ (calcd for C_10_H_15_O_3_^+^, 183.10157), corresponding to four degrees of unsaturation. In the ^1^H NMR spectrum (Table 1), one methyl group at *δ*_H_ 0.96 (3H, t, *J* = 7.5 Hz, H-10), one aldehyde proton at *δ*_H_ 9.50 (1H, s, H-1), and two olefinic protons at *δ*_H_ 7.19 (1H, d, *J* = 3.5 Hz, H-3) and 6.28 (1H, d, *J* = 3.5 Hz, H-4) were readily identified. The ^13^C NMR and DEPT data (Table 2) revealed ten carbon resonances ascribable for one CH_3_, three CH_2_, four CH, and two C. Of them, signals at *δ*_C_ 151.8 (s, C-2), 124.1 (d, C-3), 108.9 (d, C-4), 163.8 (s, C-5) were suggested to establish a furan moiety conjugated to a aldehyde group at *δ*_C_ 177.0 (d, C-1). These data were very similar to these of irpexlacte C [14]. The main difference was that the position of the hydroxyl group at C-7 in irpexlacte C was moved to C-8 in **1**, which was verified by the ^1^H-^1^H COSY correlation of *δ*_H_ 1.51 (2H, m, H-9) with *δ*_H_ 3.56 (2H, m, H-8) and of H-8 with H-10, together with the HMBC correlations from H-10 to *δ*_C_ 30.3 (t, C-9) and 72.2 (d, C-8). Therefore, a planar structure of **1** was figured out as shown in Fig. 1. The absolute configuration of **1** was determined by the Mosher reaction. Compound **1** reacted with *R*-Mosher reagent and *S*-Mosher reagent to obtain compounds **1a** and **1b** (for their ^1^H NMR spectra, see Figs. S8 and S9 in the Supporting Information), respectively. In the ^1^H NMR spectrum, the chemical shifts of *δ*_H_ 2.76–2.71 (2H, m, H-6) and *δ*_H_ 2.07–2.03 (2H, m, H-7) in **1a** were higher than *δ*_H_ 2.63–2.56 (2H, m, H-6) and *δ*_H_ 2.02–1.96 (2H, m, H-7) in **1b**, while the chemical shifts of *δ*_H_ 1.67–1.65 (2H, m, H-9) and *δ*_H_ 0.85–0.83 (3H, m, Me-10) were lower than *δ*_H_ 1.74–1.73 (2H, m, H-9) and *δ*_H_ 0.95–0.92 (3H, m, Me-10) in **1b**. Due to the shielding effect of phenyl, this result suggested that –C9‒C10– in **1a** was on the same side as phenyl in *R*-Mosher, and –C9‒C10– in **1b** is not on the same side as phenyl in *S*-Mosher corresponding to A-type and B-type in Fig. 2, respectively. At this point, the absolute configuration of C-8 was determined as *R*. Compound **1** was, therefore, established as irpexin A, as depicted.

**Table 1:** ^1^H NMR (600 MHz) Data of **1**‒**10** (*δ* in ppm and *J* in Hz)

No	**1**^a^	**2**^a^	**3**^a^	**4**^a^	**5**^a^	**6**^a^	**7**^a^	**8**^b^	**9**^a^	**10**^*a*^
1	9.50, s	9.59, s	4.59, s	5.38, s	1.20, t (7.4)	1.20, t (7.5)	1.47, d (7.0)	1.20, d (6.1)		0.98, t (7.6)
2					2.80, m	2.80, q (7.5)	4.83, q (7.0)	3.96, m	6.22, d (5.7)	2.21, q (7.6)
3	7.19, d (3.5)	7.22, d (3.5)	6.24, d (3.1)	6.31, d (3.1)				3.97, m	7.12, d (5.7)	
4	6.28, d (3.5)	6.49, d (3.5)	6.18, d (3.1)	5.98, d (3.1)						
5					7.11, d (3.5)	7.10, d (3.5)	7.23, d (3.5)	6.25, d (3.1)	2.69, m 2.55, m	2.37, m
6a 6b	2.93, m 2.83, m	4.82, dd (8.1, 5.1)	4.65, t (6.9)	2.80, m 2.71, m	6.18, d (3.5)	6.17, d (3.5)	6.25, d (3.5)	6.01, d (3.1)	2.27, m 2.11, m	2.51, m
7a 7b	1.91, m 1.77, m	1.98, m	1.86, m	1.84, m 1.71, m						
8a 8b	3.56, m	2.21, m	1.43, m 1.31, m	3.55, m	2.90, m 2.81, m	2.73, t (7.6)	3.03, t (7.3)	2.62, t (7.3)	2.44, m	2.60, m 2.52, m
9a 9b	1.51, m	6.84, m	1.36, m	1.53, m 1.47, m	1.89, m 1.77, m	1.83, m 1.74, m	2.84, t (7.3)	1.86, m	1.06, t (7.3)	1.72, m 1.63, m
10	0.96, t (7.5)	5.06, m	0.91, t (7.3)	0.95, t (7.5)	3.57, m	1.51, m		2.52, t (7.2)		3.56, m
11a 11b					1.53, m 1.49, m	3.84, m	2.46, q (7.3)			1.55, m 1.50, m
12					0.96, t (7.4)	1.21, d (6.2)	1.08, t (7.3)	2.10, s		0.97, t (7.6)
OCH_3_				3.35, s				3.23, s	3.20, s	
OCH_3_				3.35, s						

^a^Measured in CDCl_3_

^b^Measured in methanol-*d*_4_

**Table 2:** ^13^C NMR (150 MHz) Data of **1**‒**10** (δ in ppm and J in Hz)

No	**1**^a^	**2**^a^	**3**^a^	**4**^a^	**5**^a^	**6**^a^	**7**^a^	**8**^b^	**9**^a^	**10**^a^
1	177.0, d	177.5, d	57.5, t	98.0, d	8.4, q	8.4, q	22.3, q	17.9, q	169.7, s	13.3, q
2	151.8, s	152.0, s	153.3, s	149.0, s	31.3, t	31.3, t	69.3, d	81.4, d	124.7, d	16.3, t
3	124.1, d	123.1, d	108.4, d	109.1, d	189.7, s	189.7, s	190.0, s	68.2, d	153.6, d	142.0, s
4	108.9, d	108.6, d	106.5, d	105.4, d	151.2, s	151.2, s	148.6, s	150.5, s	110.4, s	210.0, s
5	163.8, s	163.4, s	157.0, s	156.1, s	118.6, d	118.5, d	120.9, d	109.8, d	35.7, t	34.3, t
6	24.7, t	67.4, d	67.8, d	24.3, t	108.1, d	108.0, d	109.1, d	105.5, d	30.9, t	29.0, t
7	34.5, t	34.6, t	55.1, t	35.0, t	161.4, s	161.3, s	161.2, s	155.3, s	210.0, s	173.2, s
8	72.2, d	29.4, t	27.7, t	72.5, d	24.7, t	28.2, t	22.4, t	26.7, t	36.0, t	27.3, t
9	30.3, t	137.3, d	22.4, t	30.2, t	34.7, t	23.9, t	39.5, t	21.8, t	7.8, q	34.5, t
10	9.9, q	115.7, t	14.0, q	9.9, q	72.3, d	38.5, t	209.0, s	41.8, t		72.9, d
11					30.3, t	67.7, d	35.9, t	210.0, s		30.3, t
12					9.9, q	23.6, q	7.7, q	28.5, q		9.8, q
OCH_3_				52.8, q				55.7, q	51.2, q	
OCH_3_				52.8, q						

^a^Measured in CDCl_3_

^b^Measured in methanol-*d*_4_

Compound **2** was isolated as a colorless oil. Its molecular formula C_10_H_12_O_3_ was determined on the basis of HR-ESI-MS, corresponding to five degrees of unsaturation. The ^13^C NMR and DEPT data of **2** were very similar to these of irpexlacte C [14], except for one double bond in **2**. In ^1^H-^1^H COSY spectrum, the correlation of *δ*_H_ 6.84 (1H, m, H-9) with *δ*_H_ 2.21 (1H, m, H-8) and *δ*_H_ 5.06 (2H, m, H-10) revealed a double bond was formed between C-9 and C-10 in **2**. The optical rotation of **2** ([*α*] = − 38.0), contrary to that of **1** ([*α*] =  + 44.8) and those analogues reported in the literature [15], indicated that the absolute configuration of the C-6 in the side chain was *S*. Compound **2** was, therefore, established as irpexin B, as depicted.

Compound **3** was isolated as a colorless oil. Its molecular formula C_10_H_16_O_3_ was determined on the basis of HR-ESI-MS, corresponding to three degrees of unsaturation. The ^13^C NMR and DEPT data (Table 2) of **3** were very similar to these of irpexlacte C [14], except that the aldehyde unit was replaced by a hydroxymethyl (*δ*_C_ 57.5) in **3**, as supported by the HMBC correlations from *δ*_H_ 4.59 (1H, s, H-1) to *δ*_C_ 153.3 (s, C-2) and 108.4 (d, C-3). The optical rotation of **3** ([*α*] =  − 16.1) suggested C-6 to be *S* form. Compound **3** was, therefore, established as irpexin C, as depicted.

Compound **4** was isolated as a colorless oil. Its molecular formula C_10_H_20_O_4_ was determined on the basis of HR-ESI–MS at *m/z* 251.12544 [M + Na]^+^ (calcd for C_12_H_20_O_4_Na^+^, 251.12538), corresponding to three degrees of unsaturation. The ^13^C NMR and DEPT data of **4** were very similar to these of **1** (Table 2), except that the aldehyde unit in **1** was replaced by a dimethoxymethine group as supported by the HMBC correlations from *δ*_H_ 5.38 (1H, s, H-1) to *δ*_C_ 149.0 (s, C-2), 109.1 (d, C-3) and two OCH_3_ (*δ*_C_ 52.8). The optical rotation of **4** ([*α*] =  + 37.5), related to that of **1**, revealed the absolute configuration of C-8 to be *R*. Compound **4** was, therefore, established as irpexin D, as depicted.

Compound **5** was isolated as a colorless oil. Its molecular formula C_12_H_18_O_3_ was determined on the basis of the HR-ESI–MS data at *m/z* 211.13280 [M + H]^+^ (calcd for C_12_H_19_O_3_^+^, 211.13287), corresponding to four degrees of unsaturation. The ^13^C NMR and DEPT data of **5** were very similar to these of **1** (Table 2), except for two additional carbon resonances linked to a carbonyl carbon forming a propionyl group, replacing the formyl group in **1**. It was supported by the ^1^H-^1^H COSY correlation of *δ*_H_ 1.20 (3H, t, *J* = 7.4 Hz, Me-1) with *δ*_H_ 2.80 (2H, m, H-2), together with the HMBC correlations from *δ*_H_ 7.11 (1H, d, H-5), H-1, and H-2 to *δ*_C_ 189.7 (s, C-3) and 151.2 (s, C-4). The optical rotation data ([*α*] =  + 14.6) indicated C-10 to be *R* form. Compound **5** was, therefore, established as irpexin E, as depicted.

Compound **6** was isolated as a colorless oil. Its molecular formula C_12_H_18_O_3_ was determined on the basis of HR-ESI-MS data. The ^1^H and ^13^C NMR spectra were very close to those of **5** (Tables 1 and 2), except for the hydroxyl group at C-10 (*δ*_C_ 72.3) in **5** moved to the C-11 (*δ*_C_ 67.7) in **6**, as verified by the ^1^H-^1^H COSY correlation of *δ*_H_ 3.84 (1H, m, H-11) with *δ*_H_ 1.51 (2H, m, H-10) and *δ*_H_ 1.21 (3H, d, *J* = 6.2 Hz, Me-12). The calculated optical rotation data ([*α*] =  − 10.7) matched well with the experimental data ([*α*] = − 14.3) for **6** suggested C-10 to be *R* form (see Supporting Information). Compound **6** was, therefore, established as irpexin I, as depicted.

Compound **7** was isolated as a colorless oil. Its molecular formula C_12_H_16_O_4_ was determined on the basis of the positive high resolution (HR) ESI-MS at m/z 225.11211 [M + H]^+^ (calcd for C_12_H_17_O_4_^+^, 225.11214), corresponding to five degrees of unsaturation. The ^13^C and DEPT spectra displayed 12 carbon resonances which were closely related to that of **5** (Table 2), except that the signals at *δ*_C_ 31.3 (t, C-2) and *δ*_C_ 72.3 (d, C-10) in **5** were replaced by signals of *δ*_C_ 69.3 (d, C-2) and *δ*_C_ 209.0 (s, C-10) in **7**, respectively. The ^1^H-^1^H COSY correlation of *δ*_H_ 1.47 (3H, d, *J* = 7.0 Hz, Me-1) with *δ*_H_ 4.83 (1H, q, *J* = 7.0 Hz, H-2) suggested that C-2 in **5** was substituted by a hydroxyl group. In addition, the ^1^H-^1^H COSY correlation of *δ*_H_ 3.03 (2H, t, *J* = 7.3 Hz, H-8) with *δ*_H_ 2.84 (2H, t, *J* = 7.3 Hz, H-9), and *δ*_H_ 2.46 (2H, q, *J* = 7.3 Hz, H-11) with *δ*_H_ 1.08 (3H, t, *J* = 7.3 Hz, Me-12), as well as the HMBC correlation from H-12 to C-10 and *δ*_C_ 35.9 (t, C-11) suggested that C-10 in **5** was substituted by a ketone group. The optical rotation ([*α*] =  − 14.0), comparing with those analogues reported in the literature [16], indicated C-2 to be *S* form. Therefore, the structure of **7** was established as irpexin G, as shown.

Compound **8** was isolated as a colorless oil. Its molecular formula C_13_H_18_O_4_ was determined on the basis of HR-ESI-MS. The ^13^C NMR and DEPT data of **8** were very similar to these of **7** (Table 2), except that the hydroxy at C-2 was replaced by a methoxy in **8**, as supported by a HMBC correlation from *δ*_H_ 3.23 (3H, s, OCH_3_) to *δ*_C_ 81.4 (d, C-2). Detailed analysis of 2D NMR data suggested that other parts of **8** were the same to those of **7**. Therefore, compound **8** was established as irpexin K, as depicted.

**Fig. 3:**
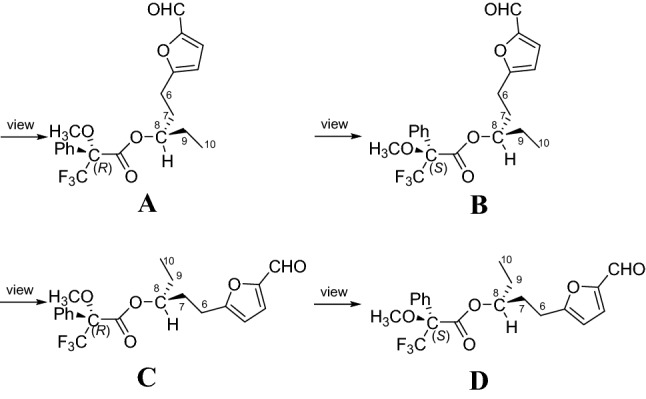
Key HMBC and ^1^H-^1^H COSY correlations of compounds **1**, **9**, and **10**

Compound **9** was isolated as a colorless oil. Its molecular formula C_10_H_14_O_4_ was determined on the basis of HR-ESI-MS at *m/z* 221.07843 [M + Na]^+^ (calcd for C_10_H_14_O_4_Na^+^, 221.07843), corresponding to four degrees of unsaturation. In the ^1^H NMR spectrum (Table 1), one methyl group at *δ*_H_ 1.06 (t, *J* = 7.3 Hz, Me-9), one methoxy group at *δ*_H_ 3.20 (s, OCH_3_), two olefinic protons at *δ*_H_ 6.22 (d, *J* = 5.7 Hz, H-2) and 7.12 (d, *J* = 5.7 Hz, H-3) were identified. The ^13^C and DEPT spectra (Table 2) displayed 10 carbon resonances corresponding to one CH_3_, one OCH_3_, three CH_2_, two CH (two olefinic carbon at *δ*_C_ 153.6 and 124.7), and three C (one ketone carbon at *δ*_C_ 210.0, one ester carbonyl at *δ*_C_ 169.7, one oxygenated sp^3^ quaternary carbon at *δ*_C_ 110.4) (Table 2). Considering one double bond, one ketone carbon and ester carbonyl, compound **9** should possess a single ring framework. Primary analysis of 2D NMR data indicated a five-membered unsaturated lactone as built by C-1 to C-4 [17], as well as a 3-pentone moiety link to C-4 (Fig. 3). In addition, one HMBC correlation from OMe to *δ*_C_ 110.4 (s, C-4) indicated one methoxy at C-4. The optical rotation ([*α*] = 0) indicated that **9** should be racemic. Compound **9** was, therefore, identifed as irpexin I, as depicted.

Compound **10** was isolated as a colorless oil. Its molecular formula C_12_H_20_O_2_ was determined on the HR-ESI-MS at *m/z* 197.15361 [M + H]^+^ (calcd for C_12_H_21_O_2_^+^, 197.15361), corresponding to three degrees of unsaturation. In the ^1^H NMR spectrum (Table 1), two methyl group at *δ*_H_ 0.98 (t, *J* = 7.6 Hz, Me-1) and 0.97 (t, *J* = 7.6 Hz, Me-12), one oxygenated proton at *δ*_H_ 3.56 (1H, m, H-10) were identified. The ^13^C and DEPT spectra (Table 2) displayed 12 carbon resonances corresponding to two CH_3_, six CH_2_, one CH (one oxygenated methine at *δ*_C_ 72.9), and three C (one ketone carbon at *δ*_C_ 210.0, two olefinic carbon at *δ*_C_ 173.2 and 142.0) (Table 2). Considering one double bond and one ketone carbon, compound **10** should possess a single ring framework. The gross structure of **10** was deduced by comprehensive analysis of its 2D NMR spectra. In the ^1^H-^1^H COSY spectrum, three fragments were revealed as shown with bold lines in Fig. 3. In the HMBC spectrum, the correlation from *δ*_H_ 2.21 (2H, q, *J* = 7.6 Hz, H-2) to *δ*_C_ 142.0 (s, C-3), 210.0 (s, C-4), and 173.2 (s, C-7), and from *δ*_H_ 2.60 (1H, m, H-8a) and 2.52 (1H, m, H-8b), as well as from *δ*_H_ 2.51 (2H, m, H-6) to C-4 established a five-membered ring by C-3, C-4, C-5, C-6, and C-7. At the same time, the ethyl branch was connected to C-3, and the other branch composed by C8, C9, C10, C11, and C12 was connected to C-7. According to ^1^H-^1^H COSY spectrum, a hydroxy attached to C-10 (*δ*_C_ 72.9) was established. The optical rotation data ([*α*] =  − 9.7) suggested *S* form of C-10. Compound **10** was, therefore, identified as irpexin J, as depicted.

Other known compounds were identified by comparison of their NMR spectroscopic data with the literature, namely irpexlacte B (**11**) [14], irpexlacte C (**12**) [14], irpexlacte D (**13**) [14], 1-(5-(hydroxymethyl)-2-furanyl)-pentanone (**14**) [18], 5-(3-oxopentyl)-2-furancarboxaldehyde (**15**) [19], (1*R*,2*R*)-1-(5-(pent-4-en-1-yl)furan-2-yl)propane-1,2-diol (**16**) [20], (1*R*,2*R*)-1-(5-pentylfuran-2-yl)propane-1,2-diol (**17**) [20], 2,5,8-decanetrione (**21**) [19], 2-butyl-3hydroxy-2-cyclopenten-1-one (**19**) [22].

All new compounds were evaluated for their cytotoxicity against human MCF-7 and Hela cancer cell lines. However, no compounds showed cytotoxicity against two cell lines at the concentration of 10 μM.

## Experimental Section

### General Experimental Procedures

Optical rotations were measured with a Horiba SEPA-300 polarimeter. IR spectra were obtained with a Tenor 27 spectrophotometer using KBr pellets. 1D and 2D spectra were run on a Bruker Avance III 600 MHz spectrometer with TMS as an internal standard. Chemical shifts (*δ*) were expressed in ppm with reference to the solvent signals. Mass spectra were recorded on an Agilent 6200 Q-TOF MS system. Column chromatography (CC) was performed on silica gel (200–300 mesh, Qingdao Marine Chemical Ltd., Qingdao, People’s Republic of China), RP-18 gel (20–45 µm, Fuji Silysia Chemical Ltd., Japan), and Sephadex LH-20 (Pharmacia Fine Chemical Co., Ltd., Sweden). Medium Pressure Liquid Chromatography (MPLC) was performed on a Biotage SP1 equipment, and columns packed with RP-18 gel. Preparative High Performance Liquid Chromatography (prep-HPLC) was performed on an Agilent 1260 liquid chromatography system equipped with Zorbax SB-C18 columns (5 μm, 9.4 mm × 150 mm or 21.2 mm × 150 mm) and a DAD detector. Fractions were monitored by TLC (GF 254, Qingdao Haiyang Chemical Co., Ltd. Qingdao), and spots were visualized by heating silica gel plates sprayed with 10% H_2_SO_4_ in EtOH.

### Fungal Material

*Irpex lacteus* was collected from the Wangtianshu Scenic Area, Xishuangbanna, Yunnan Province in July 2014, and authenticated by Prof. Yu-Cheng Dai of Beijing Forestry University. A voucher specimen of *I. lacteus* was deposited at the Higher Fungi Chemistry Group of School of Pharmaceutical Sciences, South-Central University for Nationalities (No. HFG 201407-SCUN201804.2).

### Fermentation Condition

This strain was cultured on PDA medium for 8 days, and then was cut into small pieces to incubate on solid rice medium. The rice medium was inoculated in 500-mL Erlenmeyer flasks, each containing 100 g of rice medium and 100 mL of water. Flask cultures were put into a high pressure sterilizing pot and sterilized at 121 ℃ for 15 min. Then, the strain of *I. lacteus* was cultured in the rice medium, and two hundred 500-mL Erlenmeyer flasks were dark incubated fixedly at 25 °C for 30 days.

### Extraction and Isolation

The culture of *I. lacteus* in rice medium (20 kg) was extracted five times with Me_2_CO to give a crude extract, which was partitioned into water and EtOAc layers. The EtOAc layer (72 g) was subjected to CC over silica gel (80‒100 mesh) eluted with a solvent system of CHCl_3_/MeOH (from 1:0 to 0:1) to obtain nine fractions (A‒I). Fraction C (6 g) was separated by MPLC over RP-18 silica gel column eluted with MeOH/H_2_O (from 5:95 to 100:0, v/v) to give five sub-fractions (C_1_‒C_5_). Fraction C_1_ was repeatly separated by CC over silica gel eluted with petroleum ether/ethyl acetate (15:1) to gain **18** (4.6 mg), and then purified by prep-HPLC (CH_3_CN/H_2_O from 5:95 to 25:75 in 25 min, v/v) to give **13** (0.8 mg, *t*_R_ = 21.6 min). Fraction C_2_ was separated by CC over Sephadex LH-20 (methanol), and then purified by prep-HPLC (CH_3_CN/H_2_O = 16:84, v/v) to give **2** (2.2 mg, *t*_R_ = 20.1 min), **12** (23.7 mg, *t*_R_ = 18.0 min) and **19** (2.0 mg, *t*_R_ = 24.1 min). Fraction C_3_ was repeatly separated by CC over silica gel eluted with petroleum ether/ ethyl acetate (15:1), and then purified by prep-HPLC (CH_3_CN/H_2_O from 8:92 to 20:80 in 40 min, v/v) to give** 7** (4.5 mg, *t*_R_ = 20.8 min) and **15** (0.6 mg, *t*_R_ = 17.3 min). Fraction C_4_ was separated by CC over Sephadex LH-20 (methanol), and then purified by prep-HPLC (MeOH/H_2_O from 25:75 to 55:45 in 30 min, v/v) to give **1** (24.8 mg, *t*_R_ = 22.6 min) and **4** (2.5 mg, *t*_R_ = 17.4 min).

Fraction D (8 g) was separated by MPLC over RP-18 silica gel column eluted with MeOH/H_2_O (from 5:95 to 100:0, v/v) to give eleven sub-fractions (D_1_‒D_11_). Fraction D_2_ was purified by prep-HPLC (CH_3_OH/H_2_O = 26:74, v/v) to give **3** (2.5 mg, *t*_R_ = 18.2 min) and **9** (1.0 mg, *t*_R_ = 14.6 min). Fraction D_3_ was separated by CC over Sephadex LH-20 (methanol), and then purified by prep-HPLC (CH_3_CN/H_2_O = 21:79, v/v) to give **6** (2.4 mg, *t*_R_ = 20.6 min) and **10** (5.0 mg, *t*_R_ = 25.0 min), and by prep-HPLC (CH_3_CN/H_2_O = 26:73, v/v) to give **11** (14.1 mg, *t*_R_ = 16.2 min) and **14** (2.4 mg, *t*_R_ = 18.9 min). Fraction D_4_ was repeatly separated by CC over silica gel eluted with petroleum ether/acetone (10:1), and then purified by prep-HPLC (CH_3_CN/H_2_O = 32:68, v/v) to give **7** (3.3 mg, *t*_R_ = 15.4 min). Fraction D_6_ was separated by CC over silica gel eluted with petroleum ether/acetone (10:1), and then purified by prep-HPLC (CH_3_CN/H_2_O = 32:68, v/v) to give **16** (2.2 mg, *t*_R_ = 17.5 min) and **17** (12.8 mg, *t*_R_ = 19.8 min). Fraction E (7.2 g) was separated by MPLC over RP-18 eluted with MeOH/H_2_O (from 5/95 to 100/0, v/v) to give five subfractions (E_1_‒E_5_). Fraction E_3_ was subjected to CC over silica gel (200–300 mesh) and Sephadex LH-20 (methanol), and then purified by prep-HPLC (CH_3_CN/H_2_O from 20:80 to 70:30 in 20 min, v/v) to give **8** (3.2 mg, *t*_R_ = 8.9 min).

### Spectroscopic Data of Compounds

#### Irpexin A (**1**)

Colorless oil; [*α*]_D_^27^ + 44.8 (*c* 0.5, MeOH); IR (KBr) ν_max_: 3444, 2937, 1668, 1517, 1259, 1097, 1029, 968 cm^−1^; ^1^H NMR (600 MHz, CDCl_3_) and ^13^C NMR (150 MHz, CDCl_3_) data, see Tables 1 and 2; HR-ESI-MS: *m/z* 183.10158 [M + H]^+^ (calcd for C_10_H_15_O_3_^+^, 183.10157).

#### Irpexin B (**2**)

Colorless oil; [*α*]_D_^27^ − 38.0 (*c* 0.5, MeOH); IR (KBr) *ν*_*max*_: 1670, 1508 cm^−1^; ^1^H NMR (600 MHz, CDCl_3_) and ^13^C NMR (150 MHz, CDCl_3_) data, see Tables 1 and 2; HR-ESI-MS: *m/z* 181.08589 [M + H]^+^ (calcd for C_10_H_13_O_3_^+^, 181.08592).

#### Irpexin C (**3**)

Colorless oil; [*α*]_D_^27^ − 16.1 (*c* 0.27, MeOH); UV (MeOH) λ_max_ (log ε) 225 (3.31) and 275 (3.12) nm. IR (KBr) *ν*_*max*_: 3338, 2943, 2831, 1452, 1114, 1031 cm^−1^; ^1^H NMR (600 MHz, CDCl_3_) and ^13^C NMR (150 MHz, CDCl_3_) data, see Tables 1 and 2; HR-ESI-MS: *m/z* 207.09825 [M + Na]^+^ (calcd for C_10_H_16_O_3_Na^+^, 207.09917).

#### Irpexin D (**4**)

Colorless oil; [*α*]_D_^27^ + 37.5 (*c* 0.5, MeOH); IR (KBr) *ν*_*max*_: 3504, 1635, 1653, 1246 cm^−1^; ^1^H NMR (600 MHz, CDCl_3_) and ^13^C NMR (150 MHz, CDCl_3_) data, see Tables 1 and 2; HR-ESI-MS: *m/z* 251.12544 [M + Na]^+^ (calcd for C_12_H_20_O_4_Na^+^, 251.12538).

#### Irpexin E (**5**)

Colorless oil; [*α*]_D_^27^ + 14.6 (*c* 0.1, MeOH); IR (KBr) *ν*_*max*_: 3348, 2943, 2831, 1452, 1114, 1031 cm^−1^; ^1^H NMR (600 MHz, CDCl_3_) and ^13^C NMR (150 MHz, CDCl_3_) data, see Tables 1 and 2; HR-ESI-MS: *m/z* 211.13280 [M + H]^+^ (calcd for C_12_H_19_O_3_^+^, 211.13287).

#### Irpexin F (**6**)

Colorless oil; [*α*]_D_^27^ ‒ 14.3 (*c* 0.1, MeOH); IR (KBr) *ν*_*max*_: 3336, 2943, 2831, 1454, 1114, 1031 cm^−1^; ^1^H NMR (600 MHz, CDCl_3_) and ^13^C NMR (150 MHz, CDCl_3_) data, see Tables 1 and 2; HR-ESI-MS: *m/z* 233.11481 [M + Na]^+^ (calcd for C_12_H_18_O_3_Na^+^, 233.11482).

#### Irpexin G (**7**)

Colorless oil; [*α*]_D_^27^ ‒ 14.0 (*c* 0.5, MeOH); IR (KBr) *ν*_*max*_: 3564, 2941, 1653, 1259 cm^−1^; ^1^H NMR (600 MHz, CDCl_3_) and ^13^C NMR (150 MHz, CDCl_3_) data, see Tables 1 and 2; HR-ESI-MS: *m/z* 225.11212 [M + H]^+^ (calcd for C_12_H_17_O_4_^+^, 225.11214).

#### Irpexin H (**8**)

Colorless oil; [*α*]_D_^27^ ‒ 17.9 (*c* 0.2, MeOH); UV (MeOH) λ_max_ (log ε) 210 (3.48) and 280 (2.79) nm. IR (KBr) *ν*_*max*_: 3398, 2949, 1651, 1450, 1024 cm^−1^; ^1^H NMR (600 MHz, methanol-*d*_4_) and ^13^C NMR (150 MHz, methanol-*d*_4_) spectroscopic data, see Tables 1 and 2; HR-ESI–MS: m/z 239.12790 [M + H]^+^ (calcd for C_13_H_19_O_4_^+^, 239.12779).

#### Irpexin I (**9**)

Colorless oil; [α]_D_^27^ 0 (*c* 0.67, MeOH); UV (MeOH) λ_max_ (log ε) 210 (2.20) nm. IR (KBr) *ν*_*max*_: 3338, 2943, 2831, 1452, 1114, 1031 cm^−1^; ^1^H NMR (600 MHz, CDCl_3_) and ^13^C NMR (150 MHz, CDCl_3_) data, see Tables 1 and 2; HR-ESI-MS: *m/z* 221.07843 [M + Na]^+^ (calcd for C_10_H_14_O_4_Na^+^, 221.07843).

##### Irpexin J (**10**)

Colorless oil; [α]_D_^27^ ‒ 9.7 (*c* 0.12, MeOH); UV (MeOH) λ_max_ (log ε) 235 (3.18) nm. IR (KBr) *ν*_*max*_: 3338, 2943, 2831, 1647, 1452, 1114, 1031 cm^−1^; ^1^H NMR (600 MHz, CDCl_3_) and ^13^C NMR (150 MHz, CDCl_3_) spectroscopic data, see Tables 1 and 2; HR-ESI–MS: *m/z* 197.15361 [M + H]^+^ (calcd for C_12_H_21_O_2_^+^, 197.15361).

### Mosher Reaction

Compound **1** (25 μmol) was added to a 10 mL round bottom flask, then 2.5 mL of dichloromethane and 50 μmol of pyridine were added. Finally, 50 μmol of (*R*) or (*S*) -MTPA-Cl was added dropwise, which was detected by the thin-layer chromatography. After 3 h of stirring at room temperature, the reaction was terminated, and the reaction solution was washed twice with 1 M diluted hydrochloric acid. The organic phase was concentrated and separated by HPLC to obtain compounds **1a** and **1b** respectively.

### Cytotoxicity Assay

Two human cancer cell lines, breast cancer MCF-7 and Hela, were used in the cytotoxic assay. All the cells were cultured in RPMI-1640 or DMEM medium (Hyclone, USA), supplemented with 10% fetal bovine serum (Hyclone, USA) in 5% CO_2_ at 37 ℃. The cytotoxicity assay was performed according to the MTT (3-(4,5-dimethylthiazol-2-yl)-2,5-diphenyl tetrazolium bromide) method in 96-well microplates [23]. Briefly, 100 µL adherent cells were seeded into each well of 96-well cell culture plates and allowed to adhere for 12 h before drug addition, while suspended cells were seeded just before drug addition with initial density of 1 × 10^5^ cells/mL. Each tumor cell line was exposed to the test compound at concentrations of 0.0625, 0.32, 1.6, 8, and 40 μM in triplicates for 48 h, with taxol as a positive control. After compound treatment, cell viability was detected and cell growth curve was graphed. IC_50_ value was calculated by Reed and Muench’s method [24].

## Electronic supplementary material

Below is the link to the electronic supplementary material.

Supplementary file1 (PDF 8001 KB)
