# Supplementary material for: Furan Derivatives and Polyketides from the Fungus Irpex lacteus
Source: Nat Prod Bioprospect. 2020 Nov 12;11(2):215–22. doi: 10.1007/s13659-020-00282-w (PMC7981371; doi:10.1007/s13659-020-00282-w)
Supplement: Supplementary file 1 — Supplementary file1 (PDF 8001 KB) [file 13659_2020_282_MOESM1_ESM.pdf]

Supporting Information for

Furan Derivatives and Polyketides from the Fungus *Irpex lacteus*

Meng Wang[1], Zheng-Hui Li[1], Masahiko Isaka[2], Ji-Kai Liu[1]*, Tao Feng[1]*

[1]School of Pharmaceutical Sciences, South-Central University for Nationalities, Wuhan 430074, People's Republic of China

[2]National Center for Genetic Engineering and Biotechnology (BIOTEC), 113 Thailand Science Park, Pathumthani 12120, Thailand

*Corresponding author: tfeng@mail.scuec.edu.cn (T. Feng); liujikai@mail.scuec.edu.cn (J.K. Liu)

Contents

Section S1. NMR and MS spectra for Compounds 1–6.

Figure S1-S7. NMR and MS spectra of irpexin A (**1**).

Figure S8. NMR and MS spectra of **1a**.

Figure S9. NMR and MS spectra of **1b**.

Figure S10-S16. NMR and MS spectra of irpexin B (**2**).

Figure S17-S23. NMR and MS spectra of irpexin C (**3**).

Figure S24-S30. NMR and MS spectra of irpexin D (**4**).

Figure S31-S37. NMR and MS spectra of irpexin E (**5**).

Figure S38-S44. NMR and MS spectra of irpexin F (**6**).

Figure S45-S51. NMR and MS spectra of irpexin G (**7**).

Figure S52-S58. NMR and MS spectra of irpexin H (**8**).

Figure S59-S65. NMR and MS spectra of irpexin I (**9**).

Figure S66-S72. NMR and MS spectra of irpexin J (**10**).

Section S2. Specific Optical Rotation Calculation for compound 6.

Section S1. NMR and MS spectra for Compounds 1–6.

Figure S1. ^{1}H NMR (600 MHz, $CDCl_3$) spectrum of compound **1**

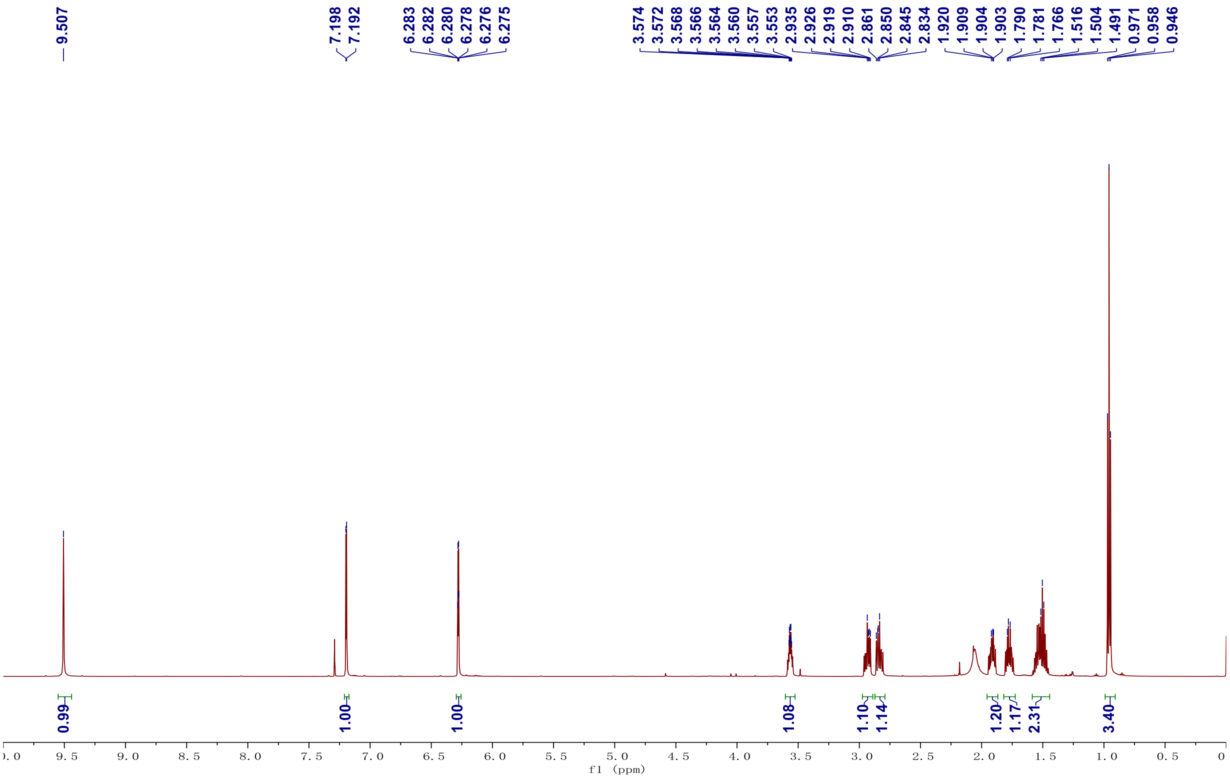

Figure S2. ^{13}C NMR (150 MHz, $CDCl_3$) spectrum of compound **1**

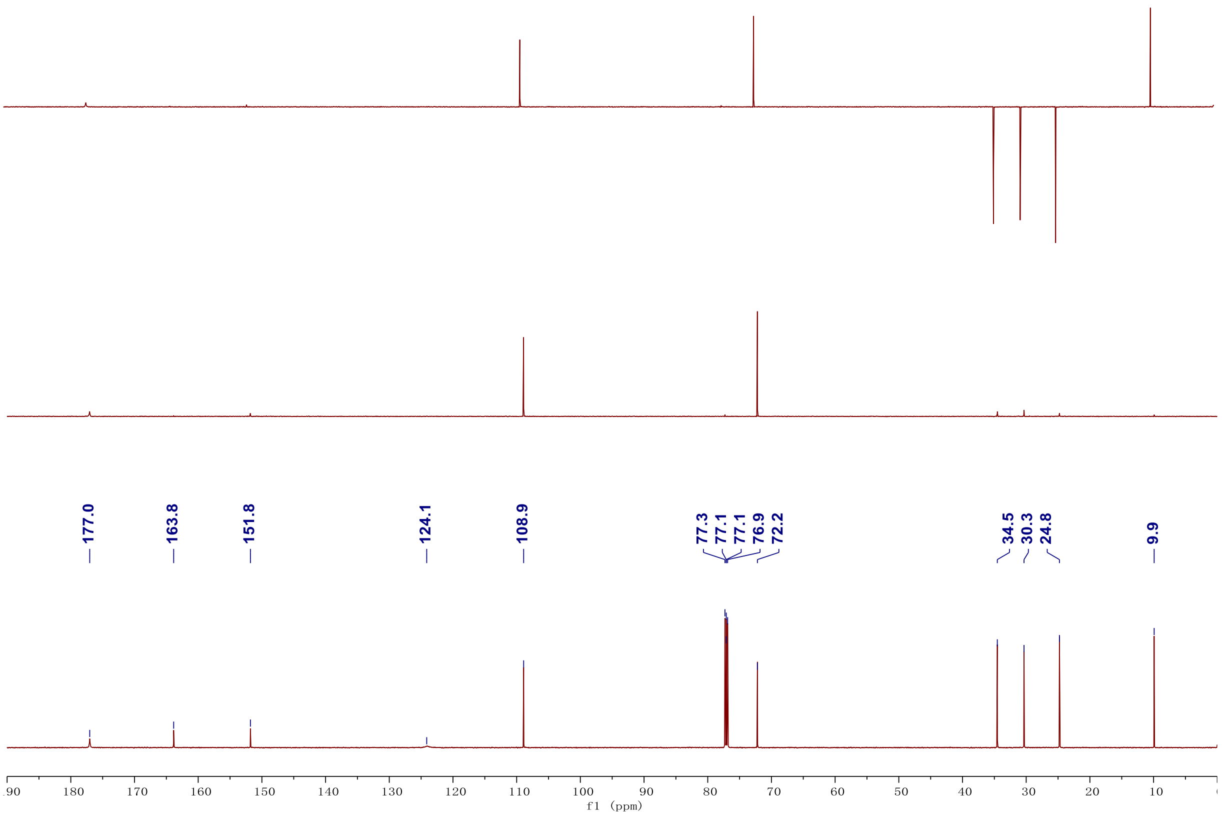

Figure S3. HSQC spectrum of compound **1**

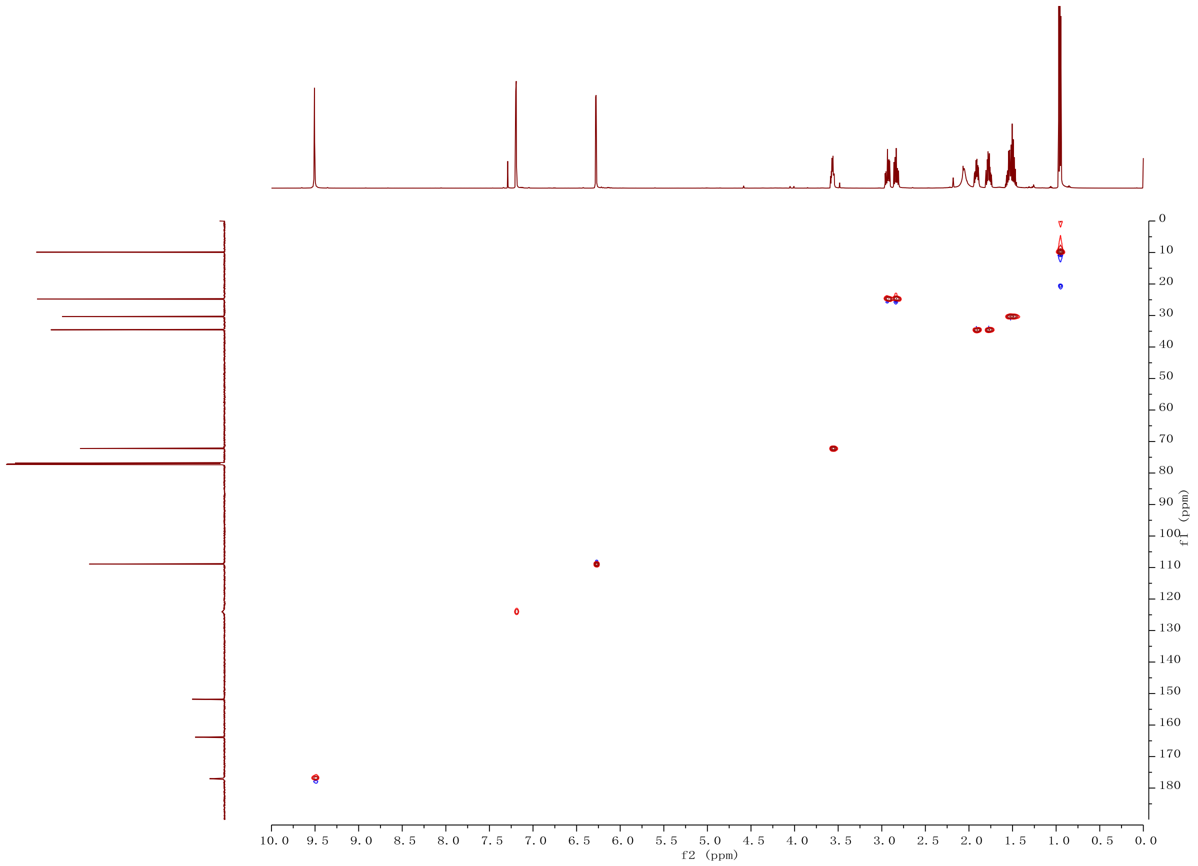

Figure S5. HMBC spectrum of compound **1**

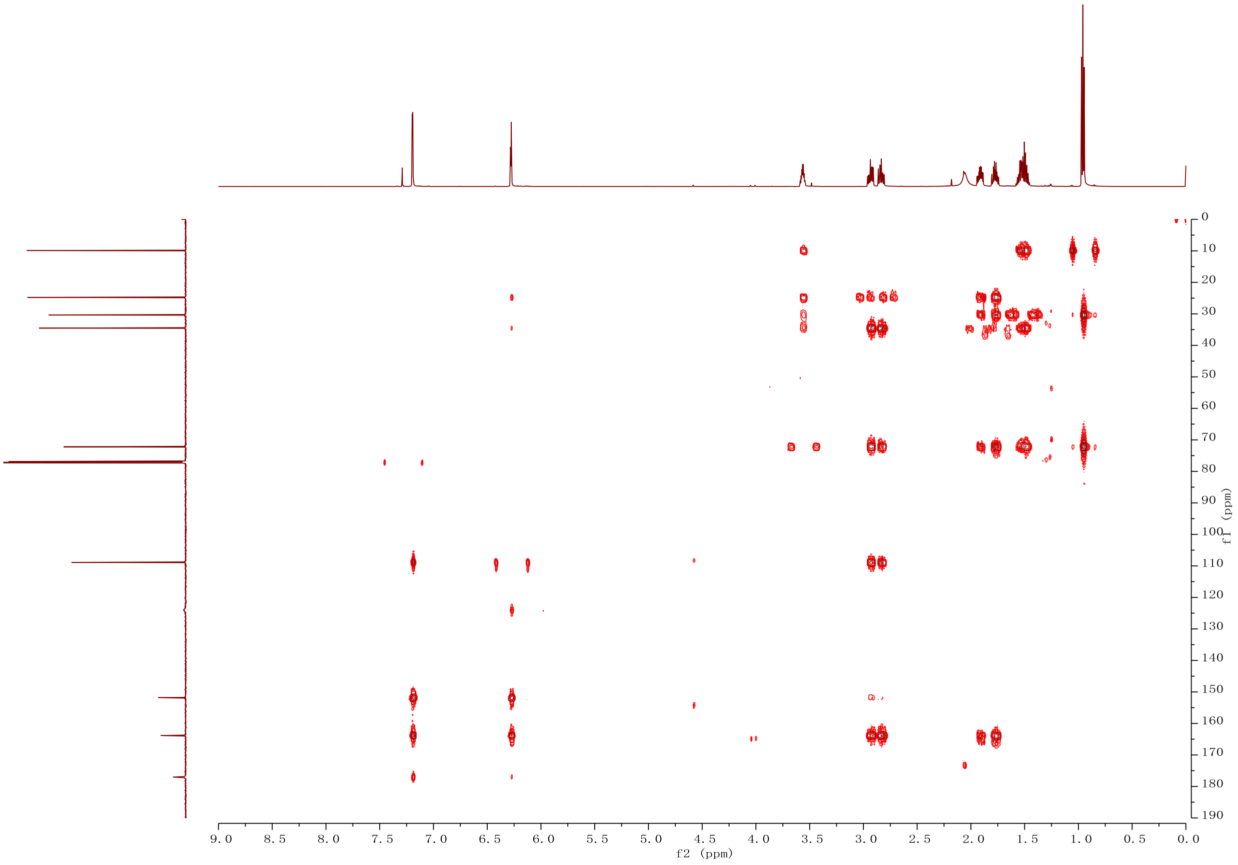

Figure S4. COSY spectrum of compound **1**

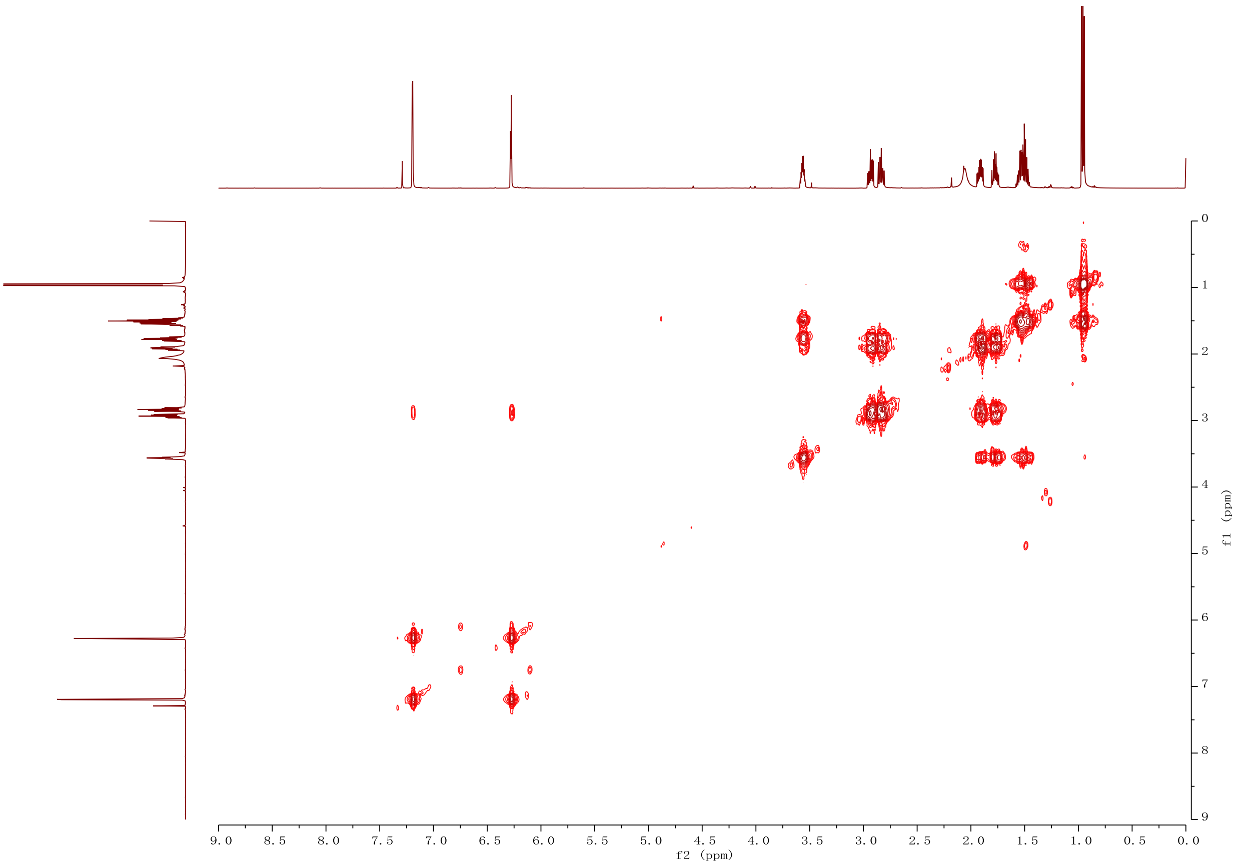

Figure S6. ROESY spectrum of compound **1**

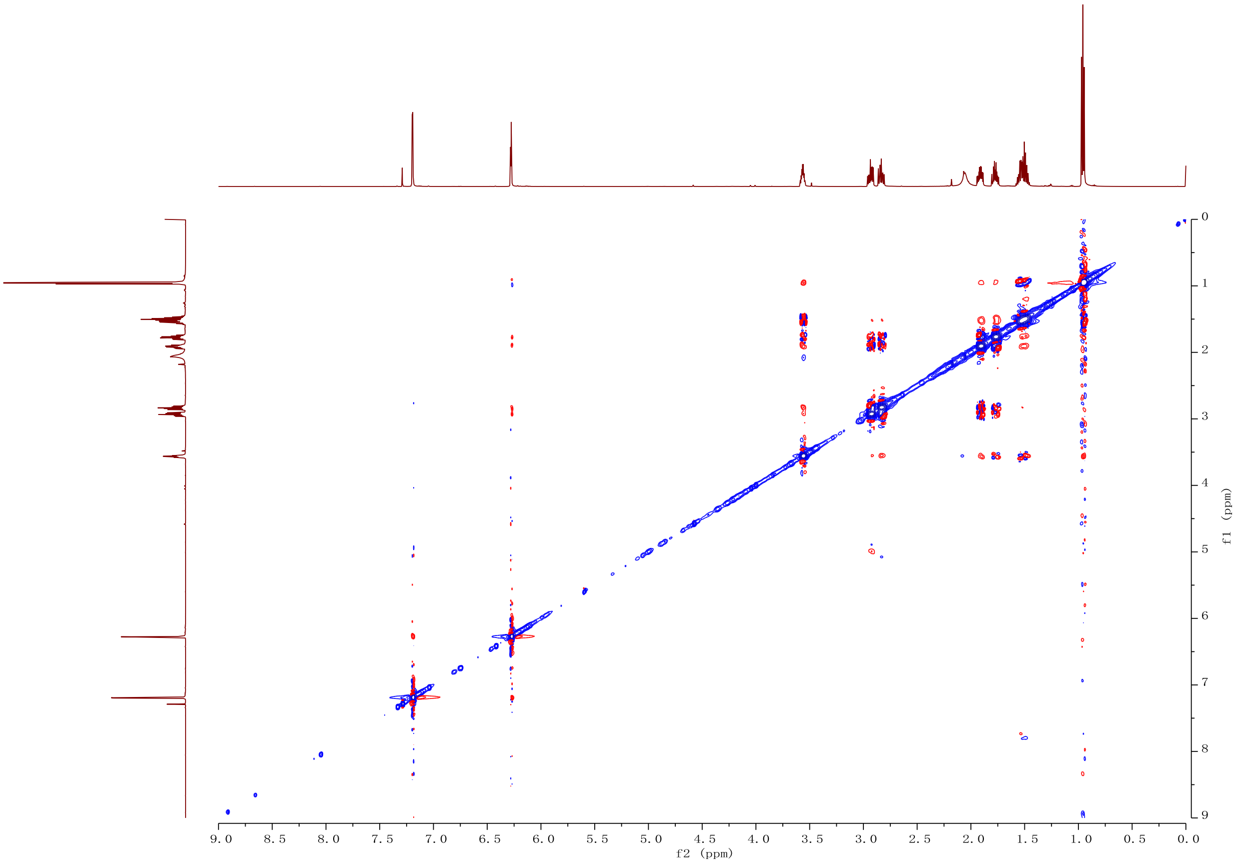

Figure S7. HR-ESI-MS of compound **1**

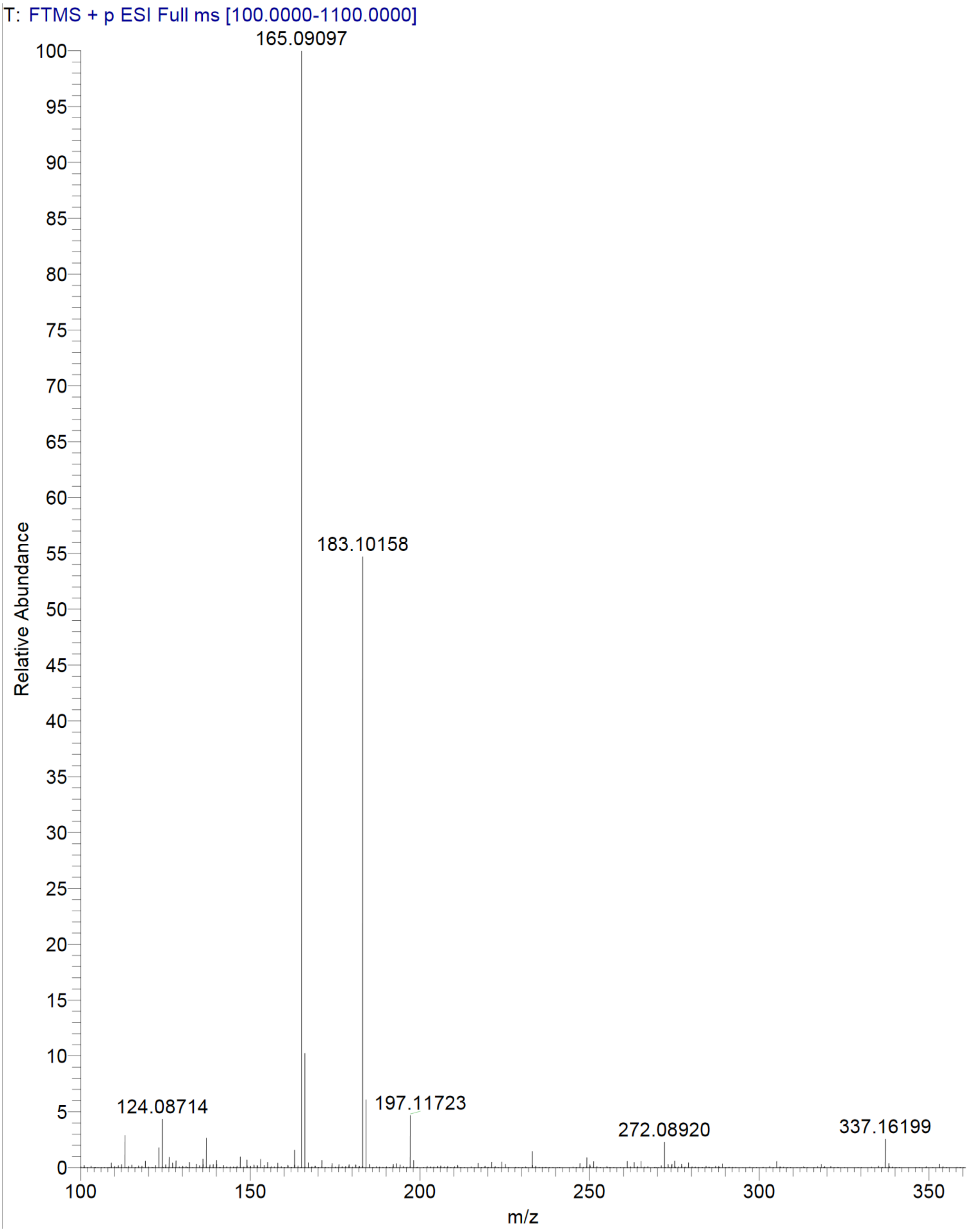

Figure S8. ^{1}H NMR (600 MHz, $CDCl_3$) spectrum of compound **1a**

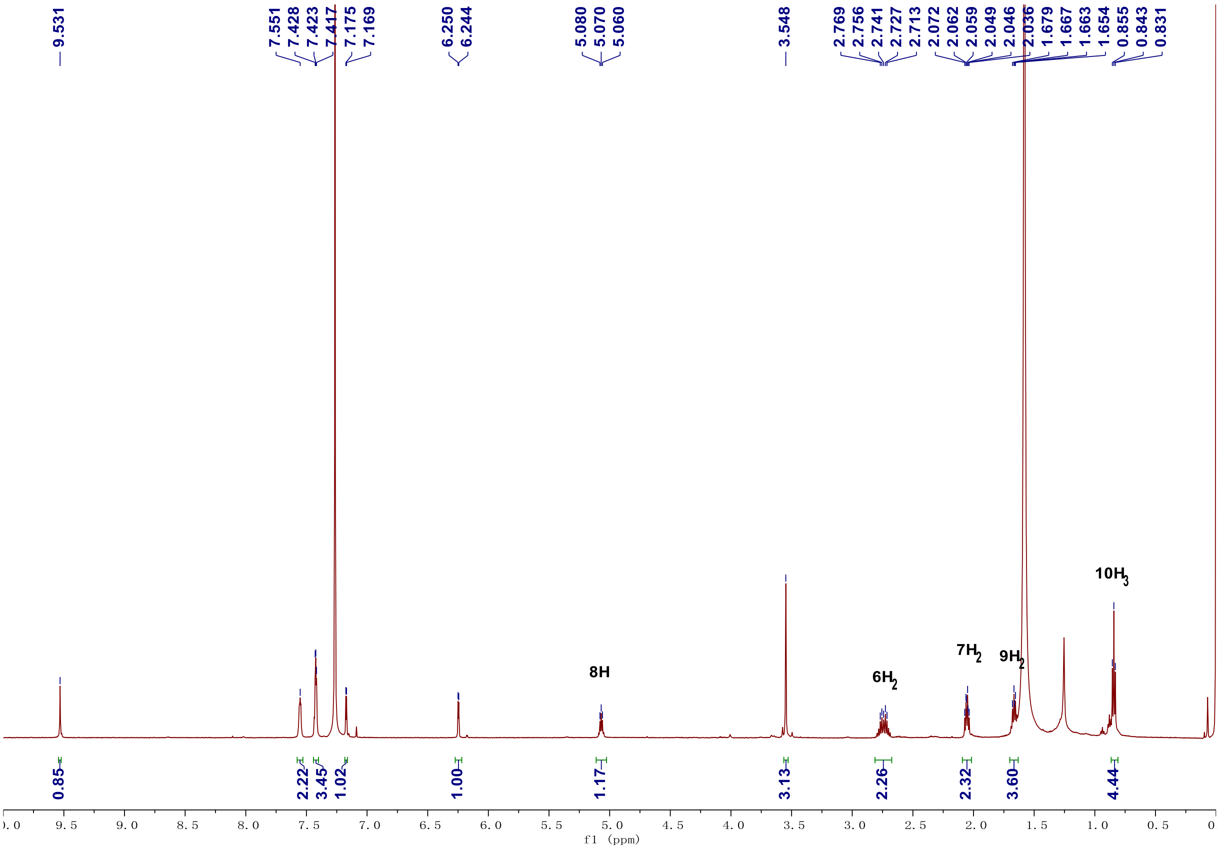

Figure S9. ^{1}H NMR (600 MHz, $CDCl_3$) spectrum of compound **1b**

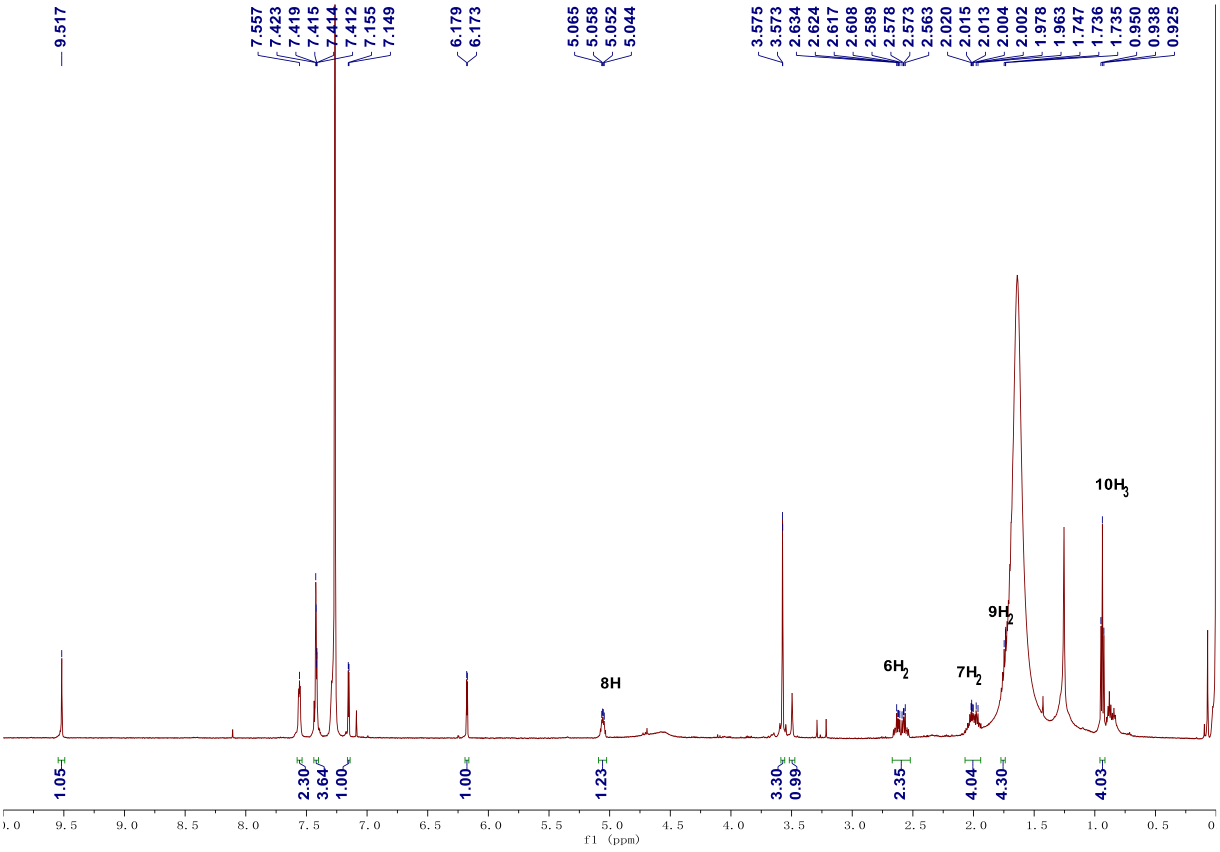

Figure S10. ^{1}H NMR (600 MHz, $CDCl_3$) spectrum of compound **2**

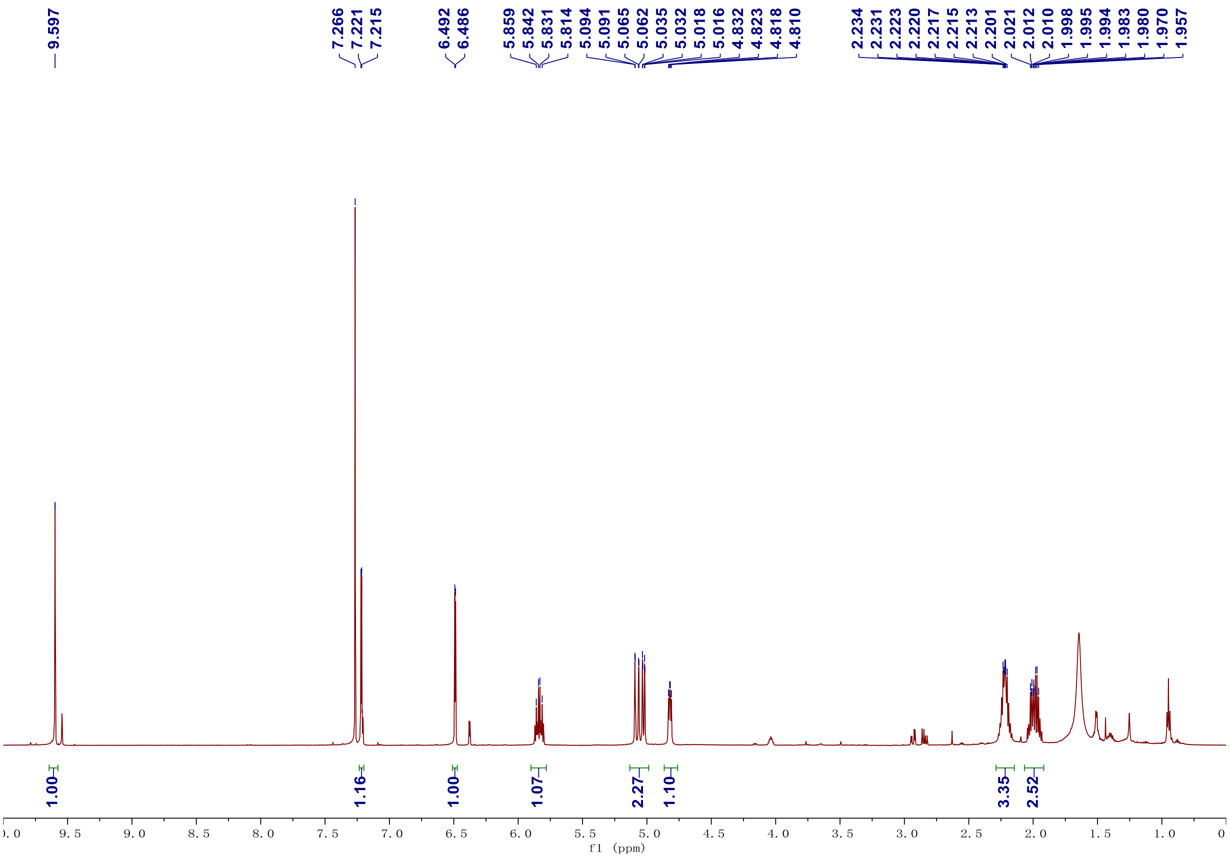

Figure S11. ^{13}C NMR (150 MHz, $CDCl_3$) spectrum of compound **2**

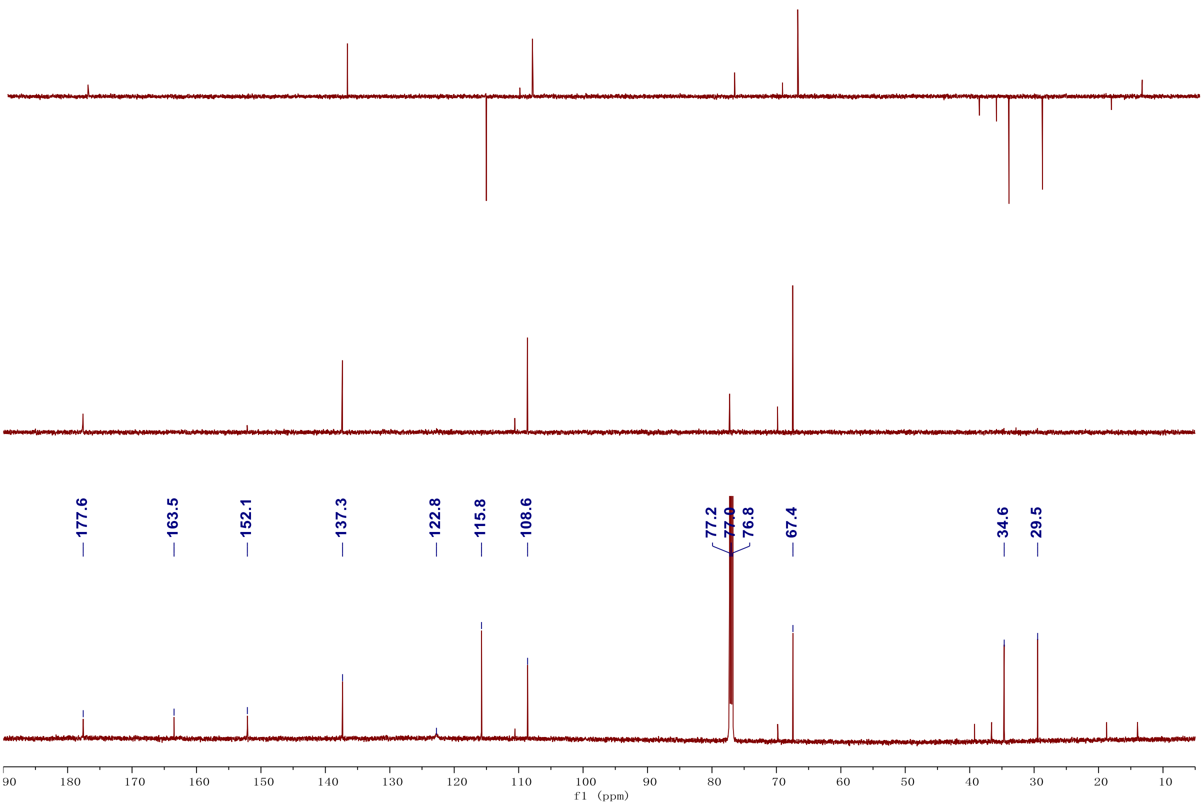

Figure S12. HSQC spectrum of compound **2**

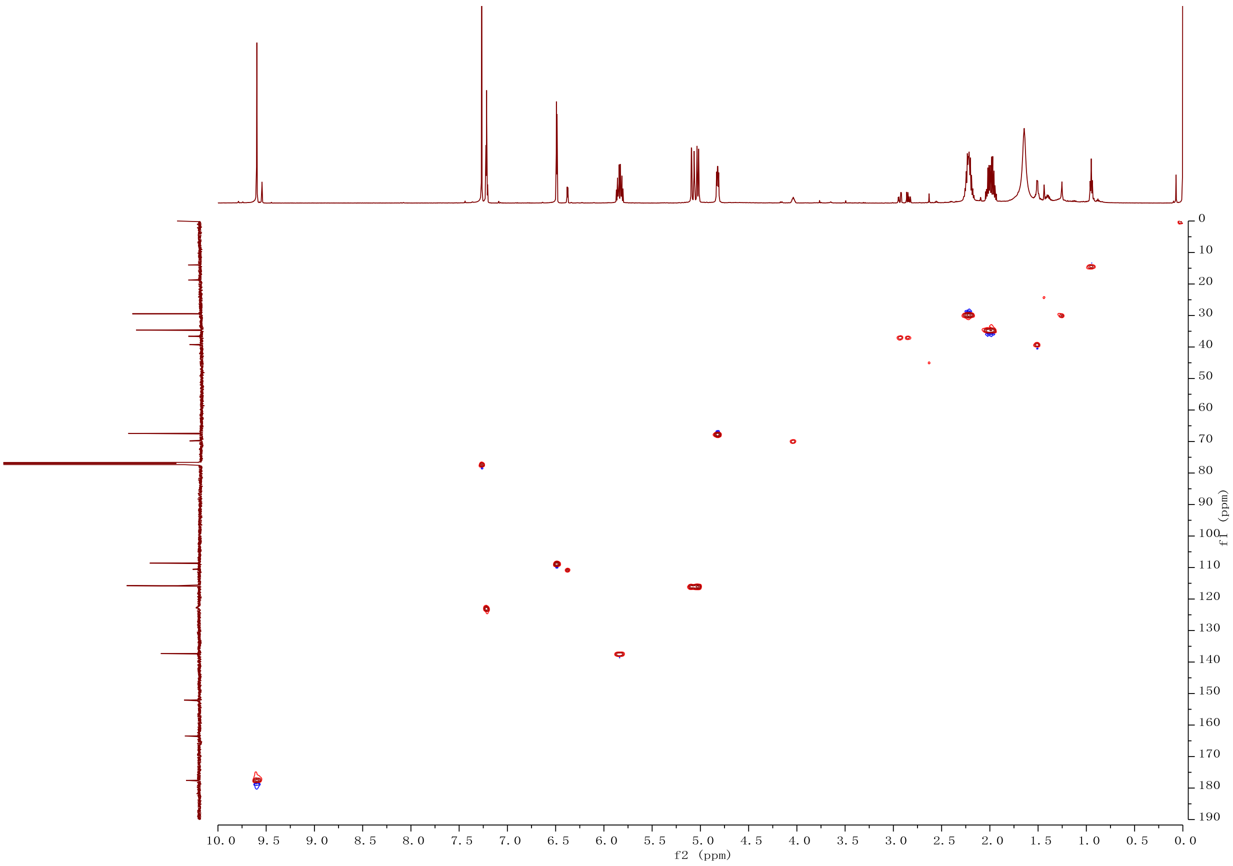

Figure S13. HMBC spectrum of compound **2**

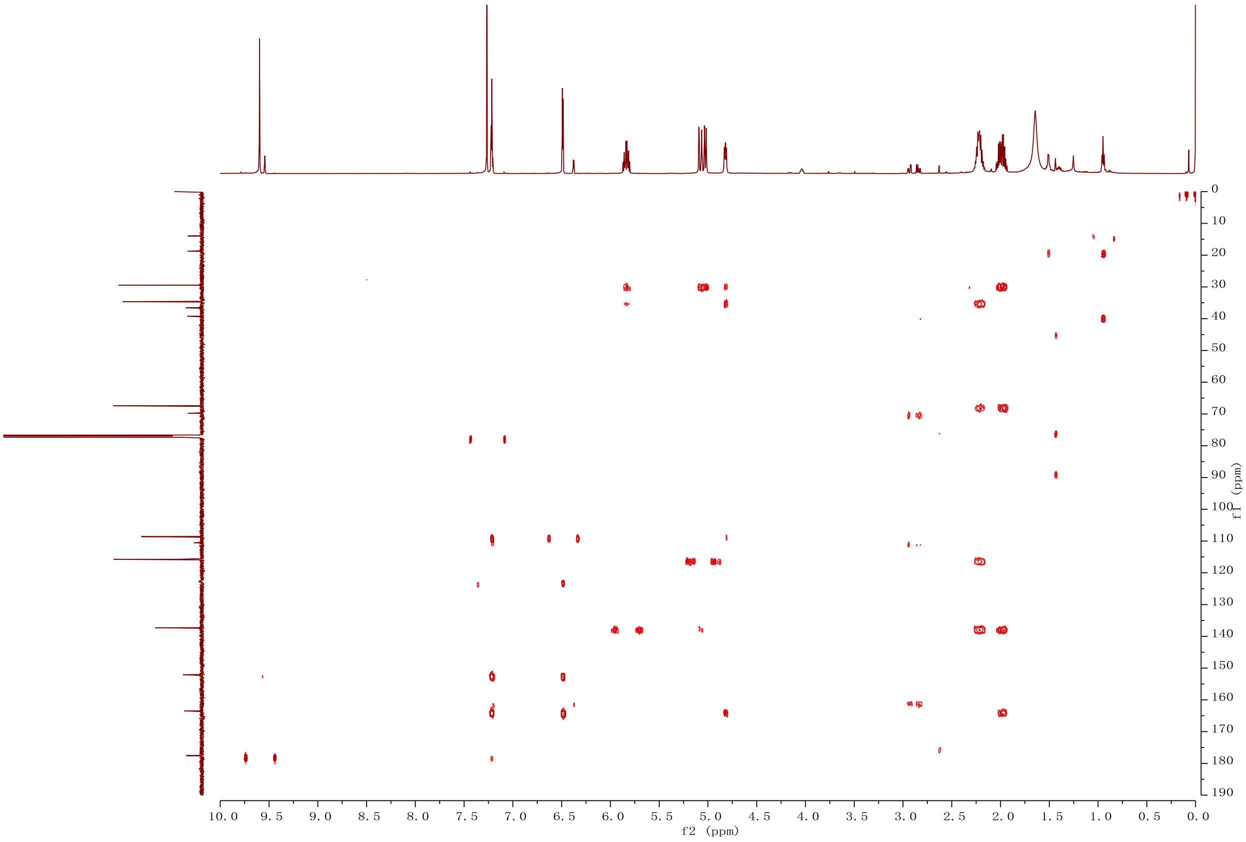

Figure S14. COSY spectrum of compound **2**

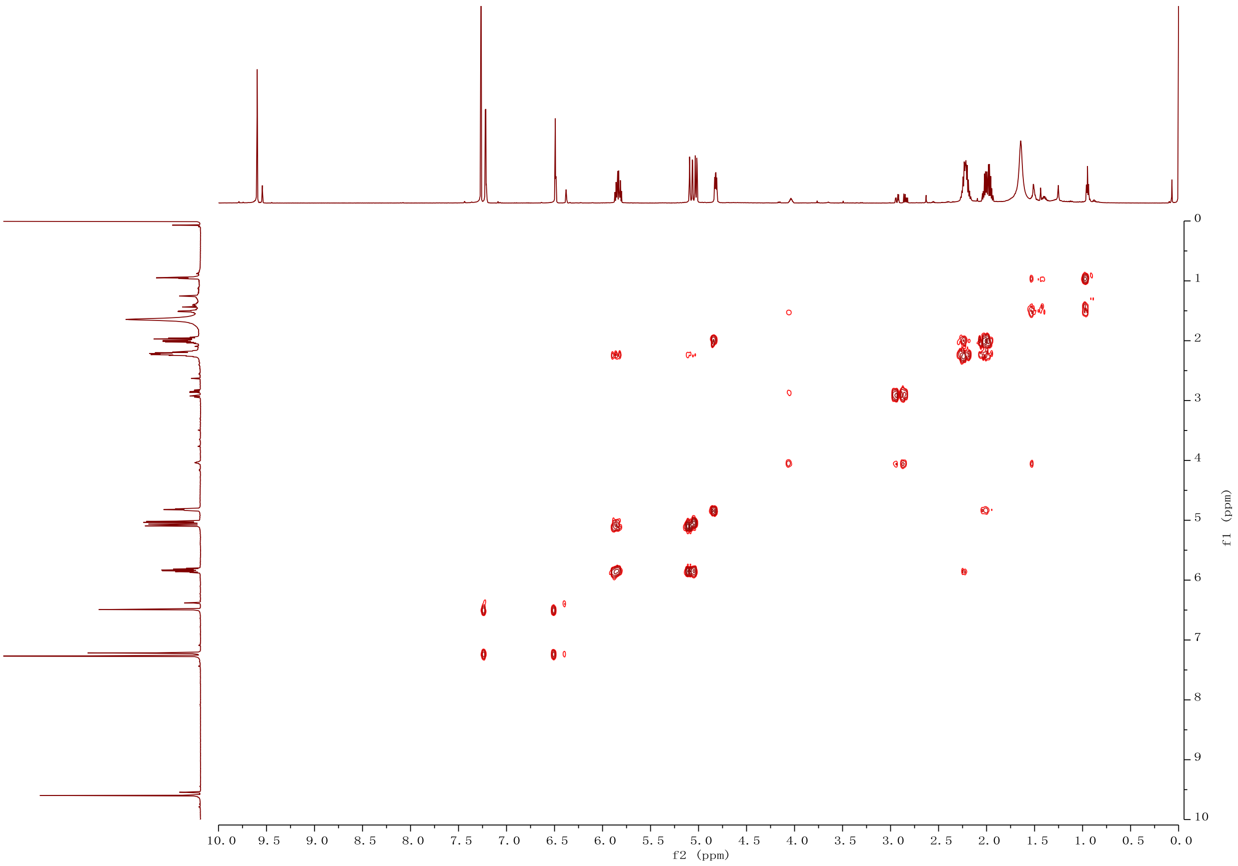

Figure S15. ROESY spectrum of compound **2**

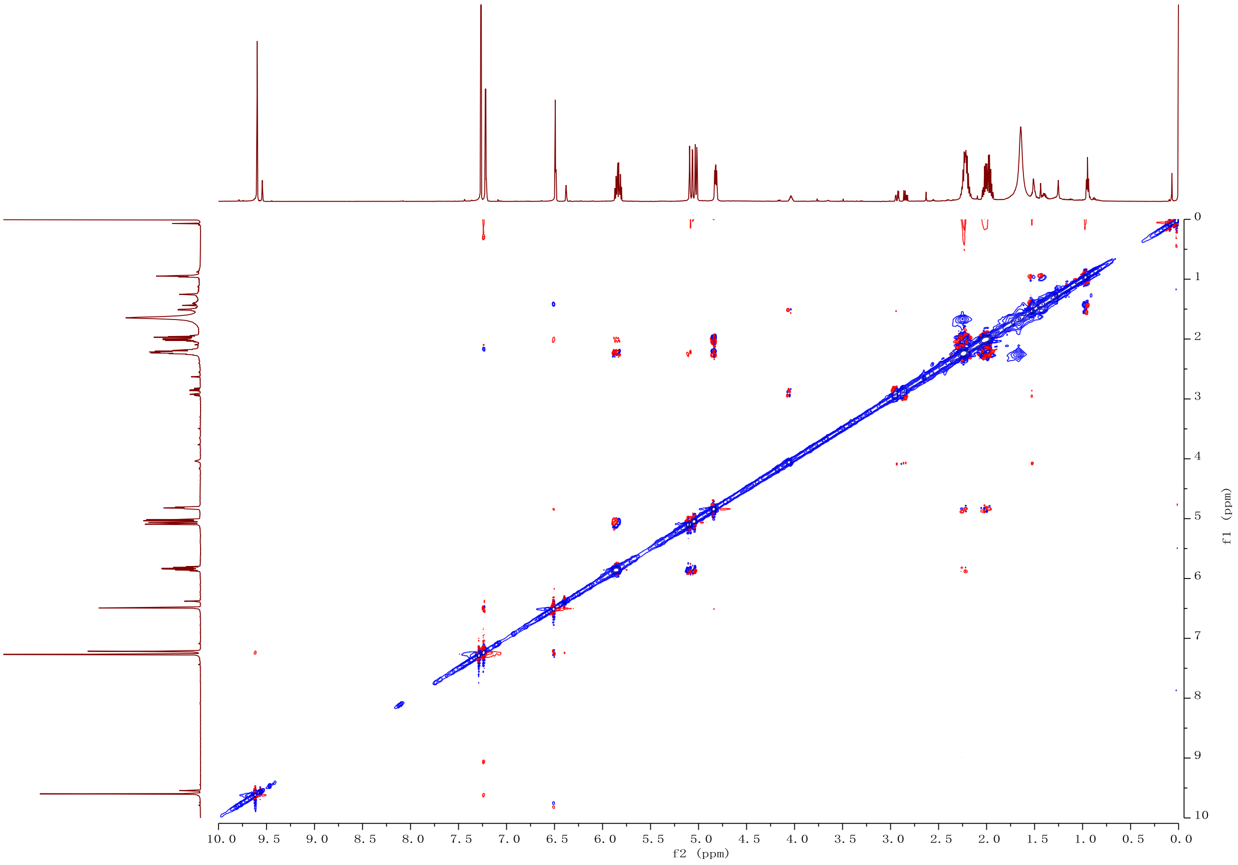

Figure S16. HR-ESI-MS of compound **2**

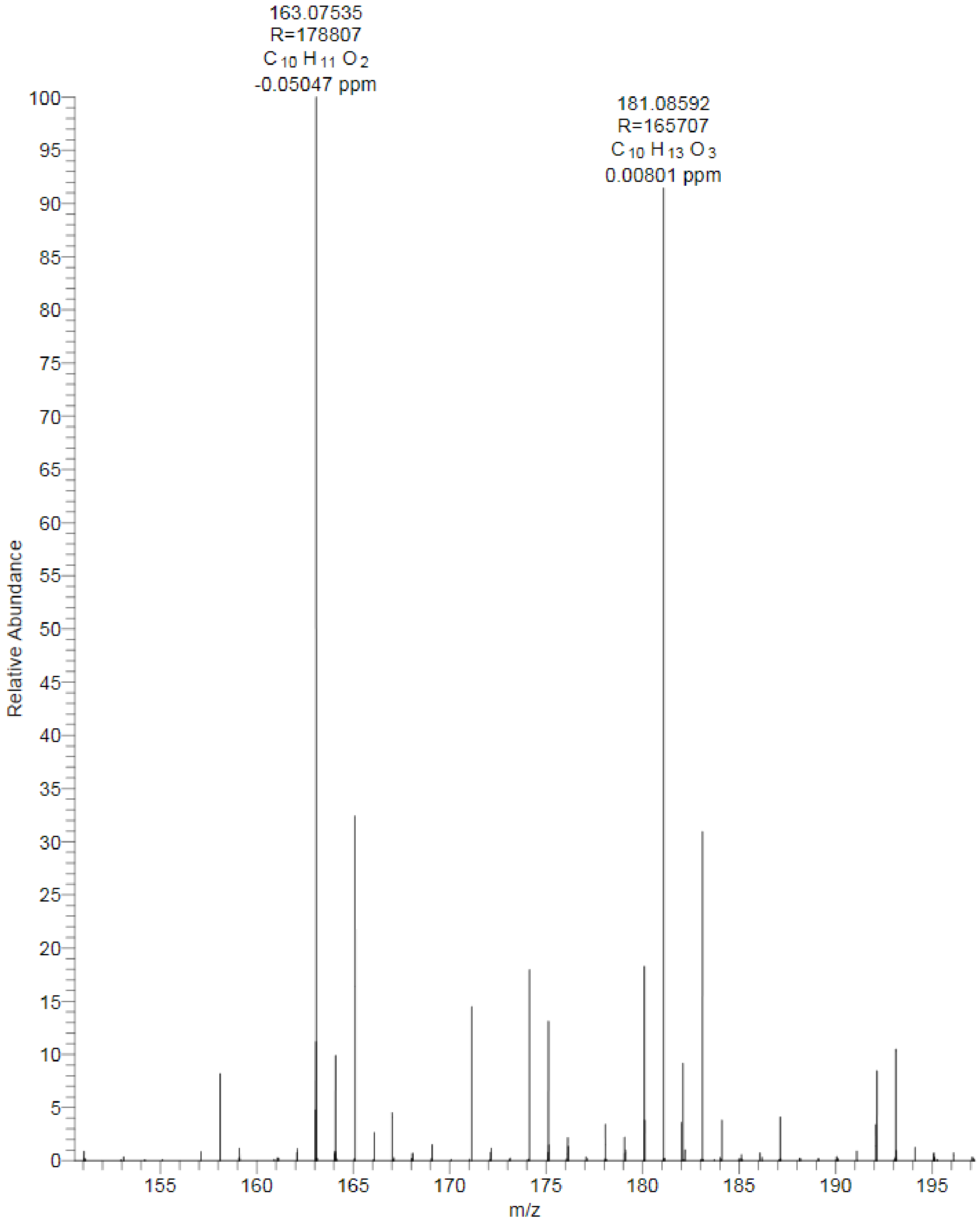

Figure S17. ^{1}H NMR (600 MHz, $CDCl_3$) spectrum of compound **3**

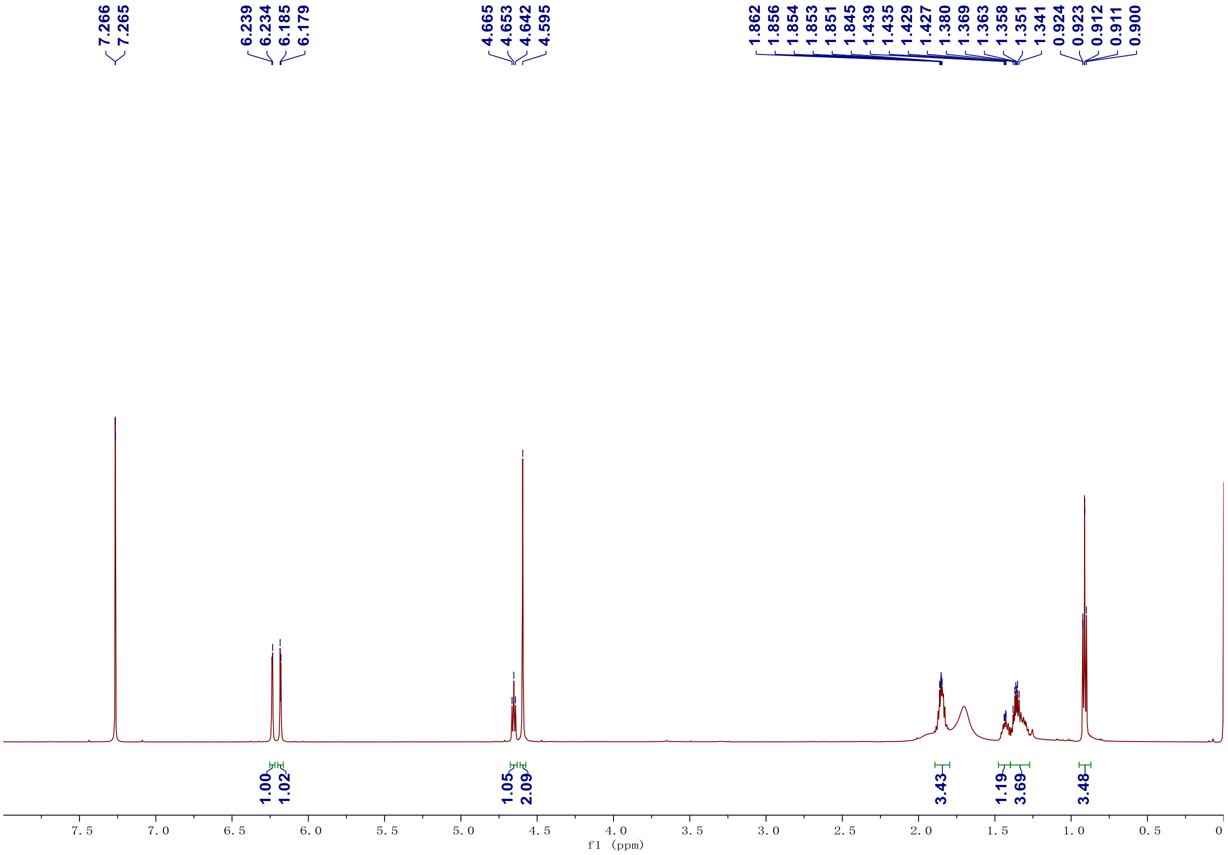

Figure S18. ^{13}C NMR (150 MHz, $CDCl_3$) spectrum of compound **3**

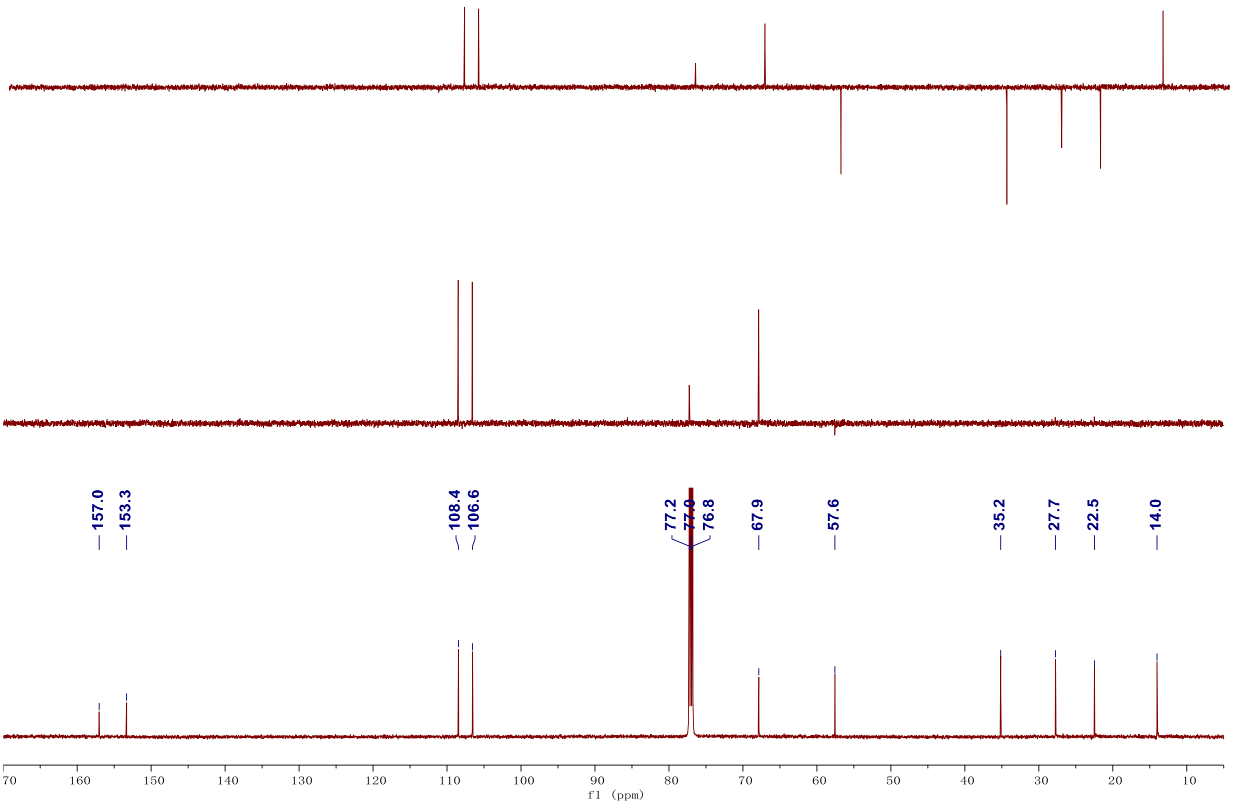

Figure S19. HSQC spectrum of compound **3**

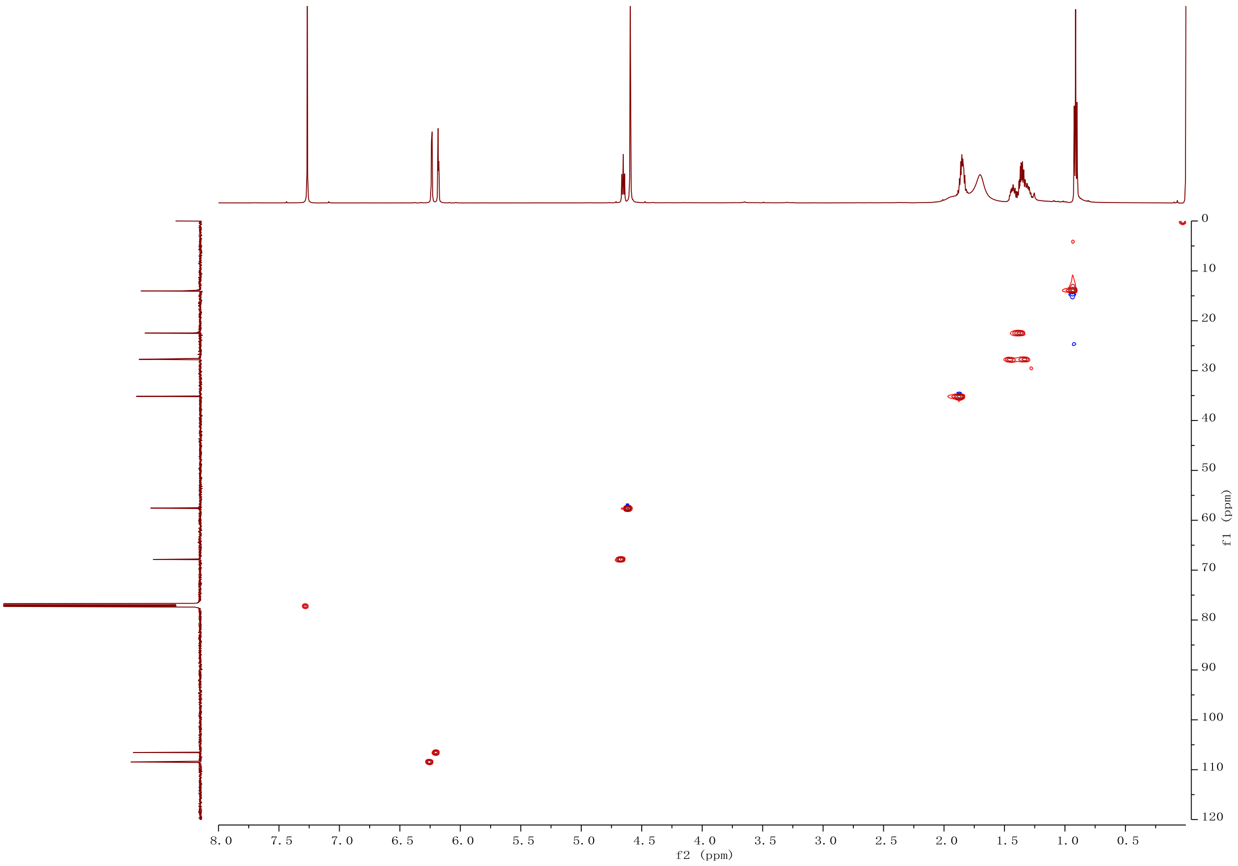

Figure S20. HMBC spectrum of compound **3**

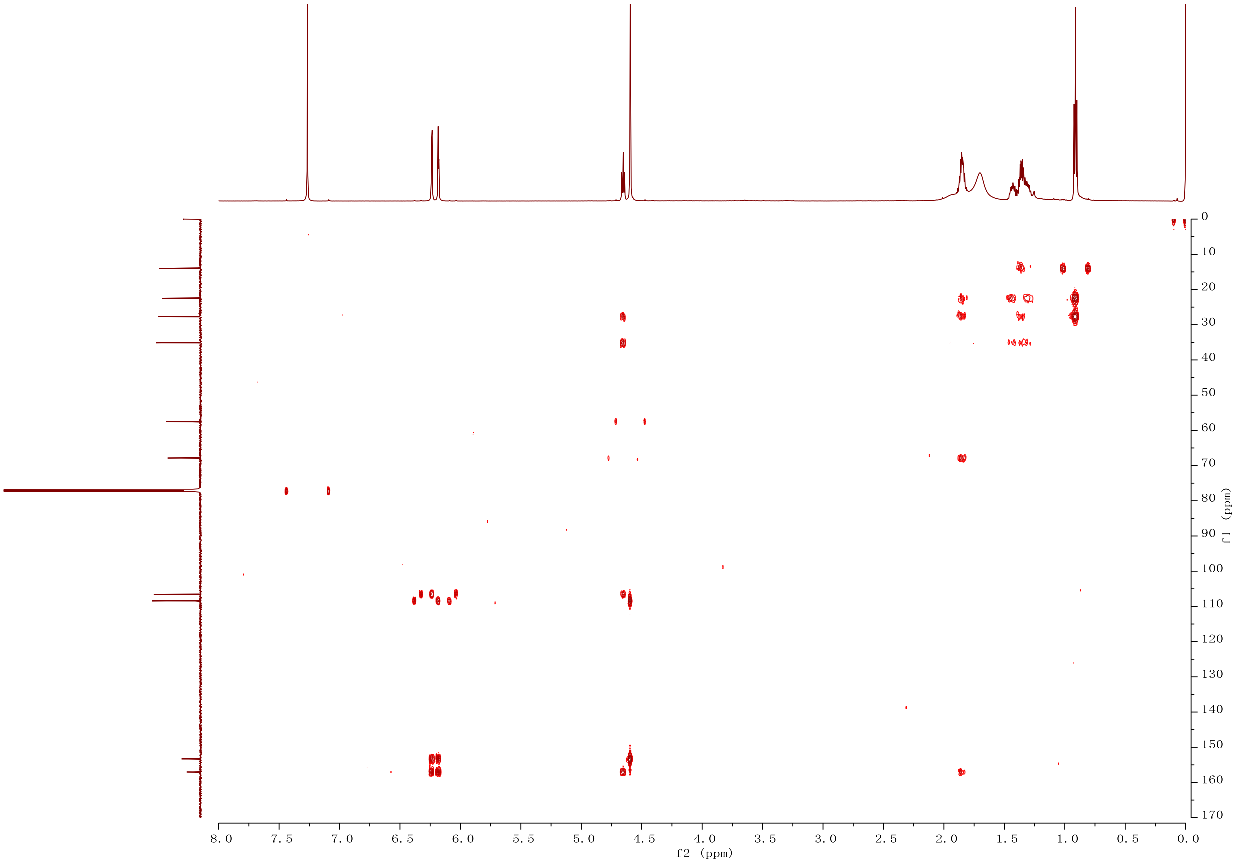

Figure S21. COSY spectrum of compound **3**

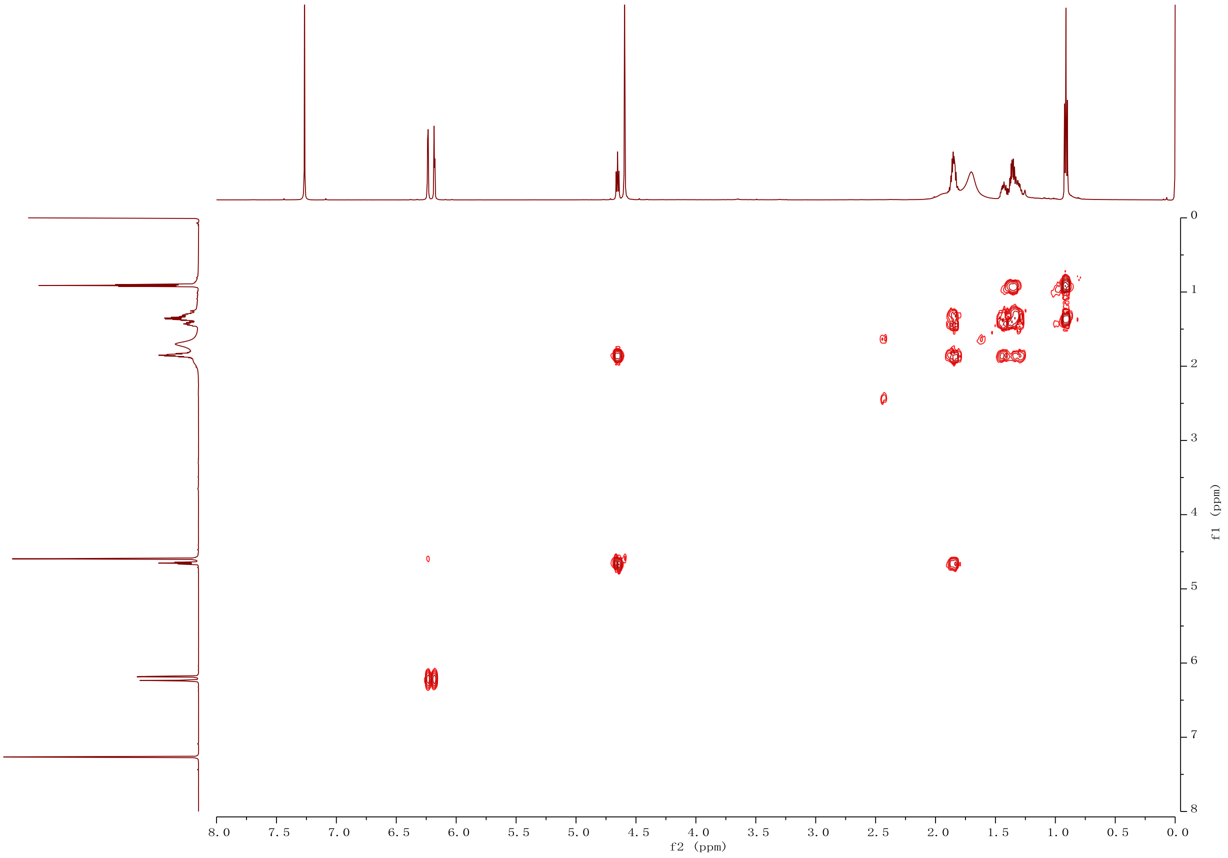

Figure S22. ROESY spectrum of compound **3**

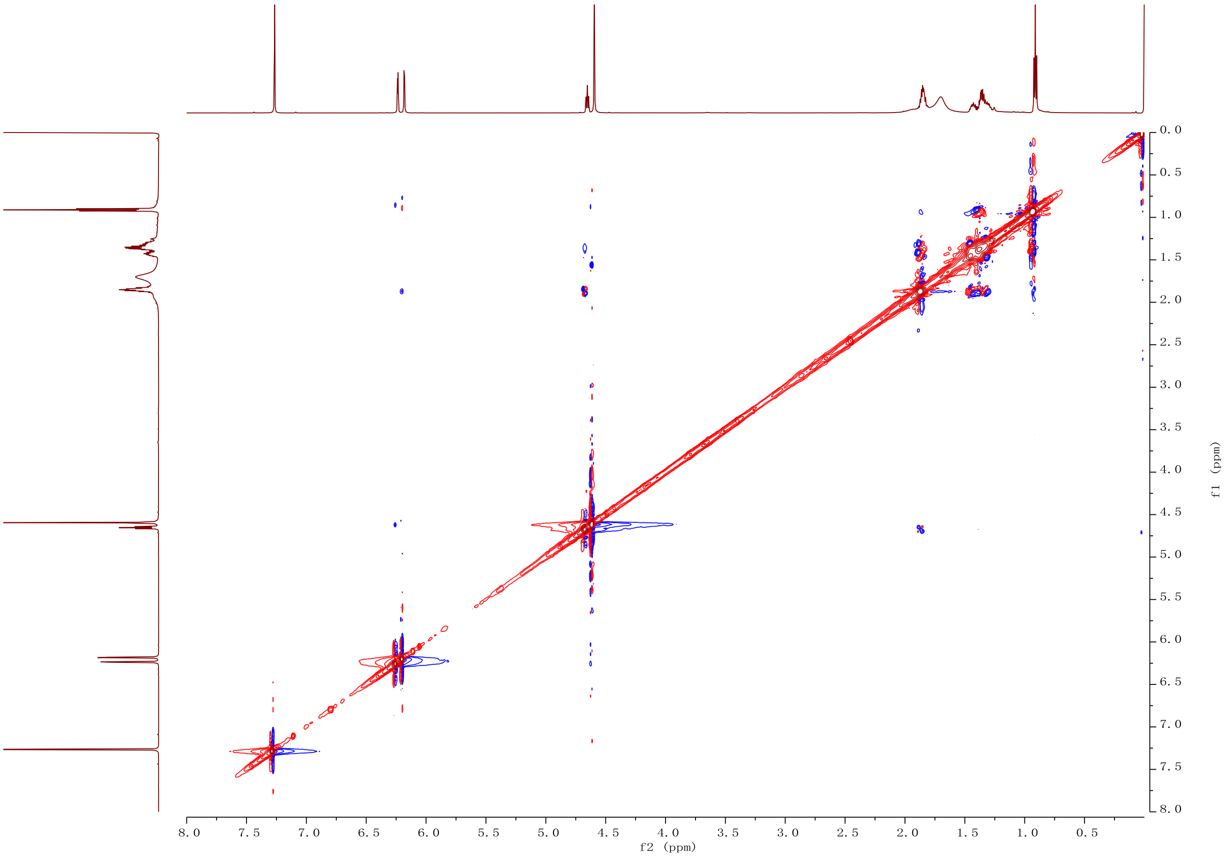

Figure S23. HR-ESI-MS of compound **3**

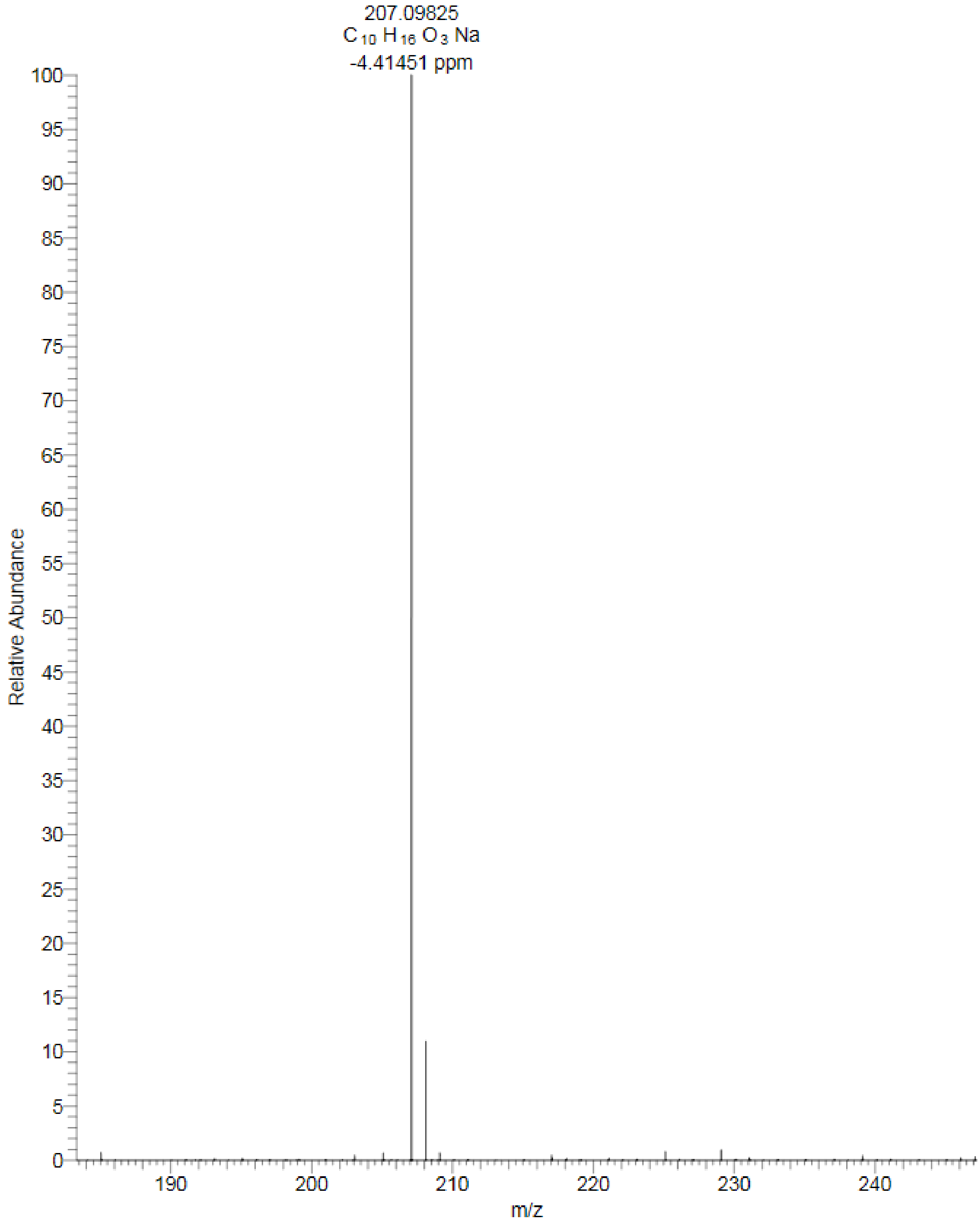

Figure S24. ^{1}H NMR (600 MHz, $CDCl_3$) spectrum of compound **4**

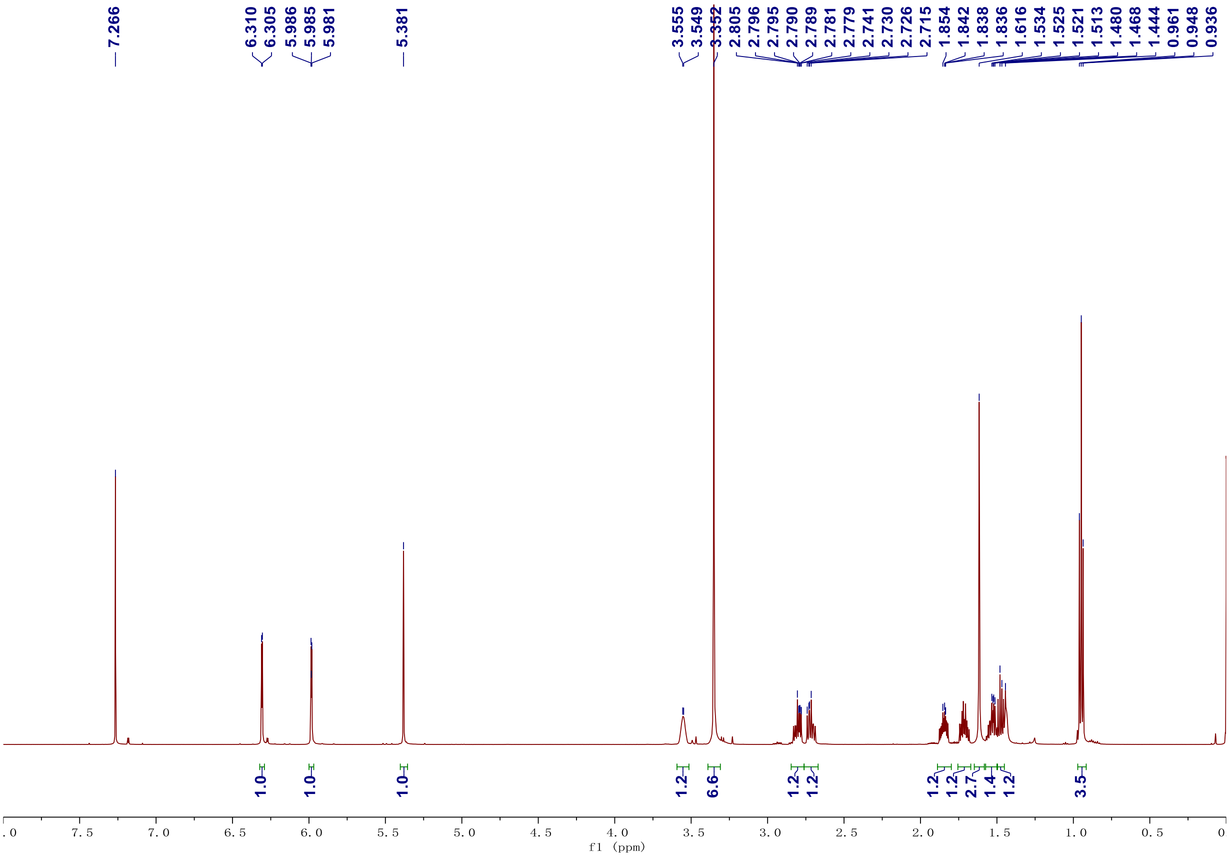

Figure S25. ^{13}C NMR (150 MHz, $CDCl_3$) spectrum of compound **4**

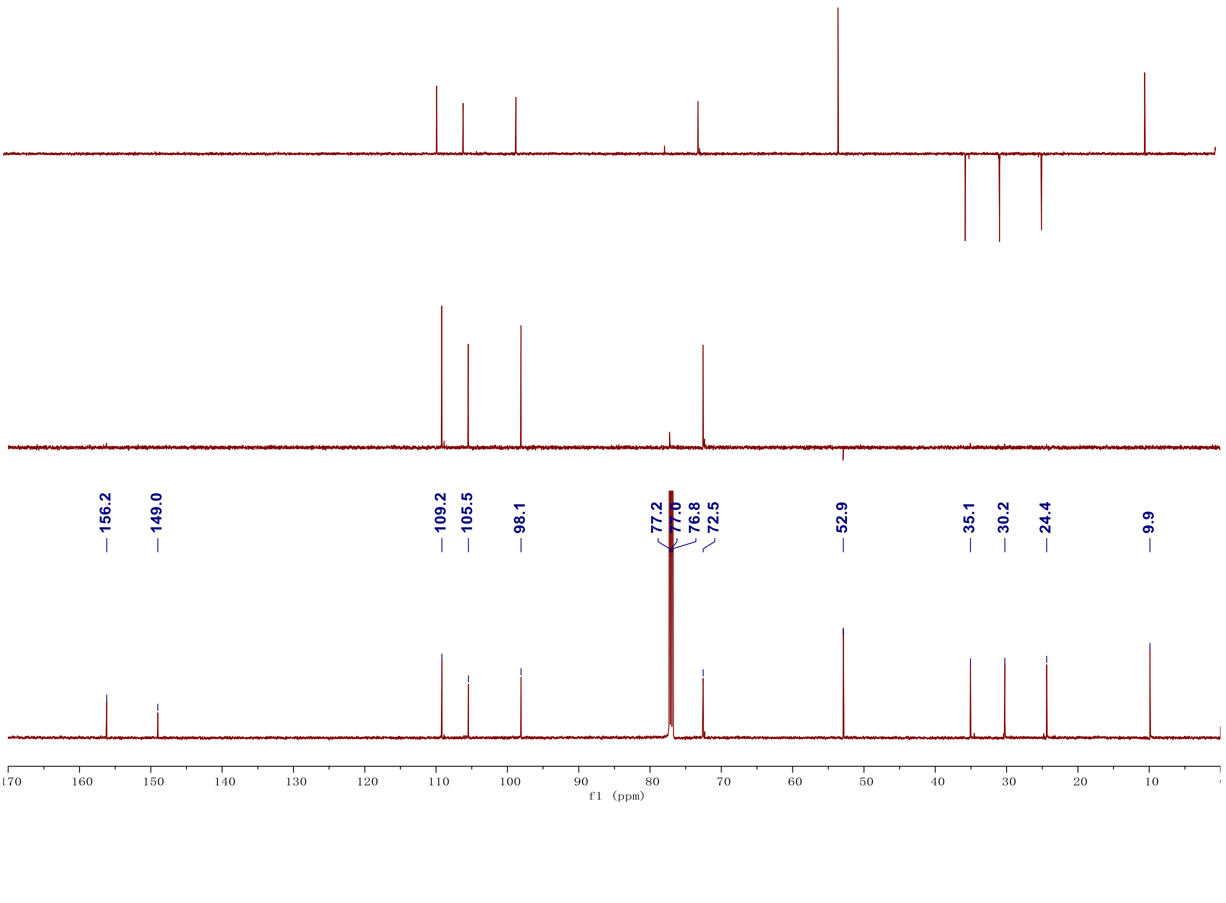

Figure S26. HSQC spectrum of compound **4**

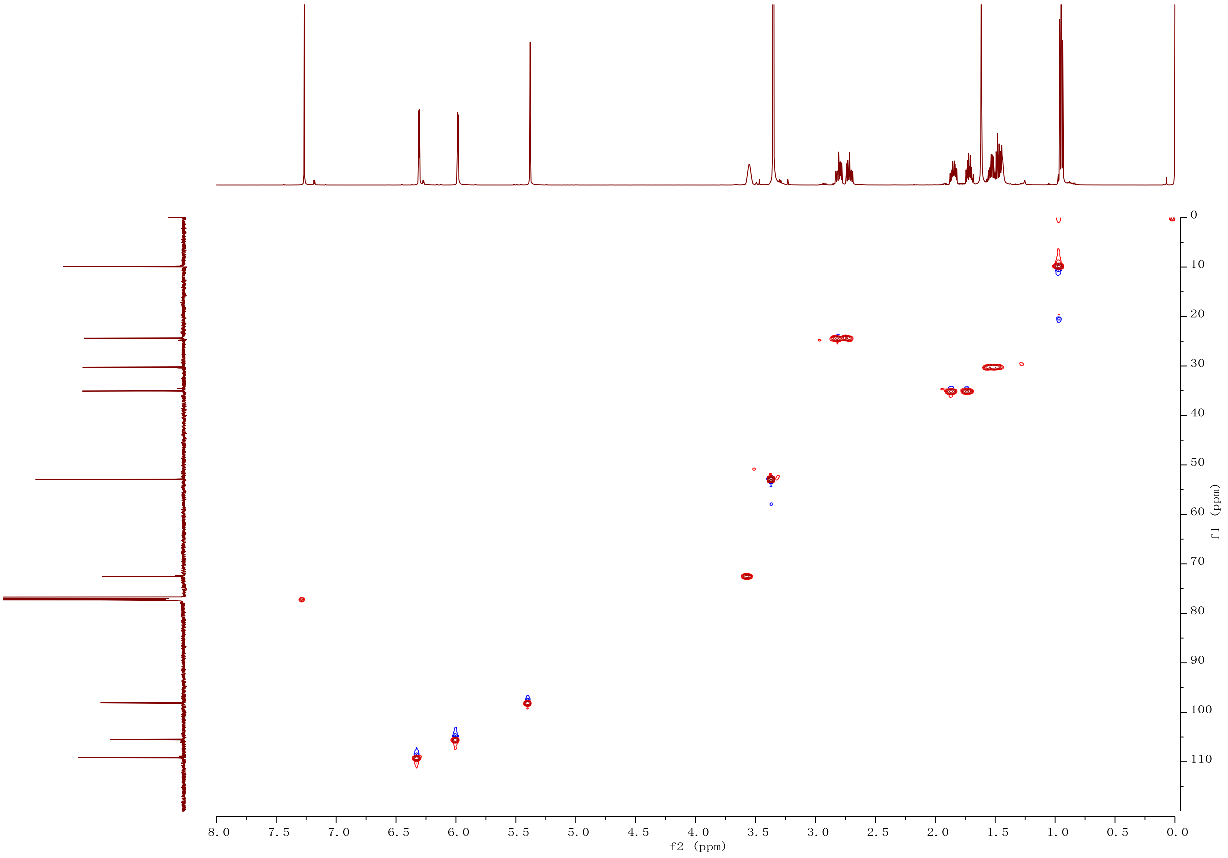

Figure S27. HMBC spectrum of compound **4**

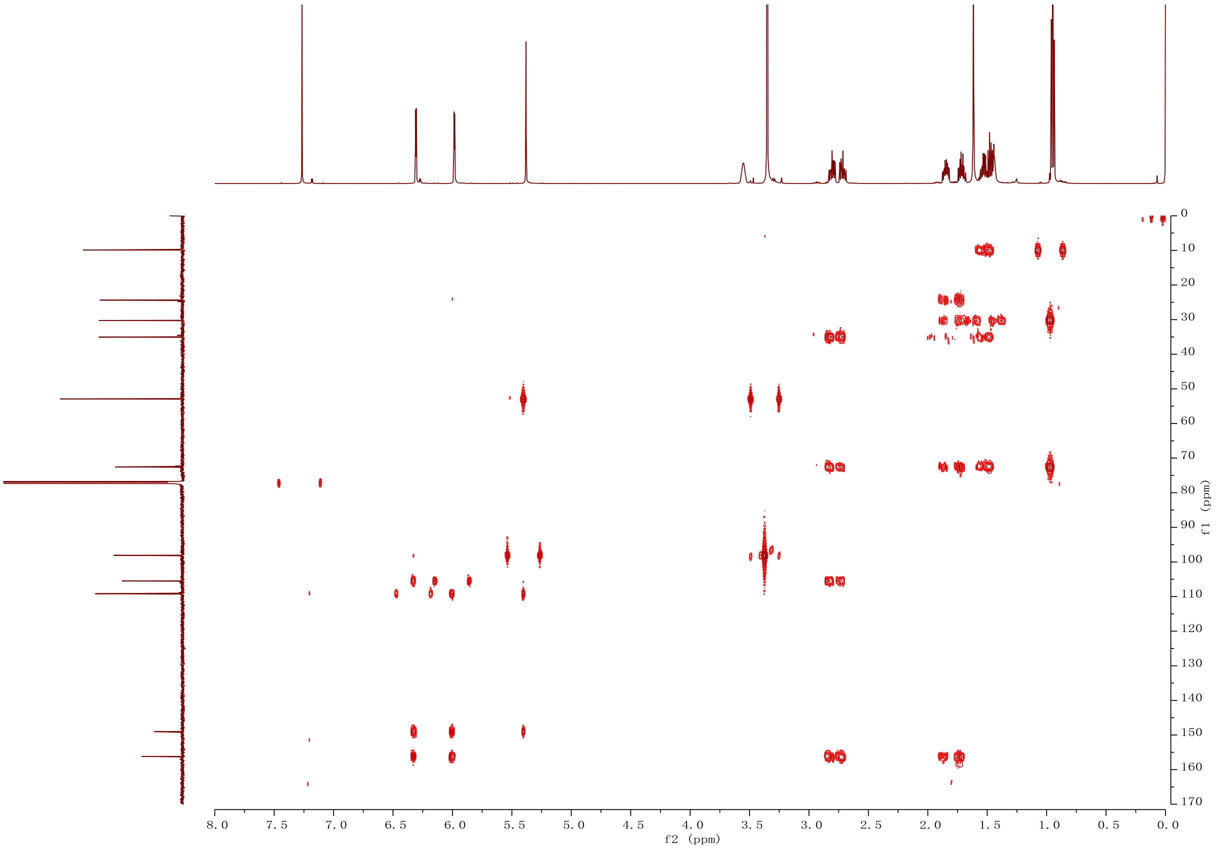

Figure S28. COSY spectrum of compound **4**

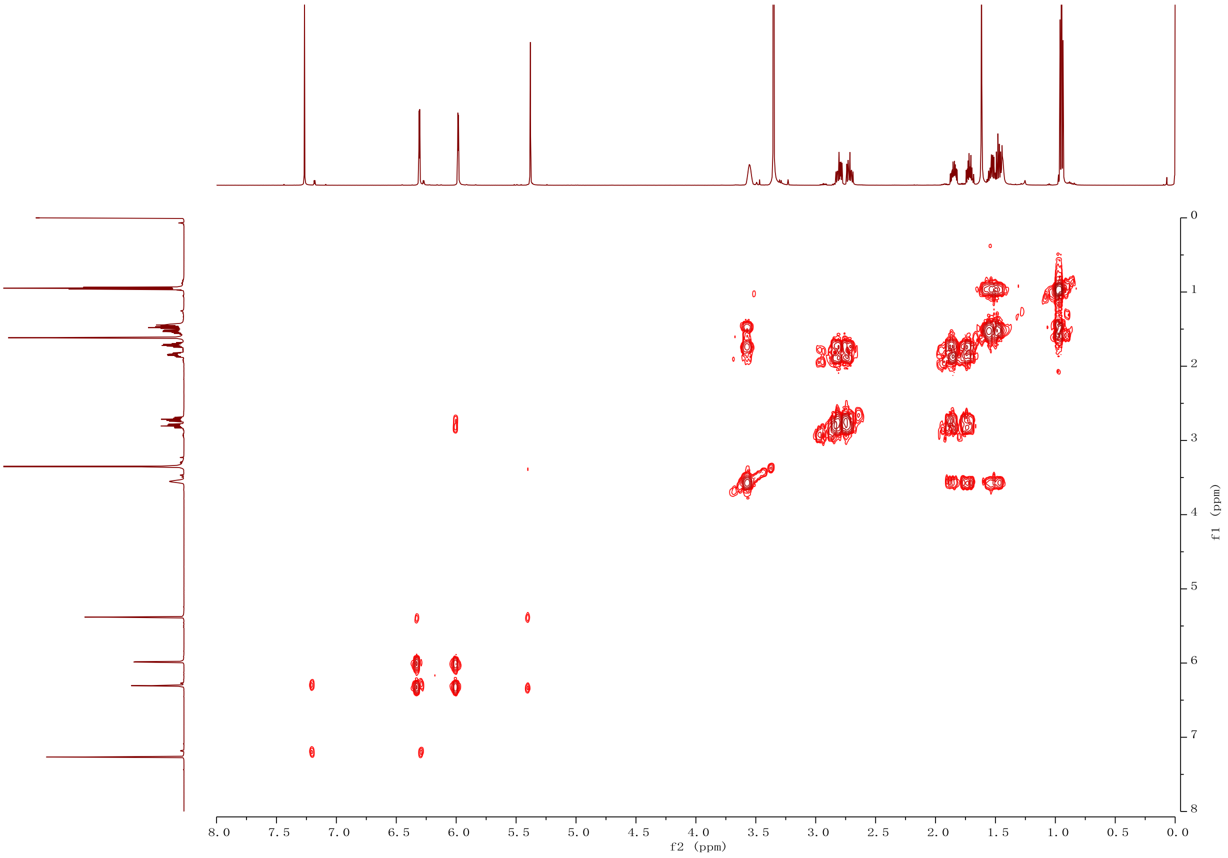

Figure S29. ROESY spectrum of compound **4**

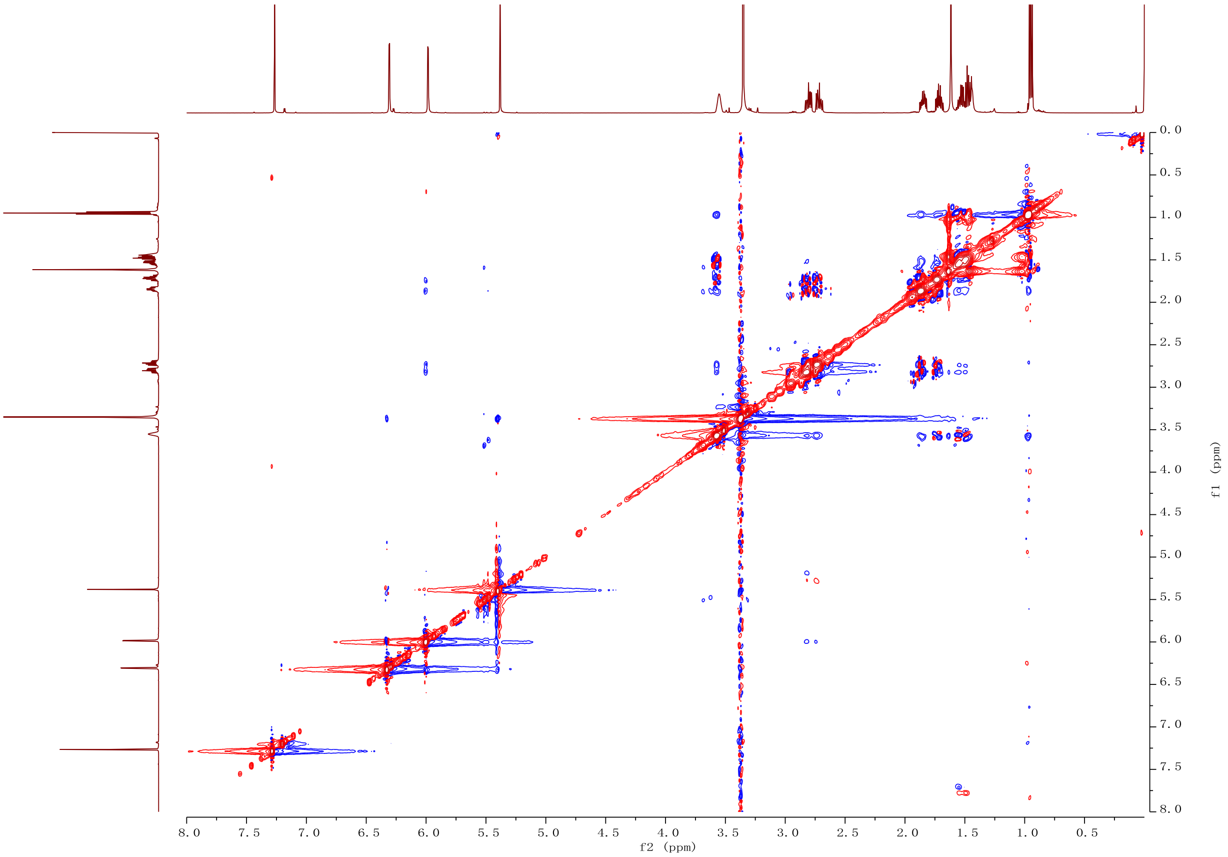

Figure S30. HR-ESI-MS of compound **4**

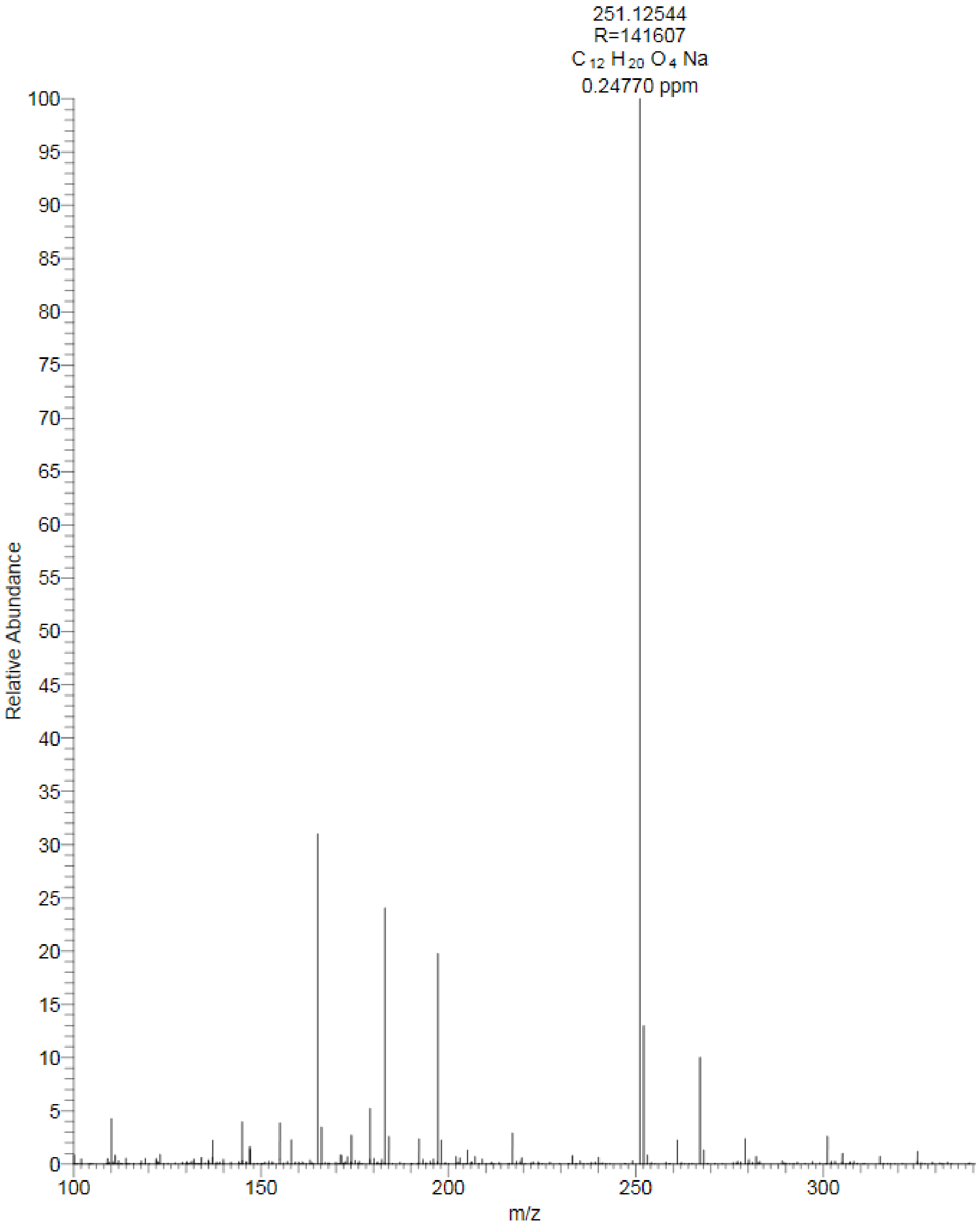

Figure S31. ^{1}H NMR (600 MHz, $CDCl_3$) spectrum of compound **5**

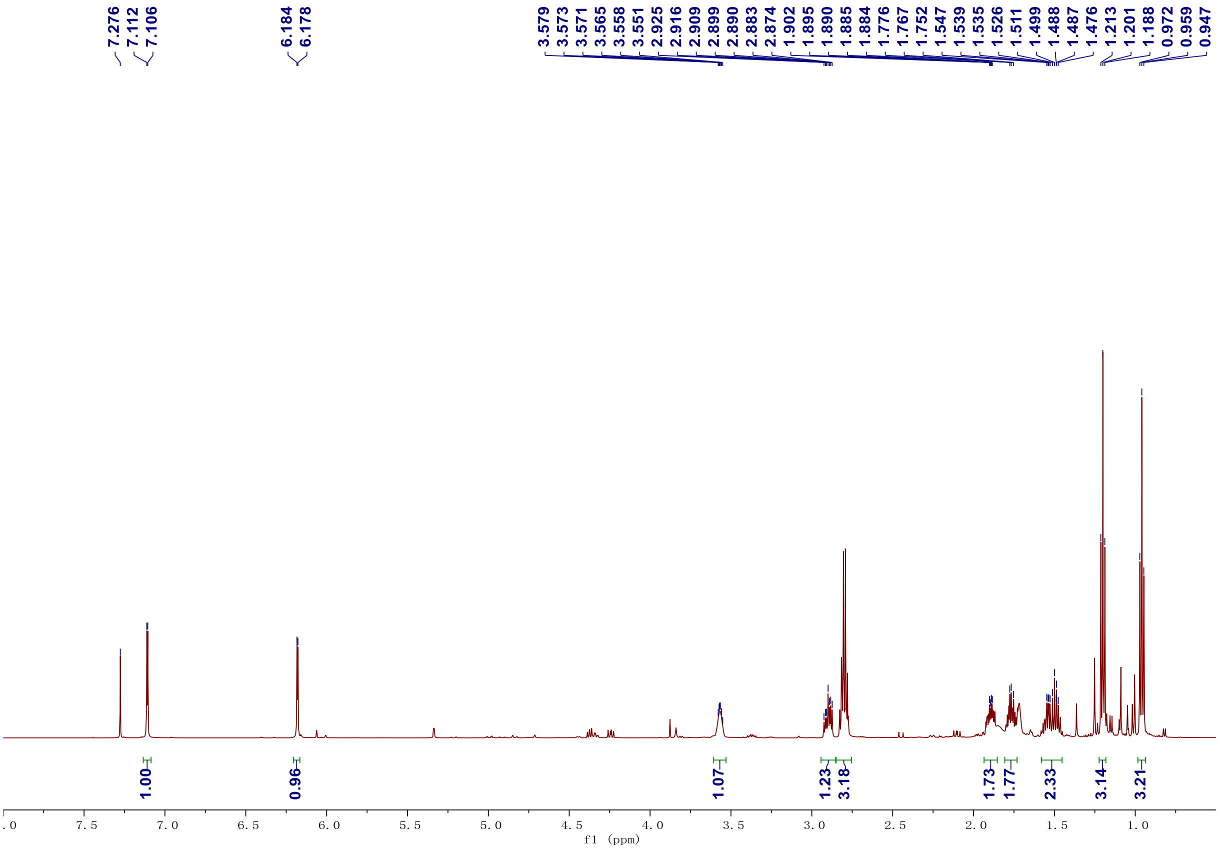

Figure S32. ^{13}C NMR (150 MHz, $CDCl_3$) spectrum of compound **5**

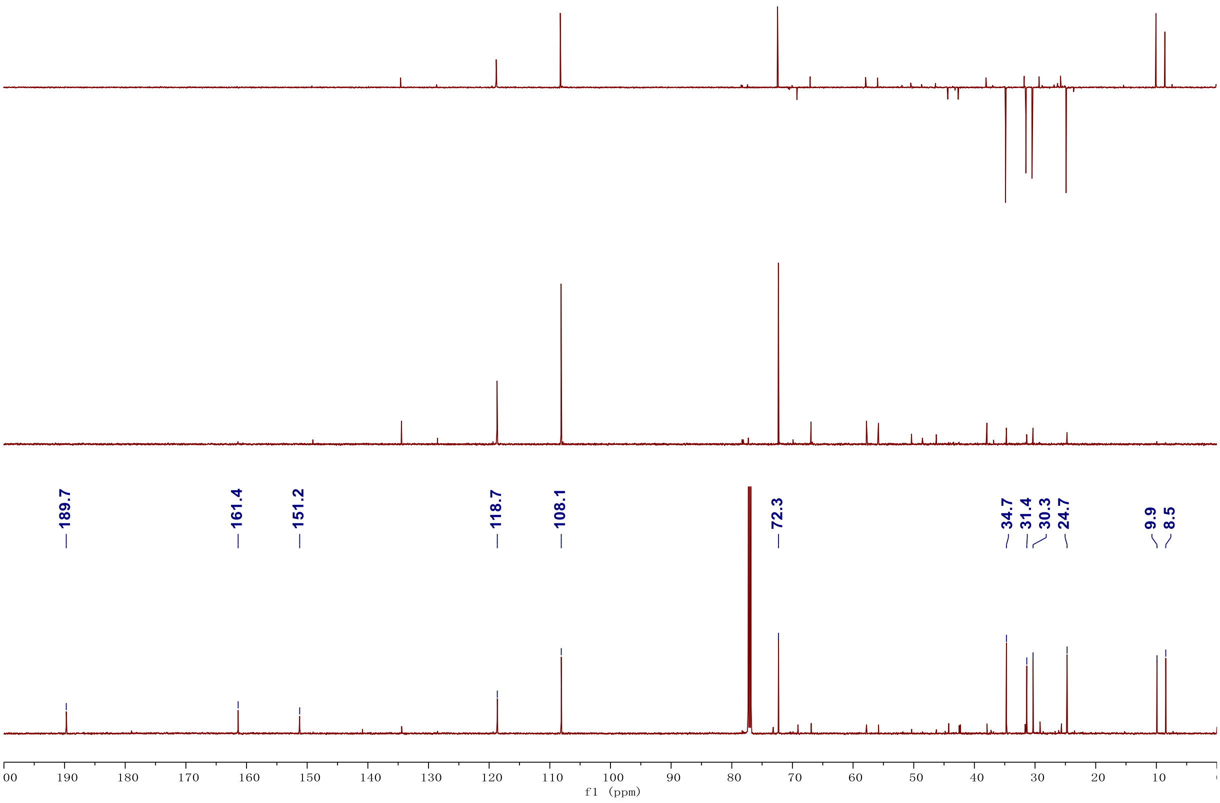

Figure S33. HSQC spectrum of compound **5**

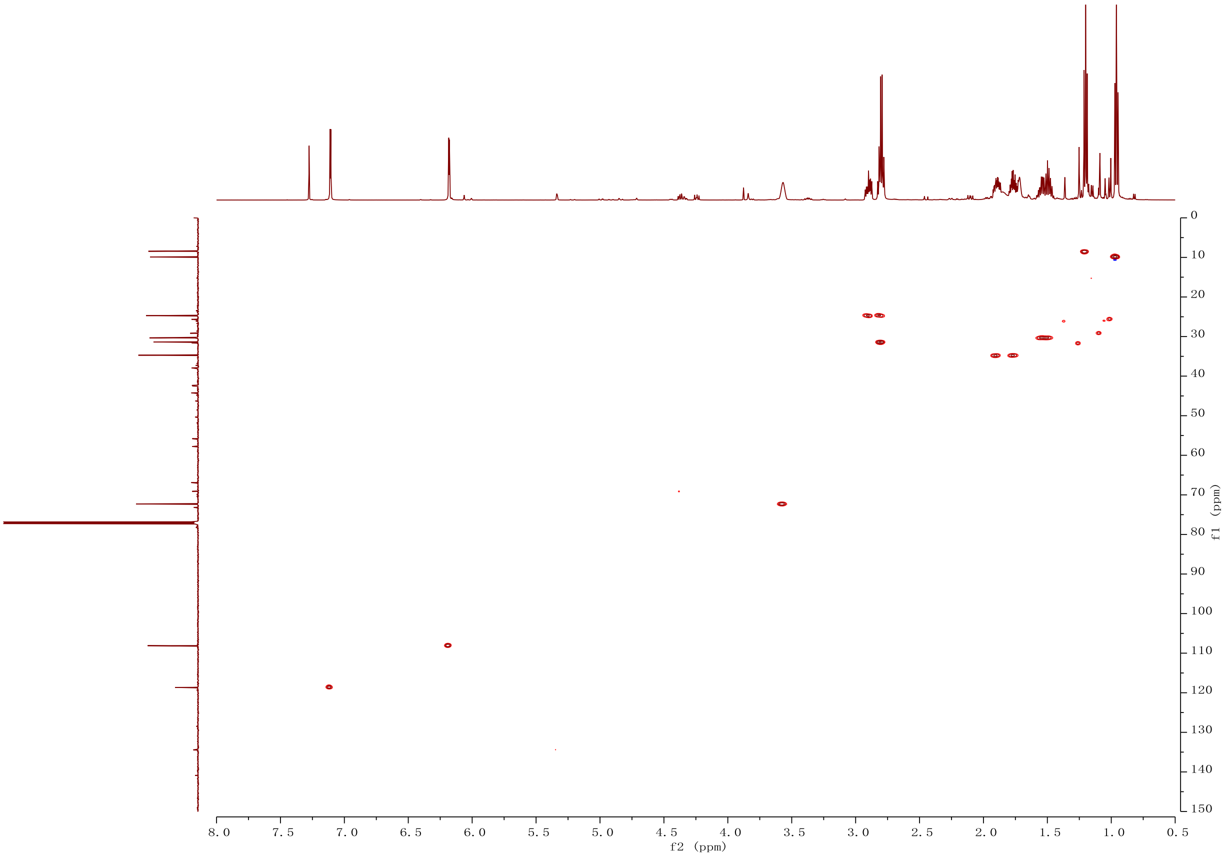

Figure S34. HMBC spectrum of compound **5**

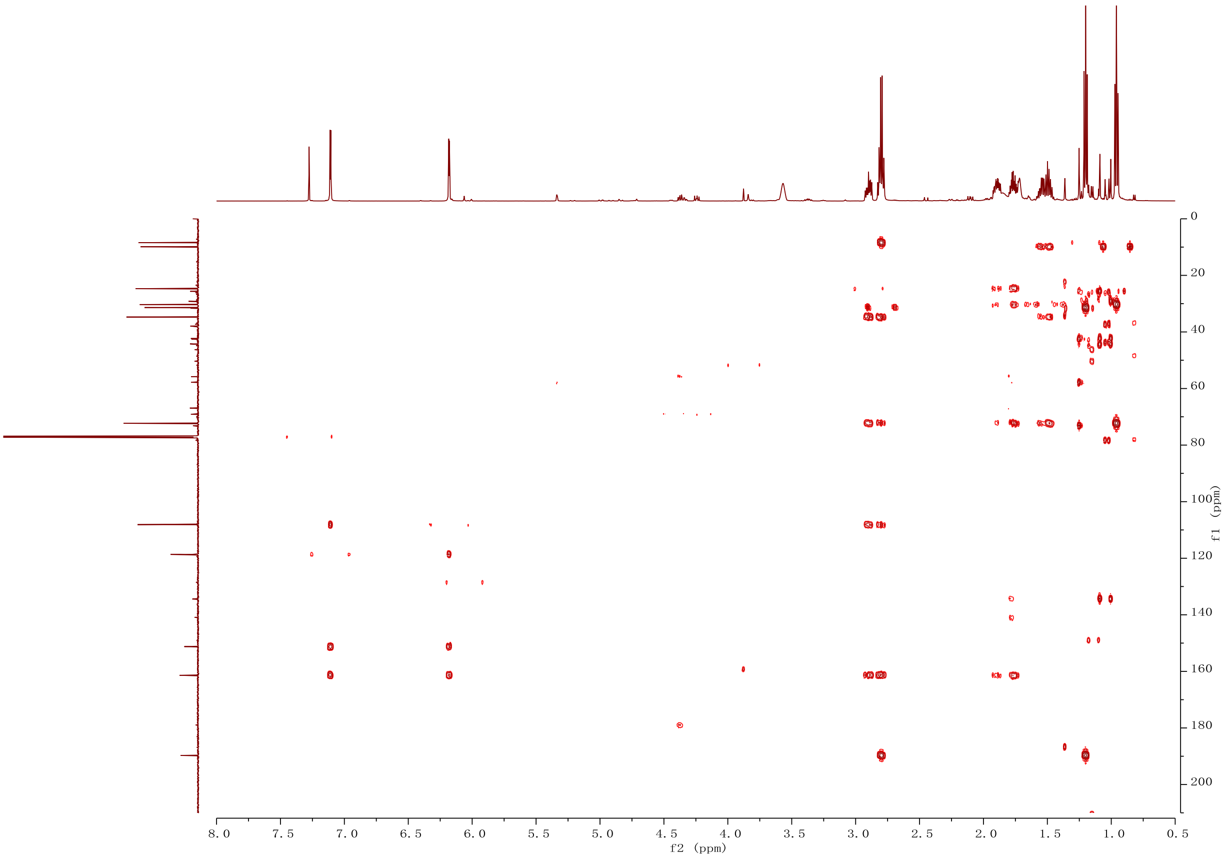

Figure S35. COSY spectrum of compound **5**

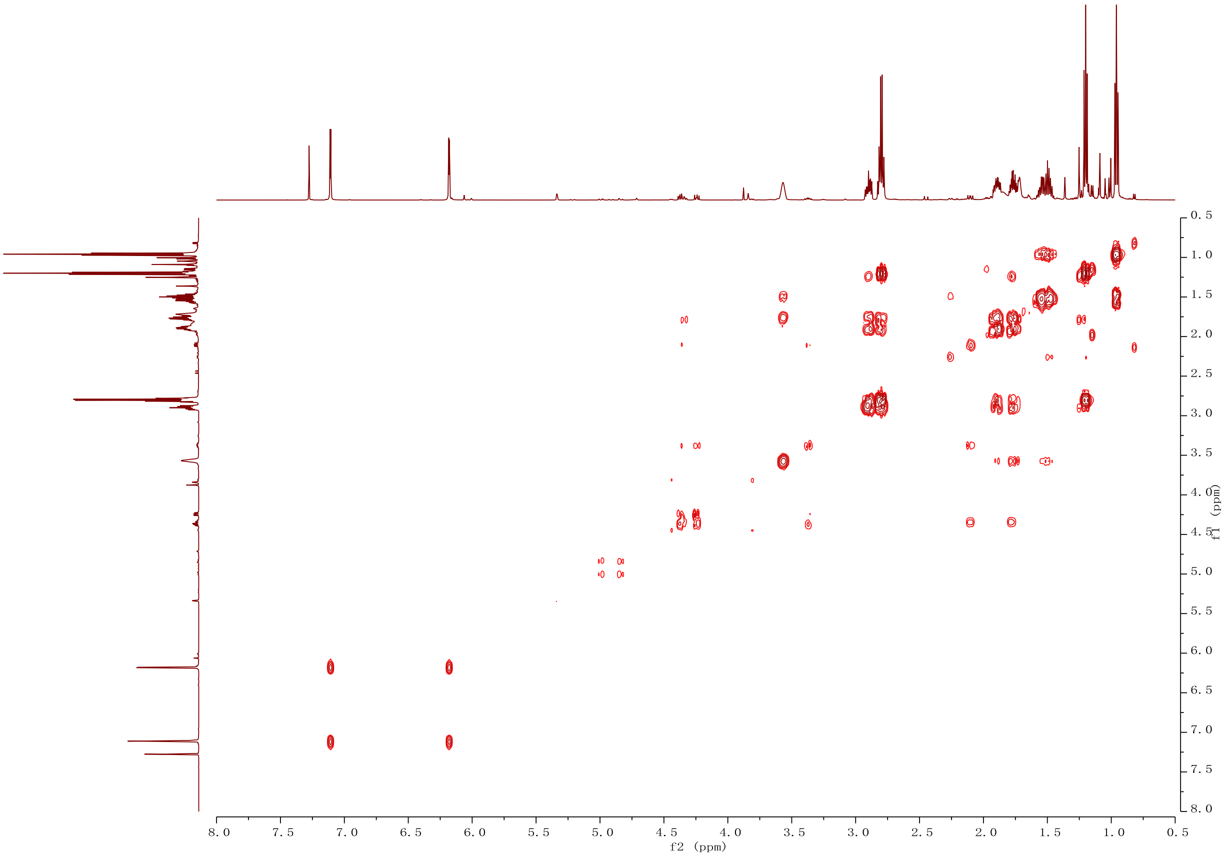

Figure S36. ROESY spectrum of compound **5**

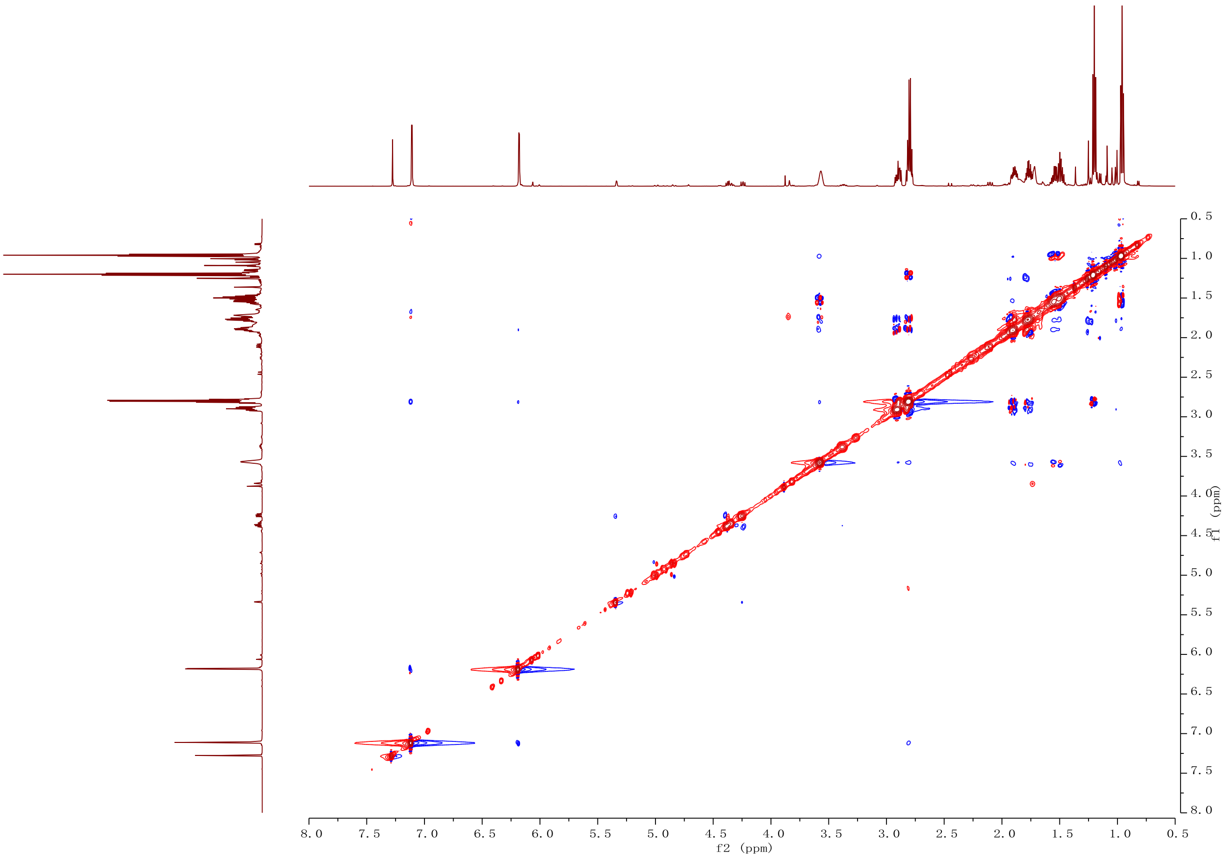

Figure S37. HR-ESI-MS of compound **5**

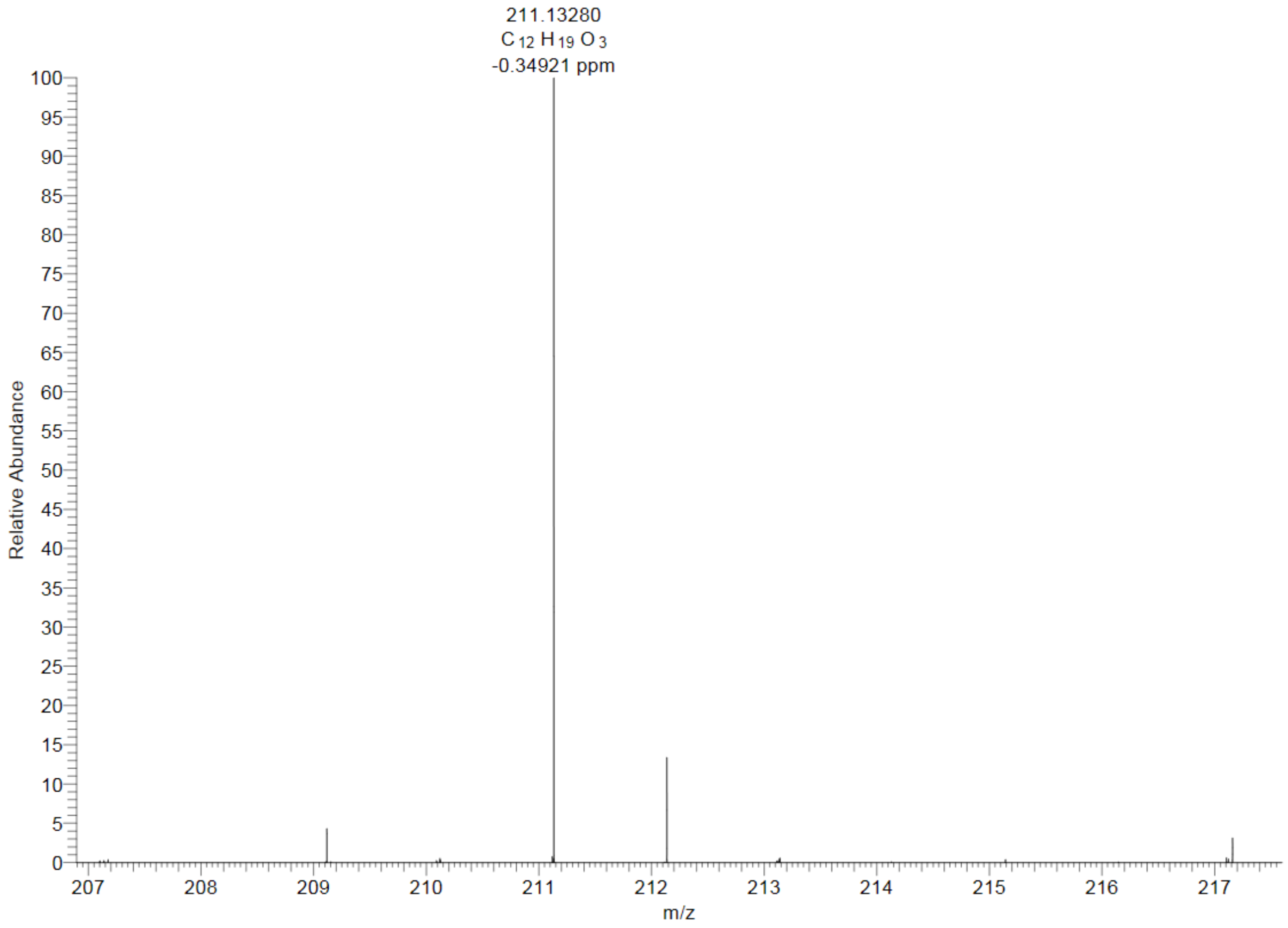

Figure S38. ^{1}H NMR (600 MHz, $CDCl_3$) spectrum of compound **6**

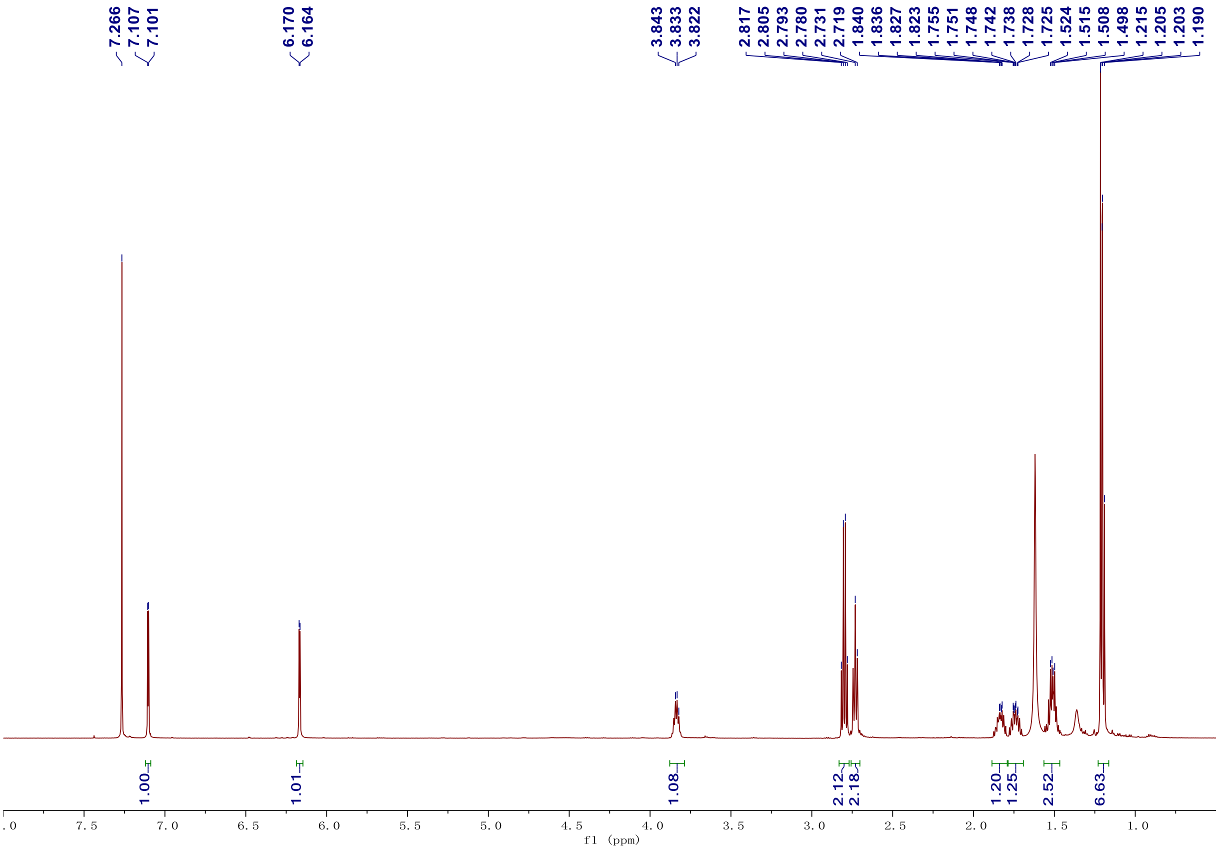

Figure S39. ^{13}C NMR (150 MHz, $CDCl_3$) spectrum of compound **6**

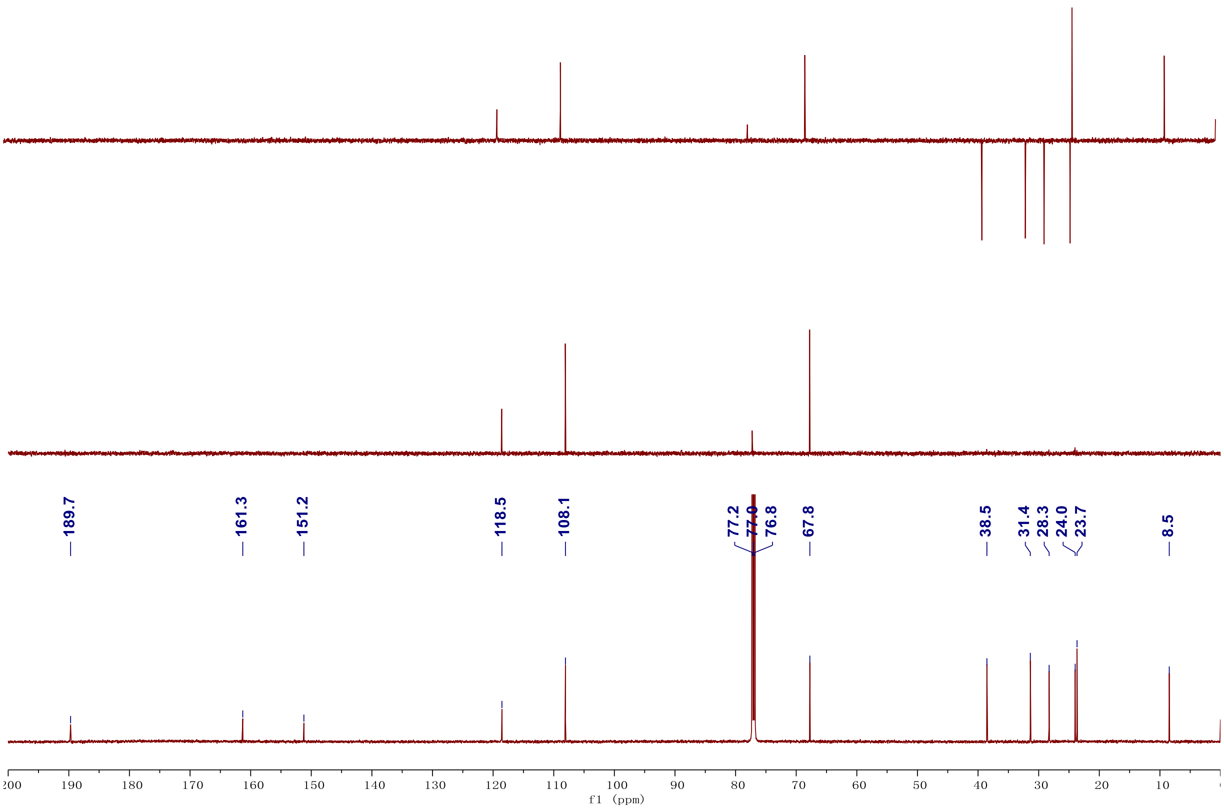

Figure S40. HSQC spectrum of compound **6**

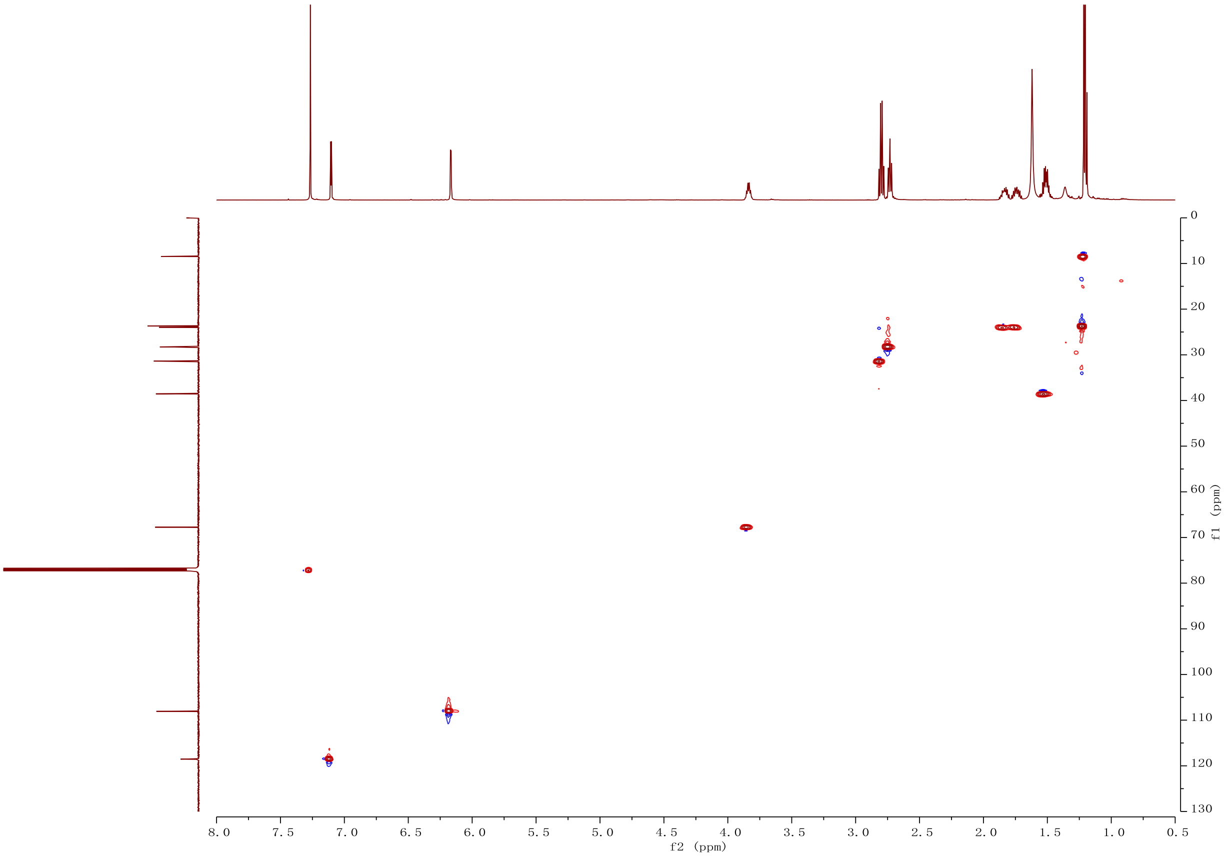

Figure S41. HMBC spectrum of compound **6**

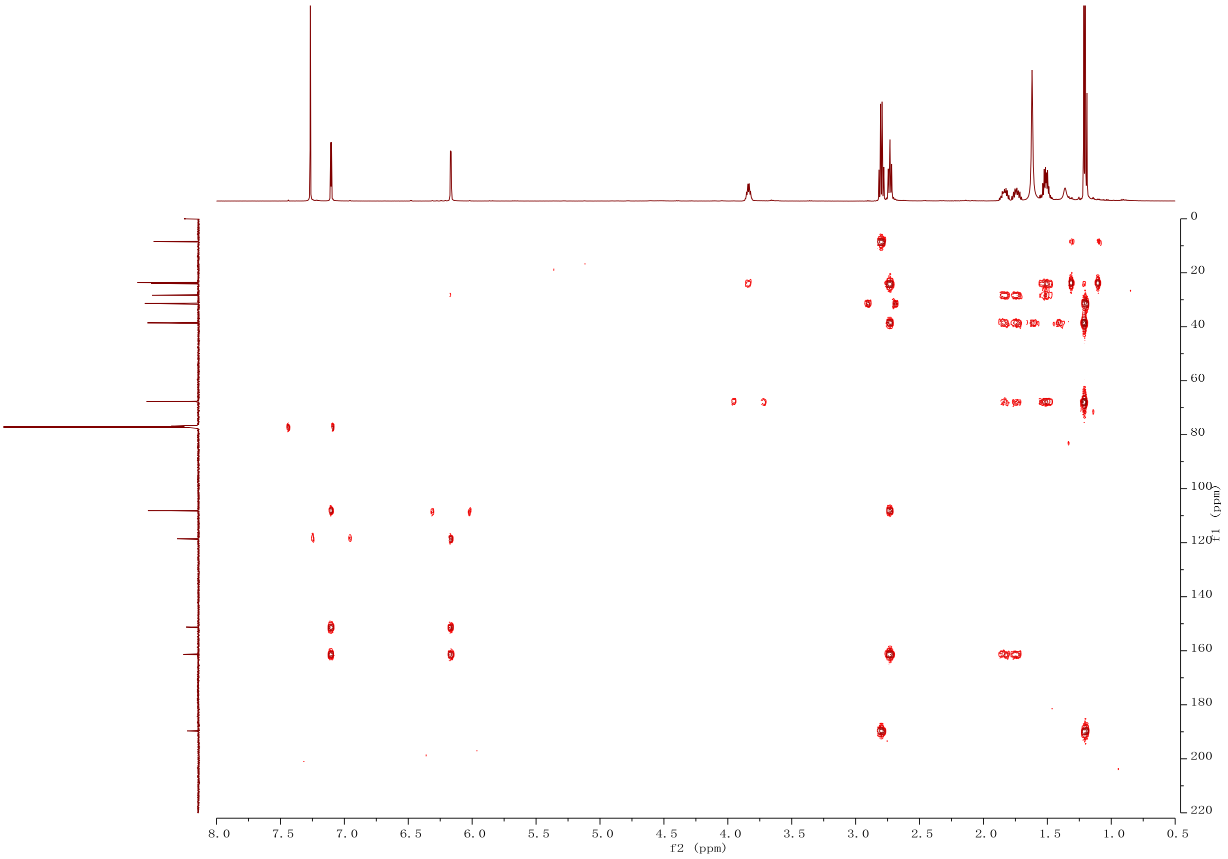

Figure S42. COSY spectrum of compound **6**

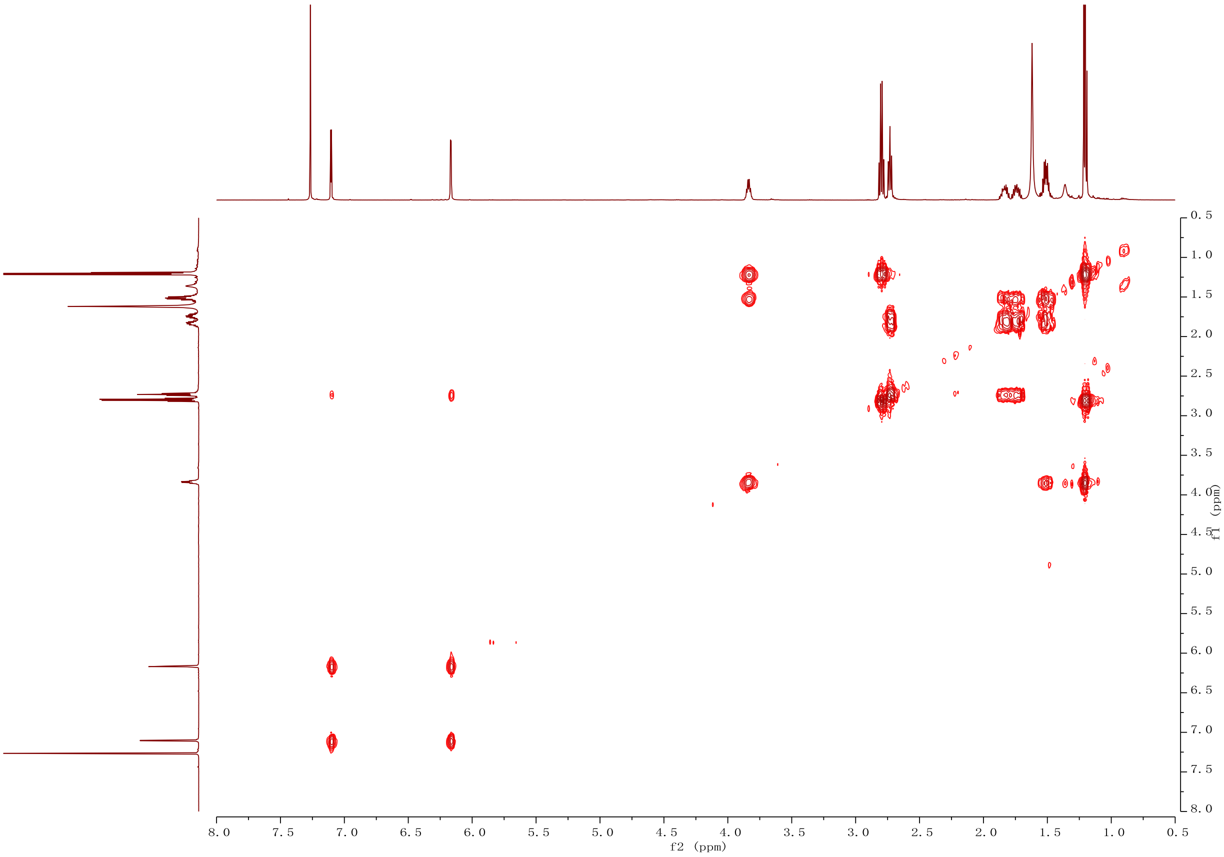

Figure S43. ROESY spectrum of compound **6**

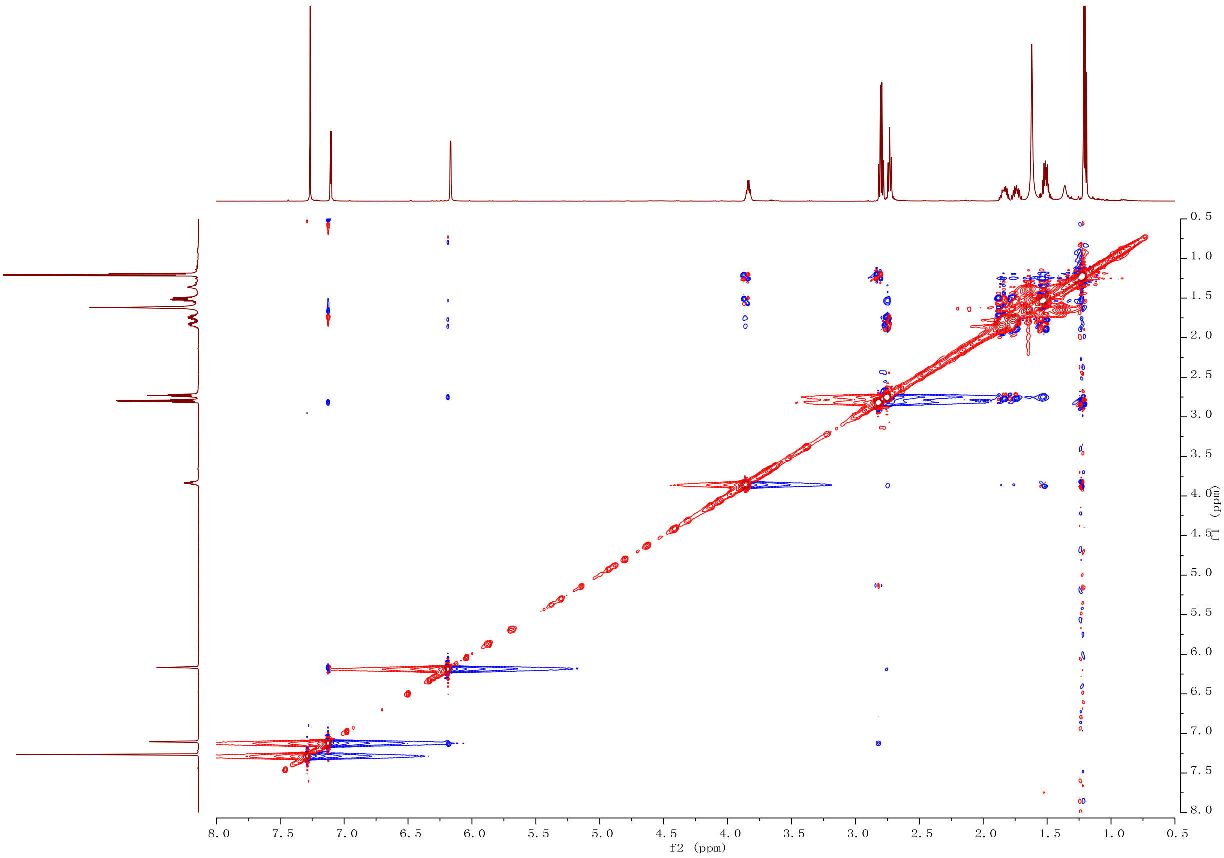

Figure S44. HR-ESI-MS of compound **6**

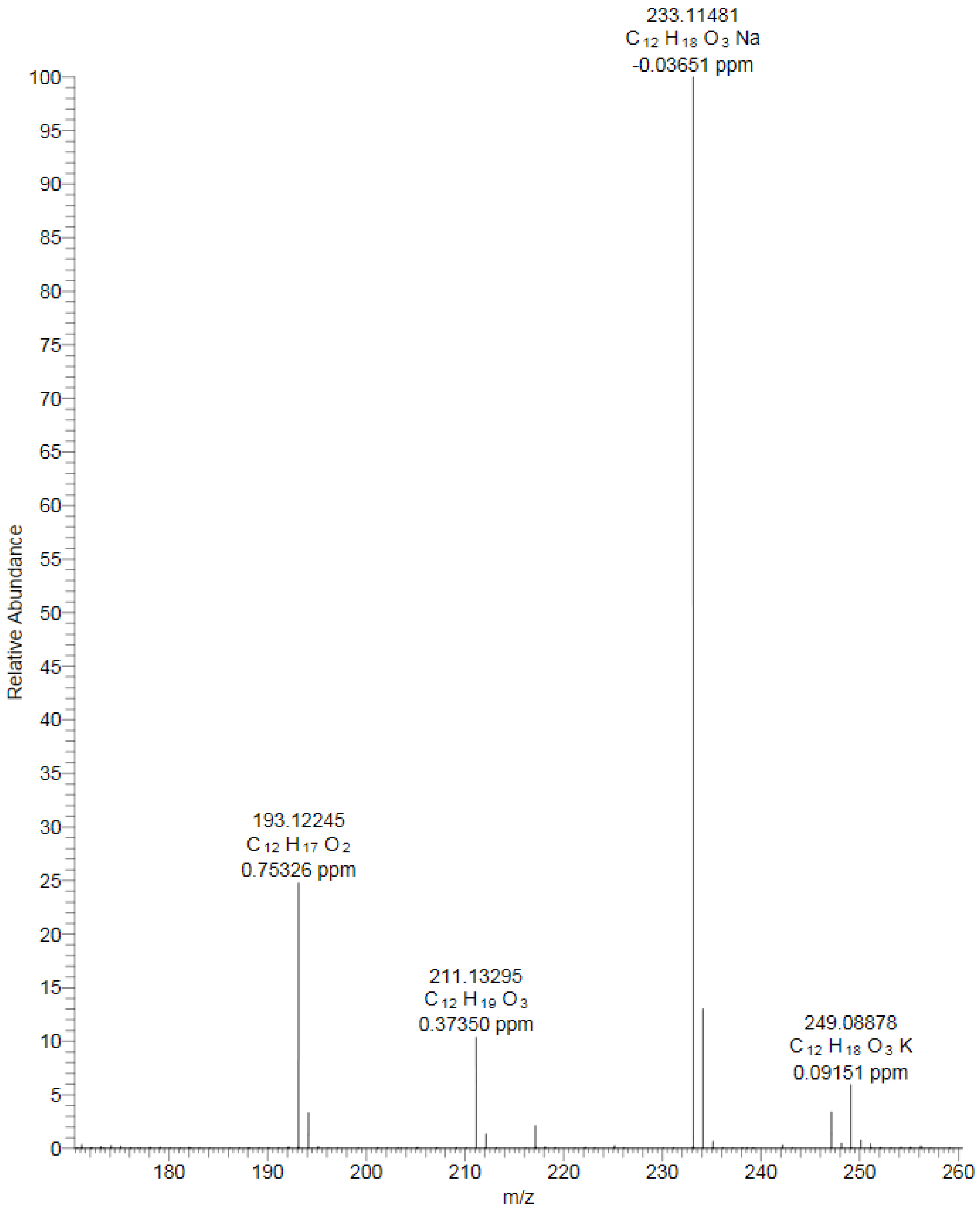

Figure S45. ^{1}H NMR (600 MHz, $CDCl_3$) spectrum of compound **7**

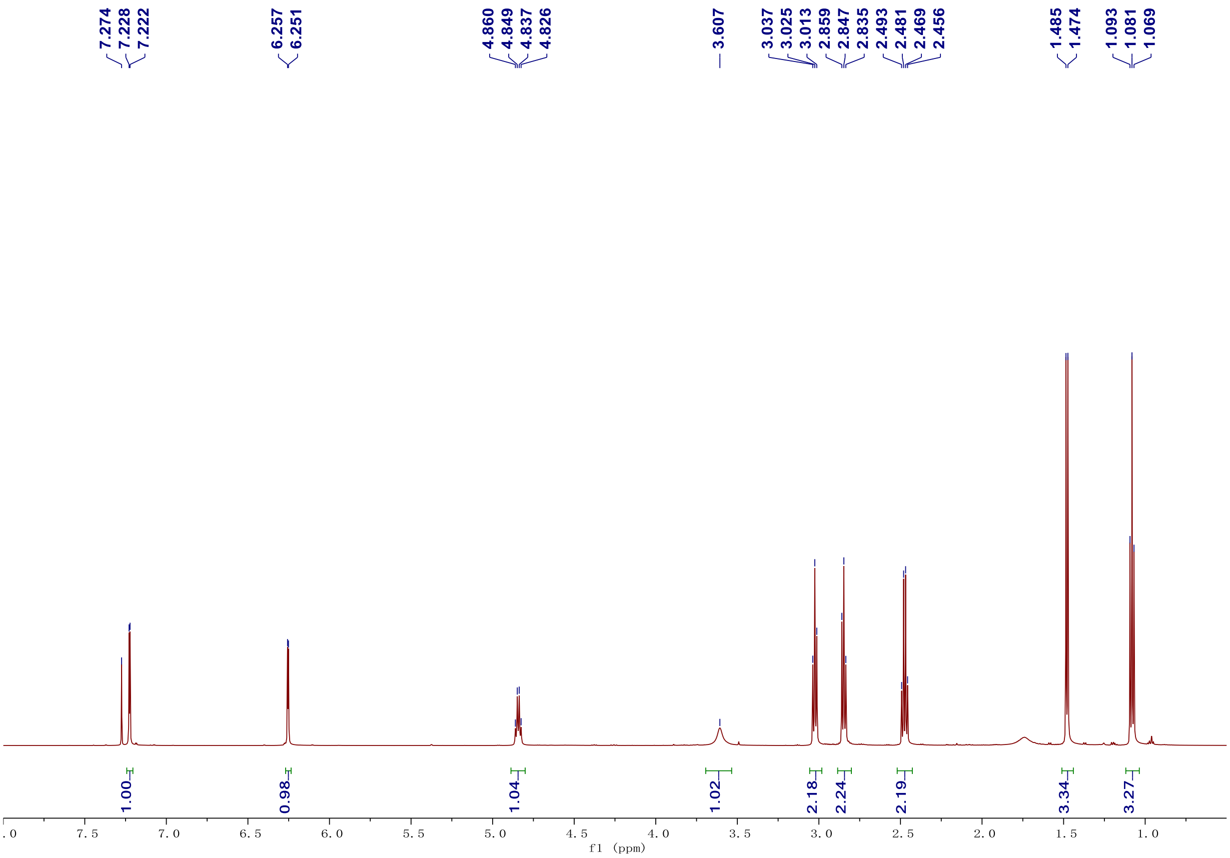

Figure S46. ^{13}C NMR (150 MHz, $CDCl_3$) spectrum of compound **7**

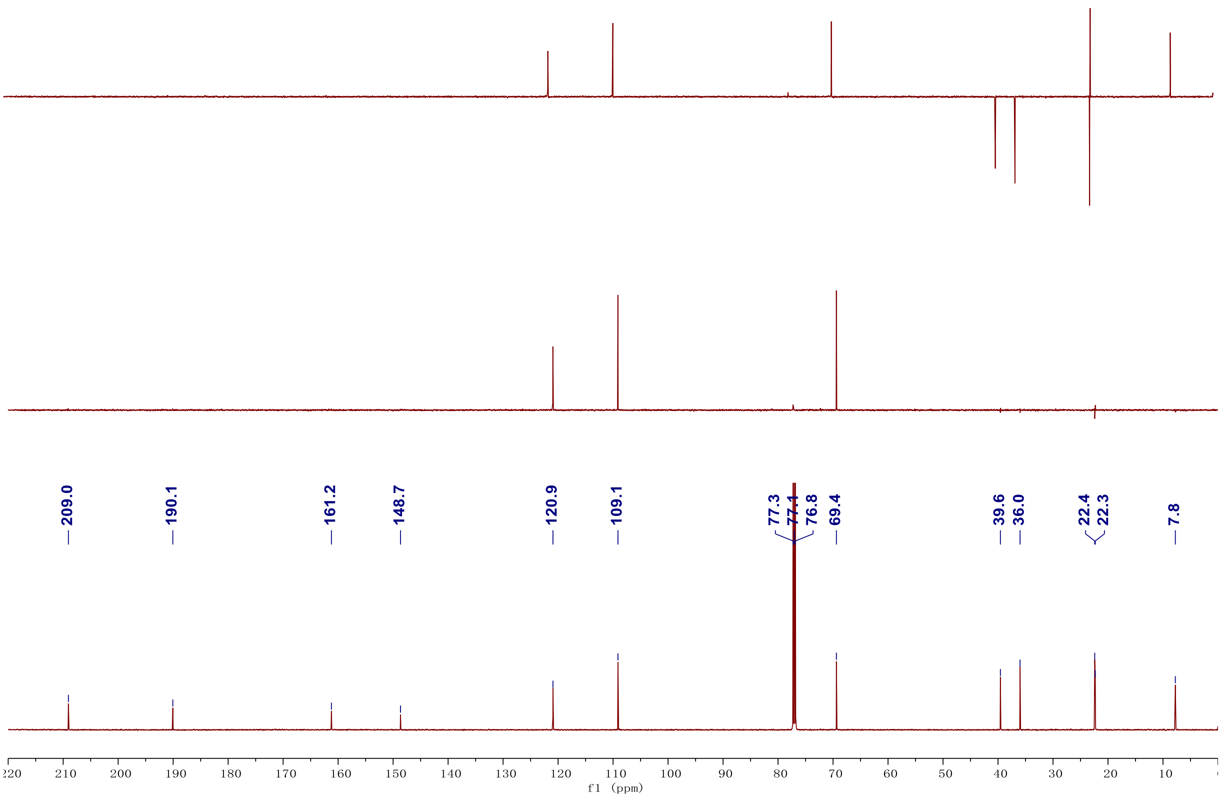

Figure S47. HSQC spectrum of compound **7**

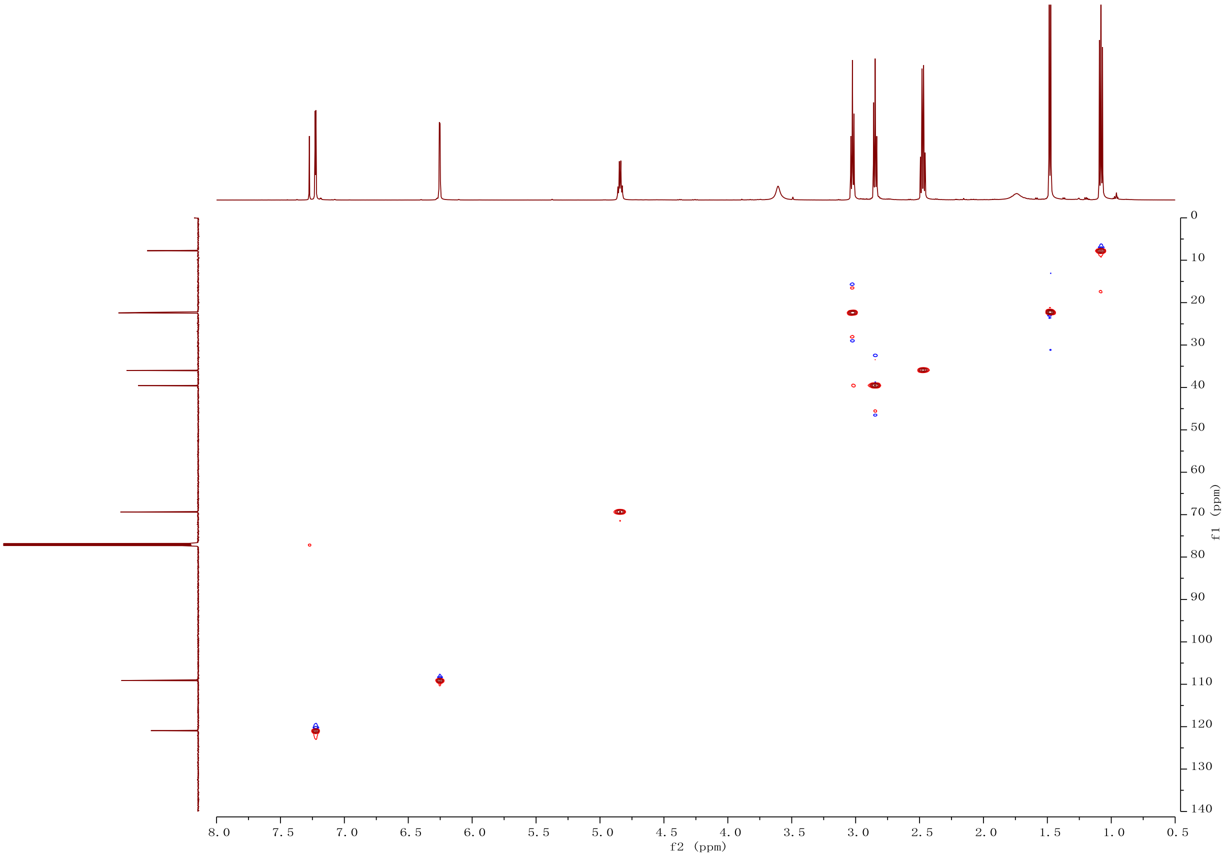

Figure S48. HMBC spectrum of compound **7**

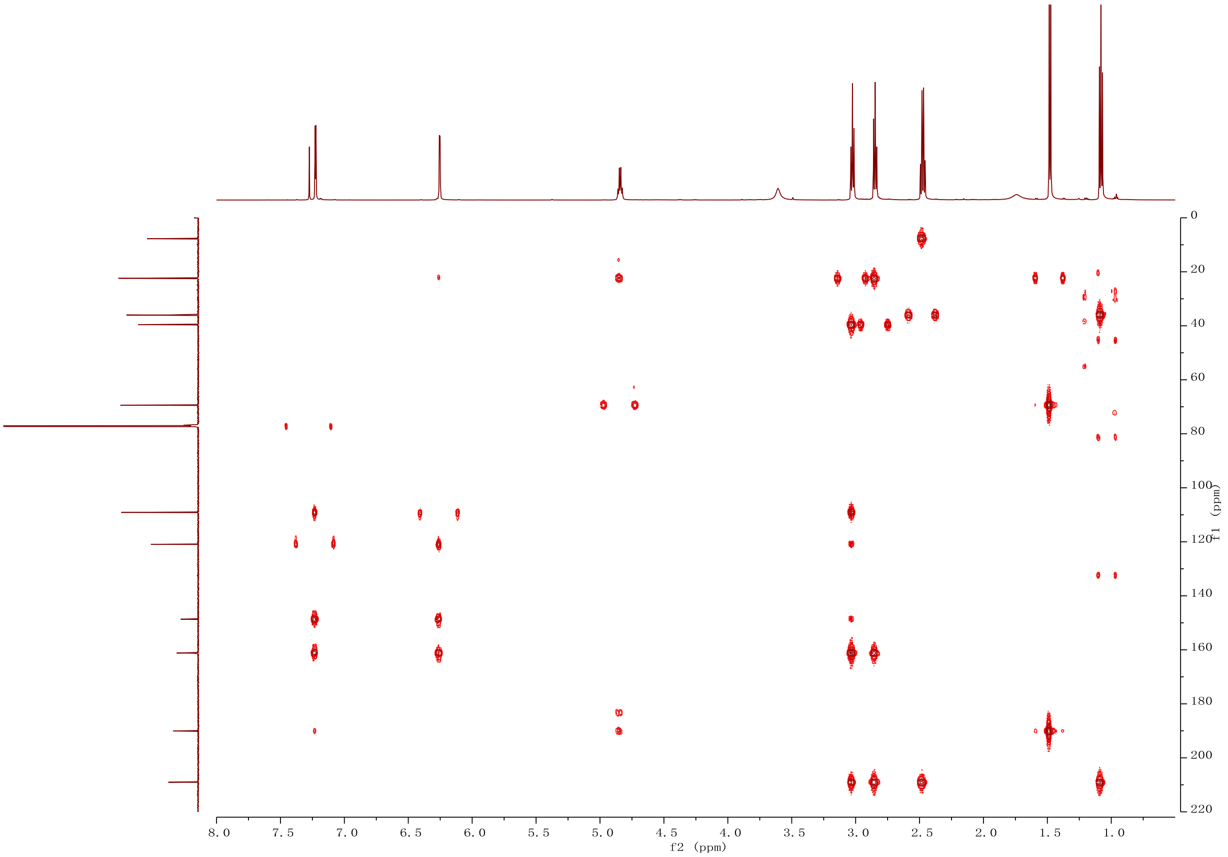

Figure S49. COSY spectrum of compound **7**

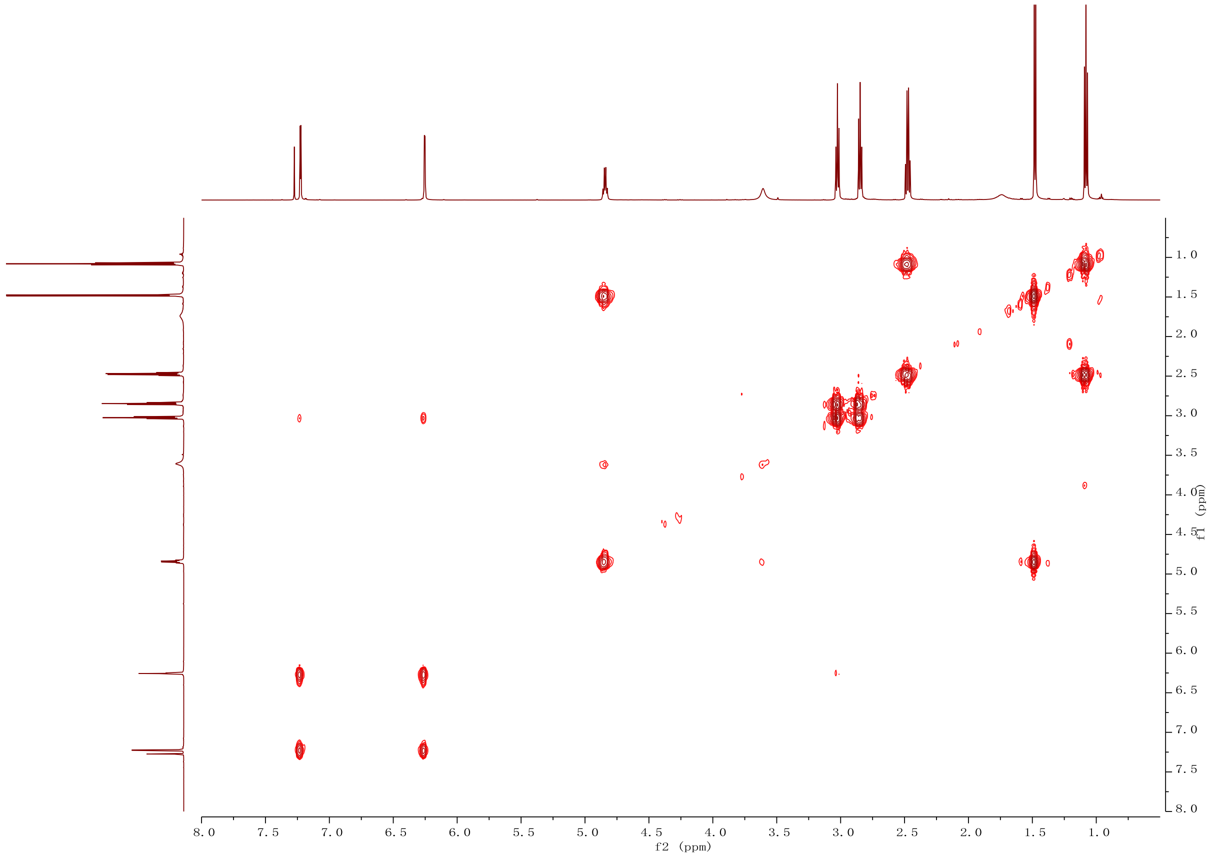

Figure S50. ROESY spectrum of compound **7**

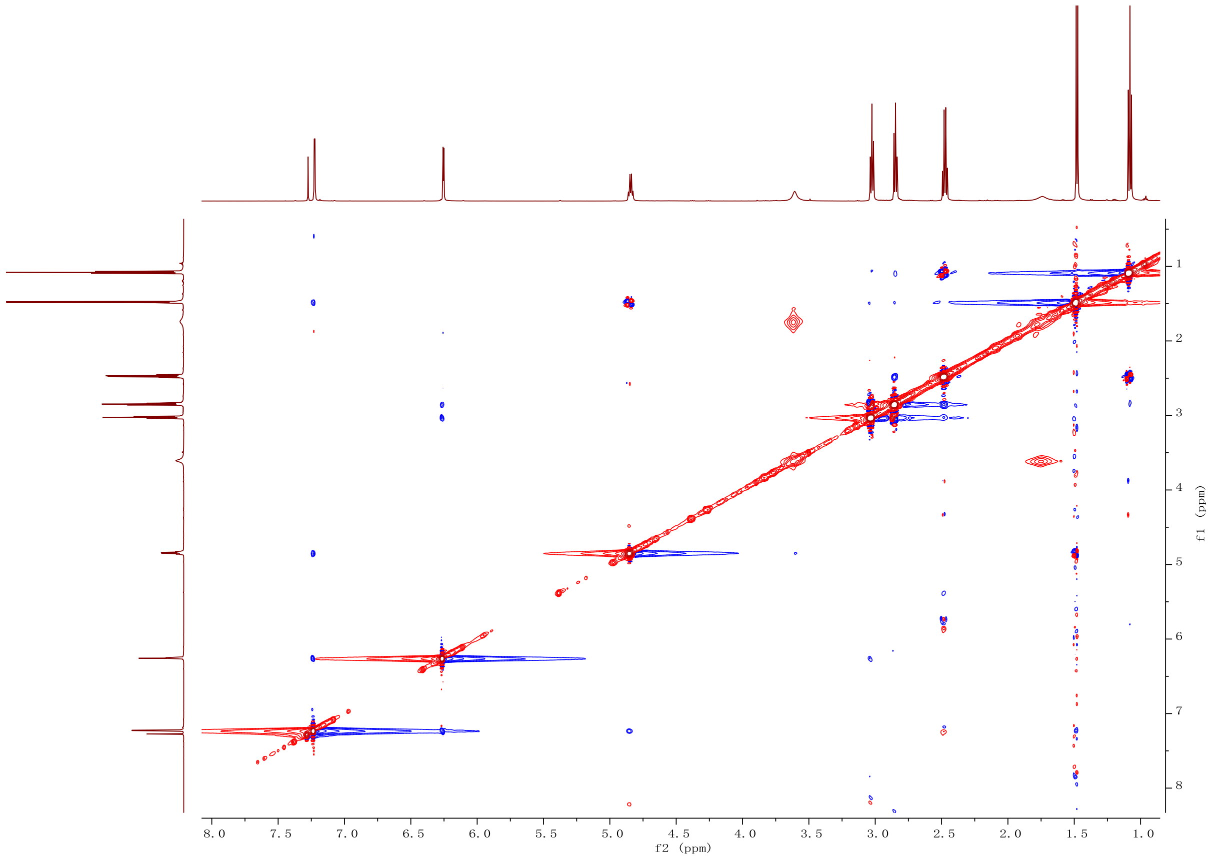

Figure S51. HR-ESI-MS of compound **7**

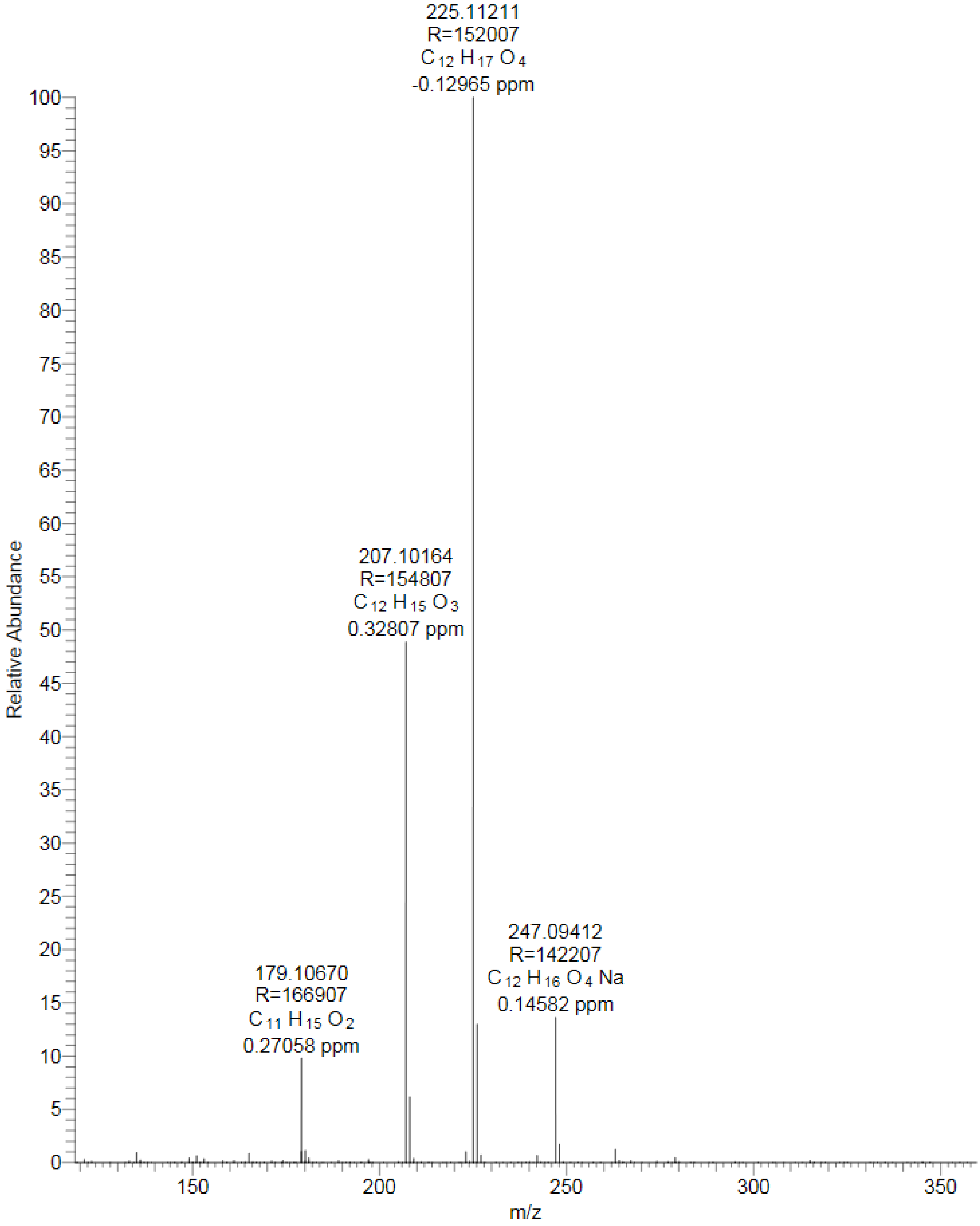

Figure S52. ^{1}H NMR (600 MHz, methanol-d_4) spectrum of compound **8**

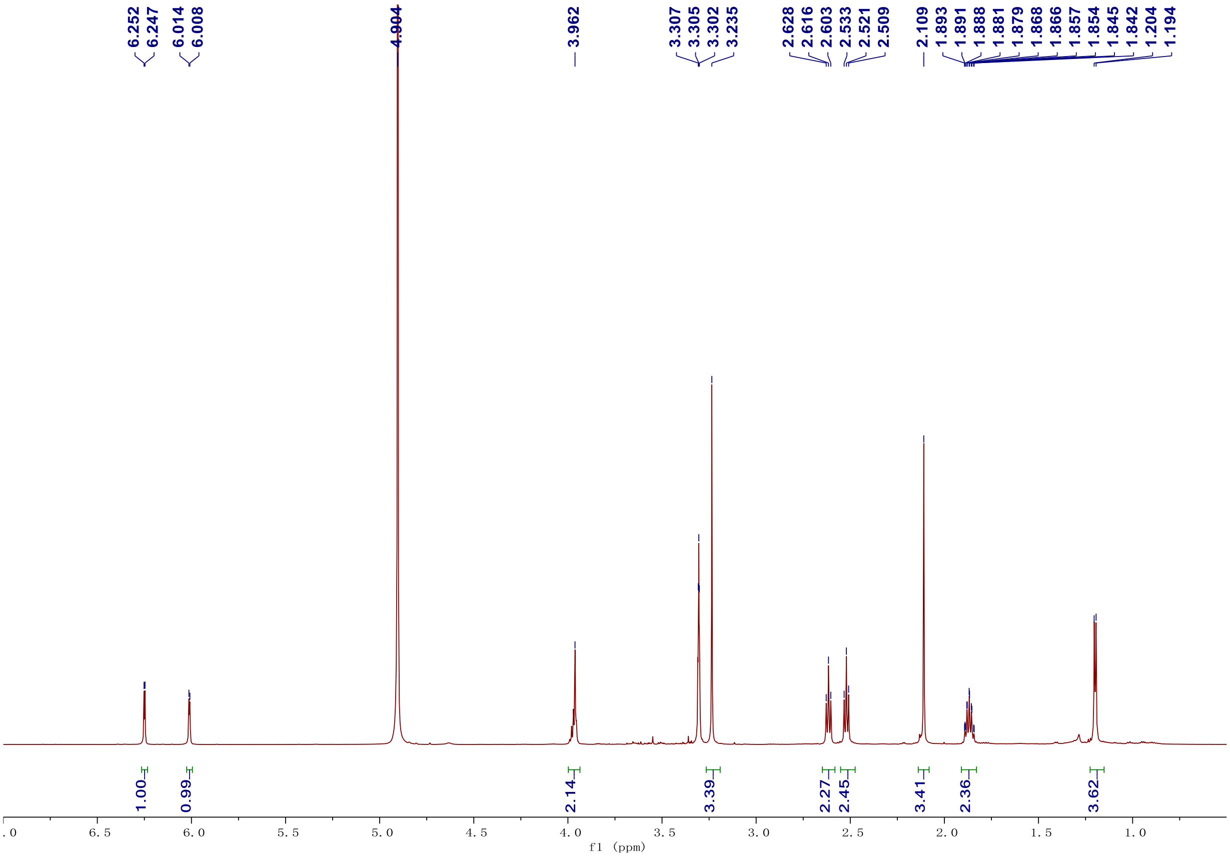

Figure S53. ^{13}C NMR (150 MHz, methanol-d_4) spectrum of compound **8**

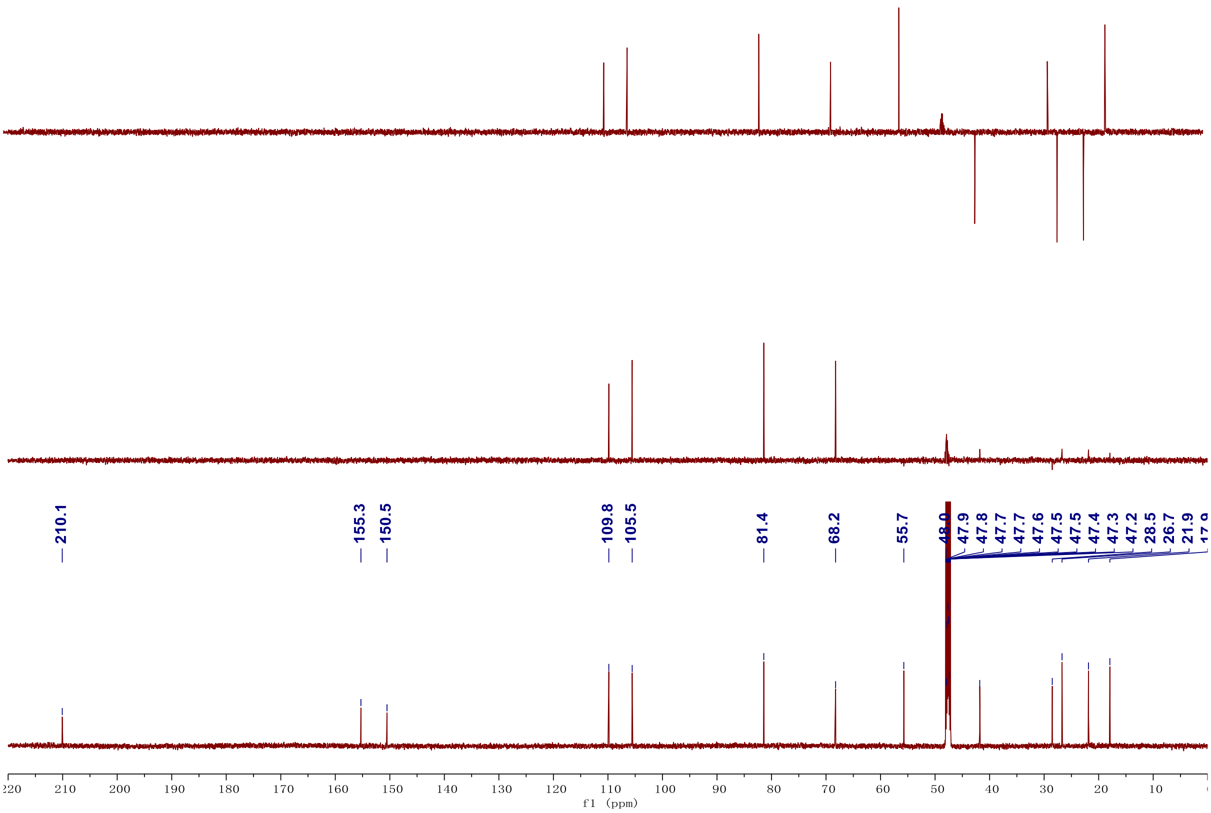

Figure S54. HSQC spectrum of compound **8**

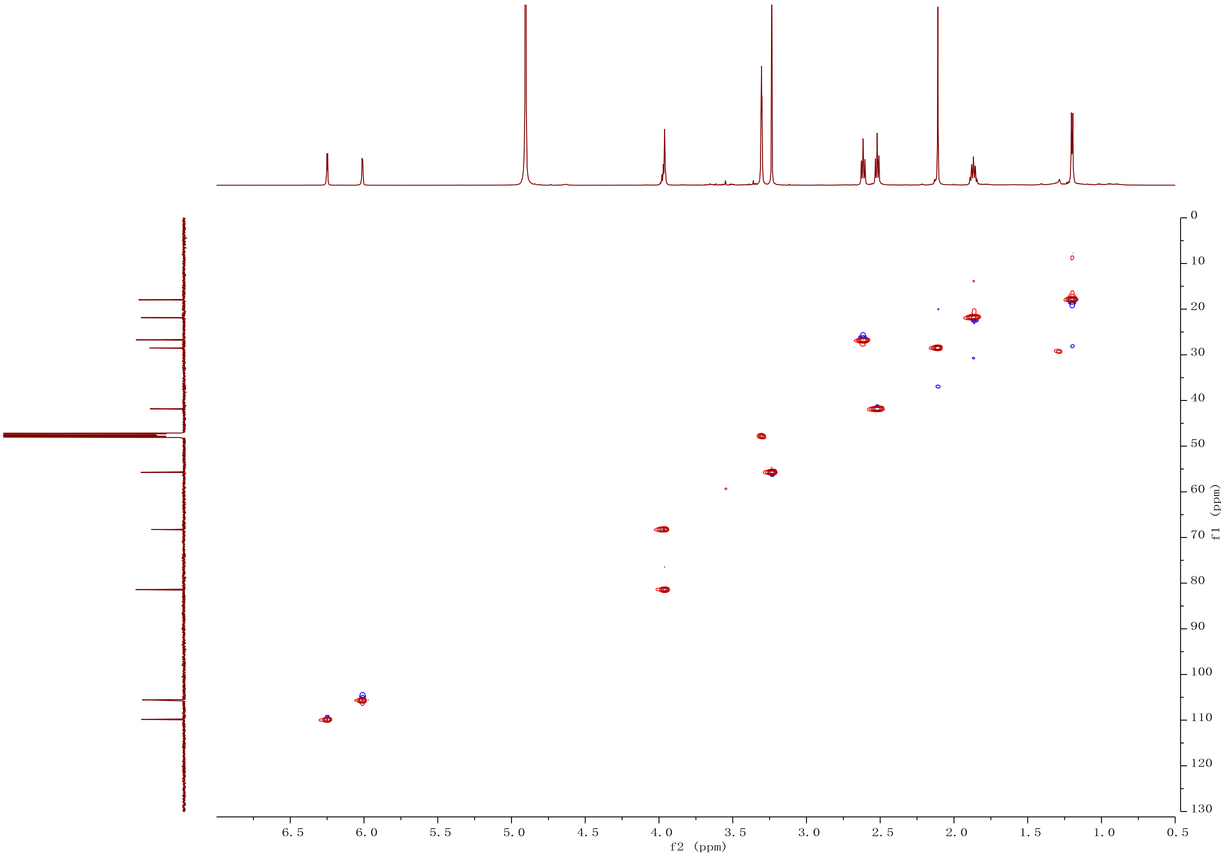

Figure S55. HMBC spectrum of compound **8**

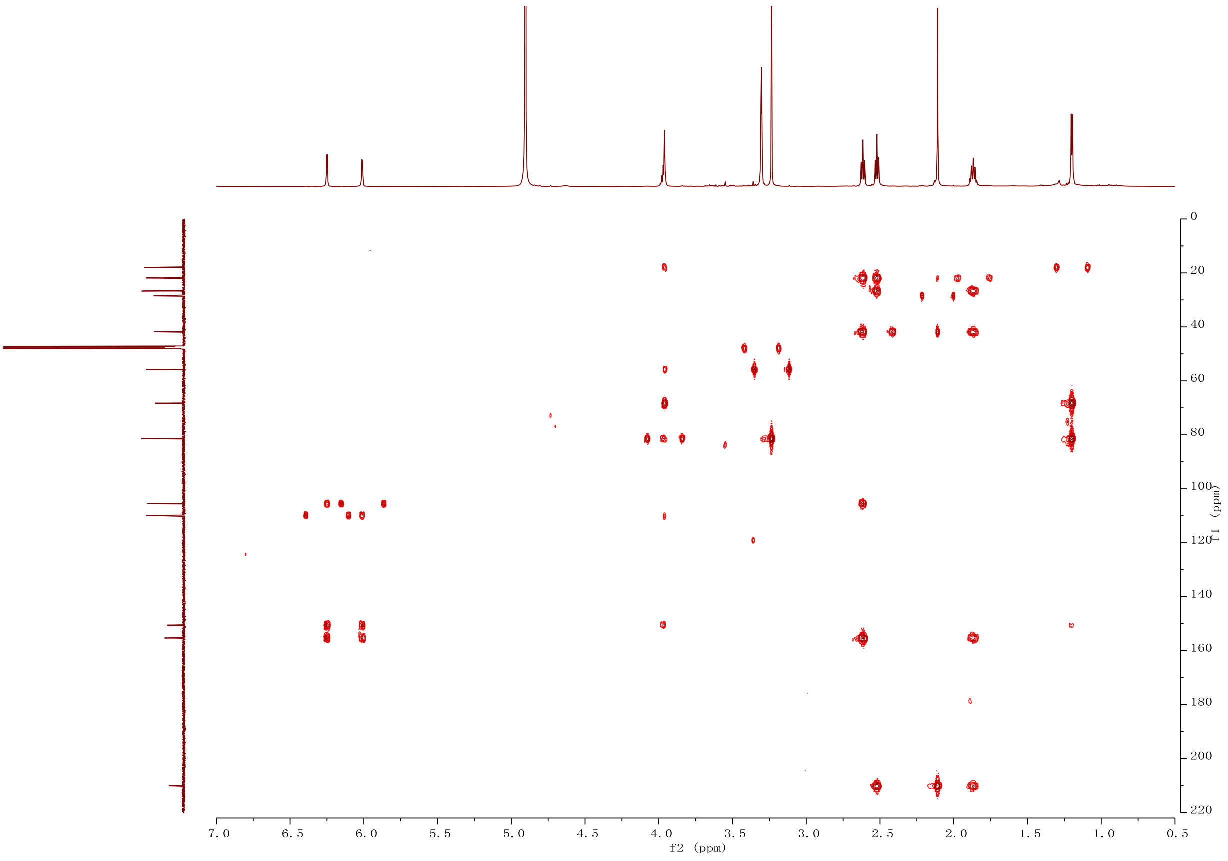

Figure S56. COSY spectrum of compound **8**

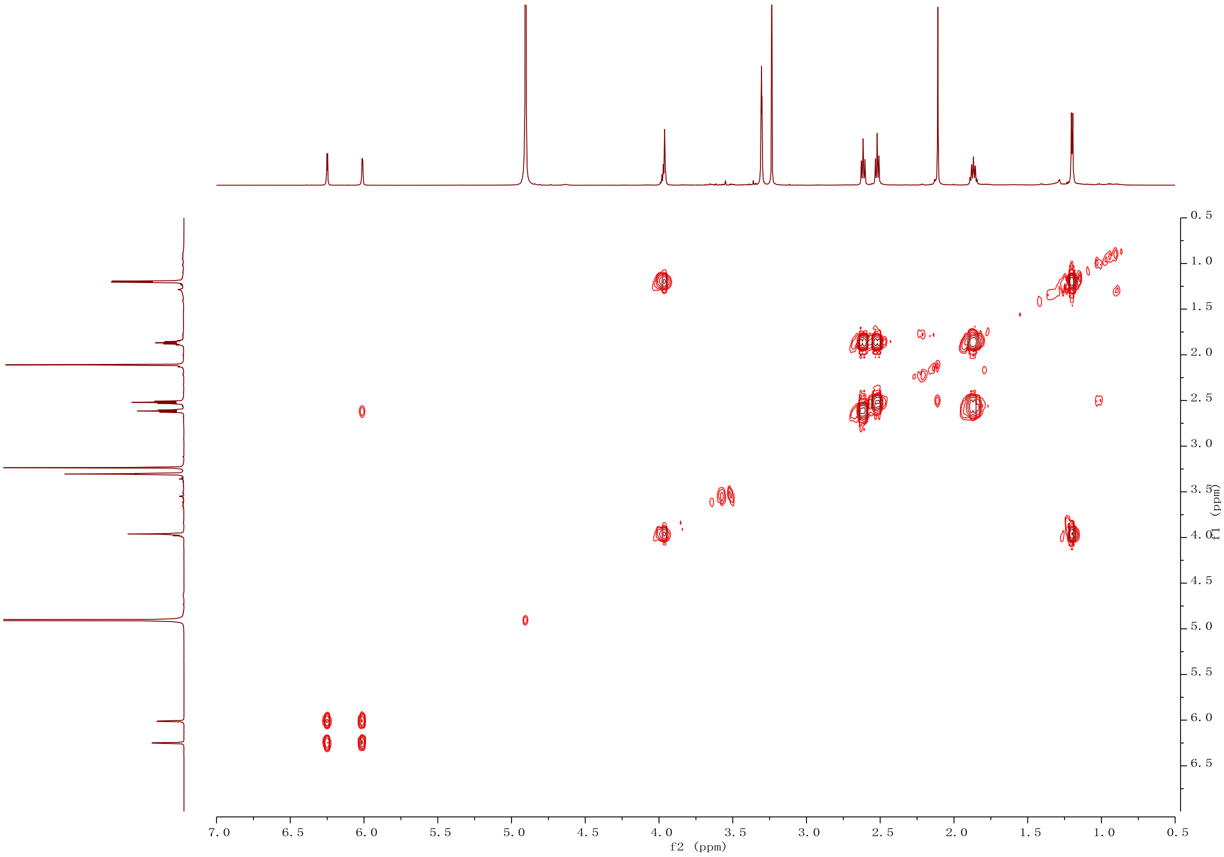

Figure S57. ROESY spectrum of compound **8**

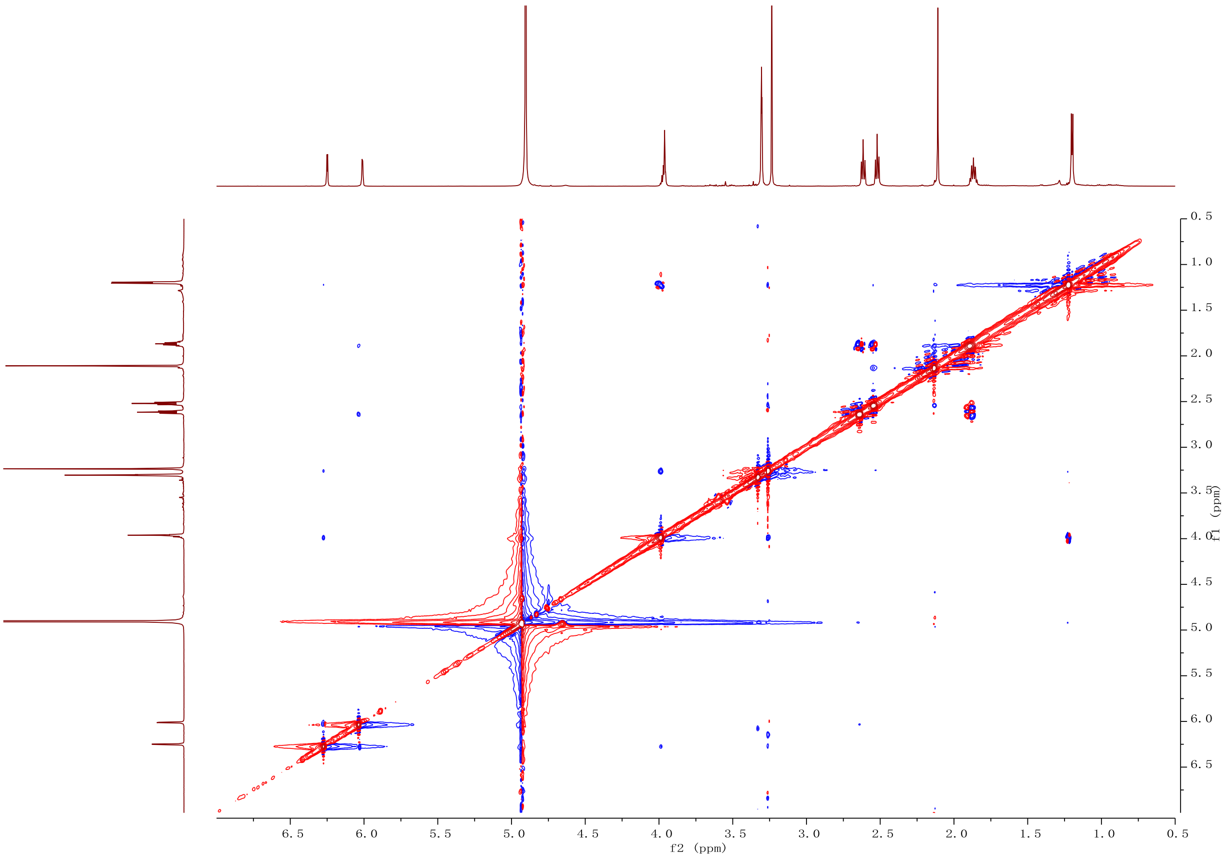

Figure S58. HR-ESI-MS of compound **8**

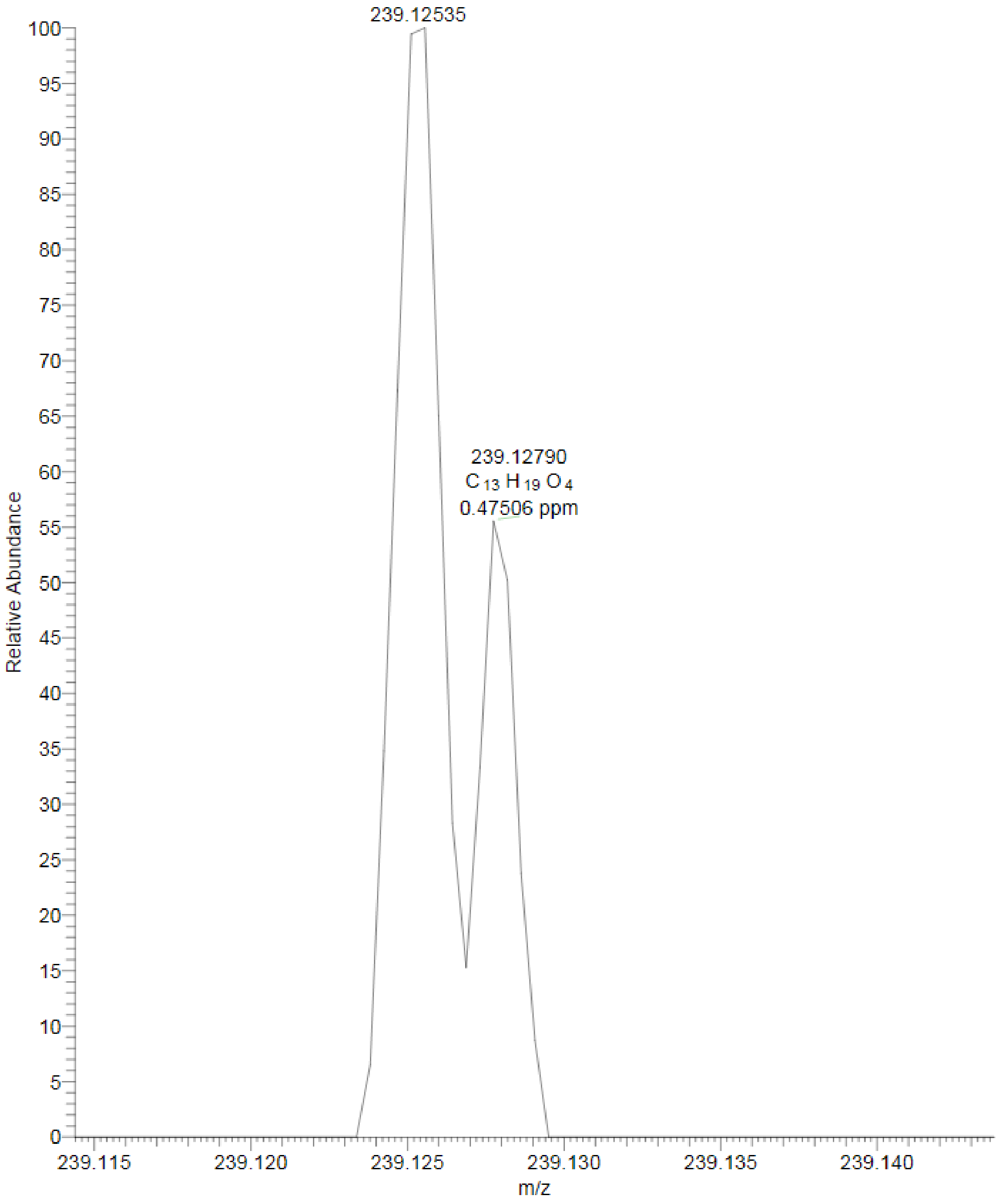

Figure S59. ^{1}H NMR (600 MHz, $CDCl_3$) spectrum of compound **9**

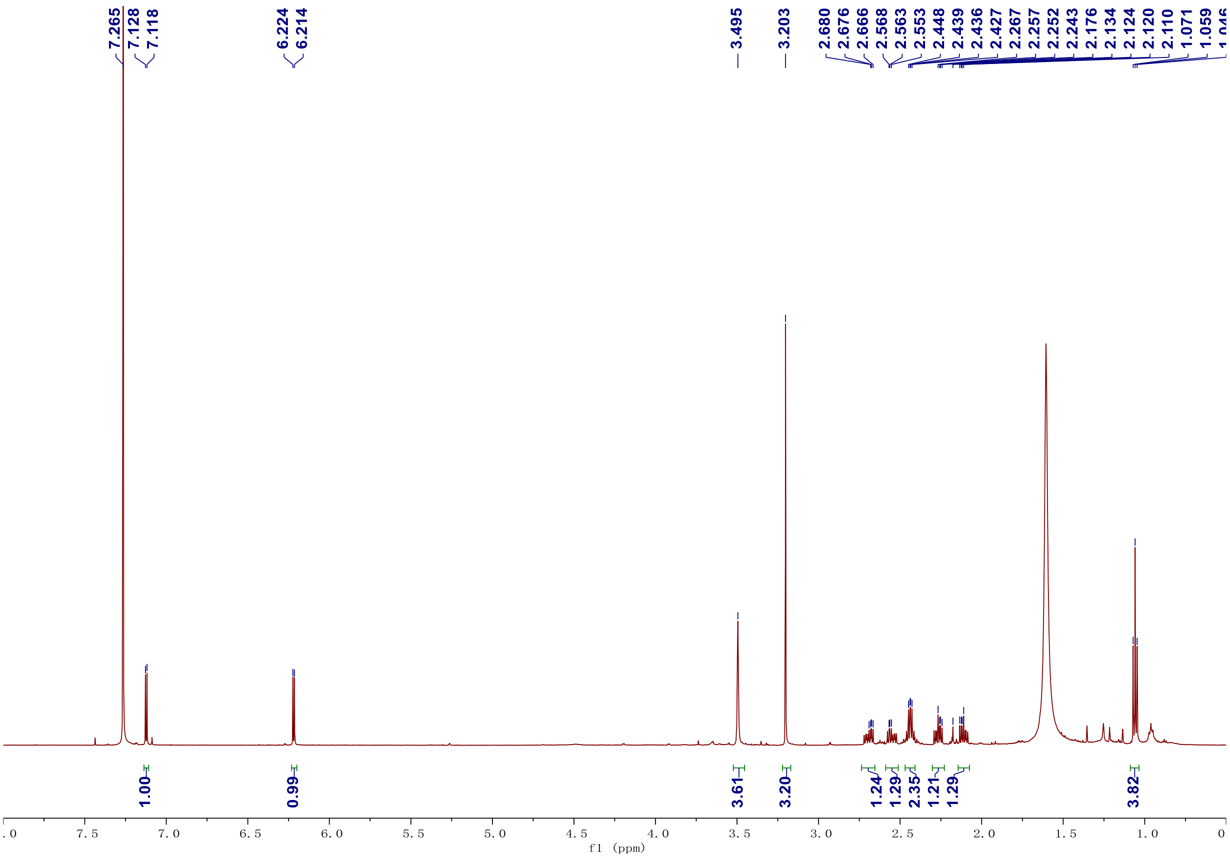

Figure S60. ^{13}C NMR (150 MHz, $CDCl_3$) spectrum of compound **9**

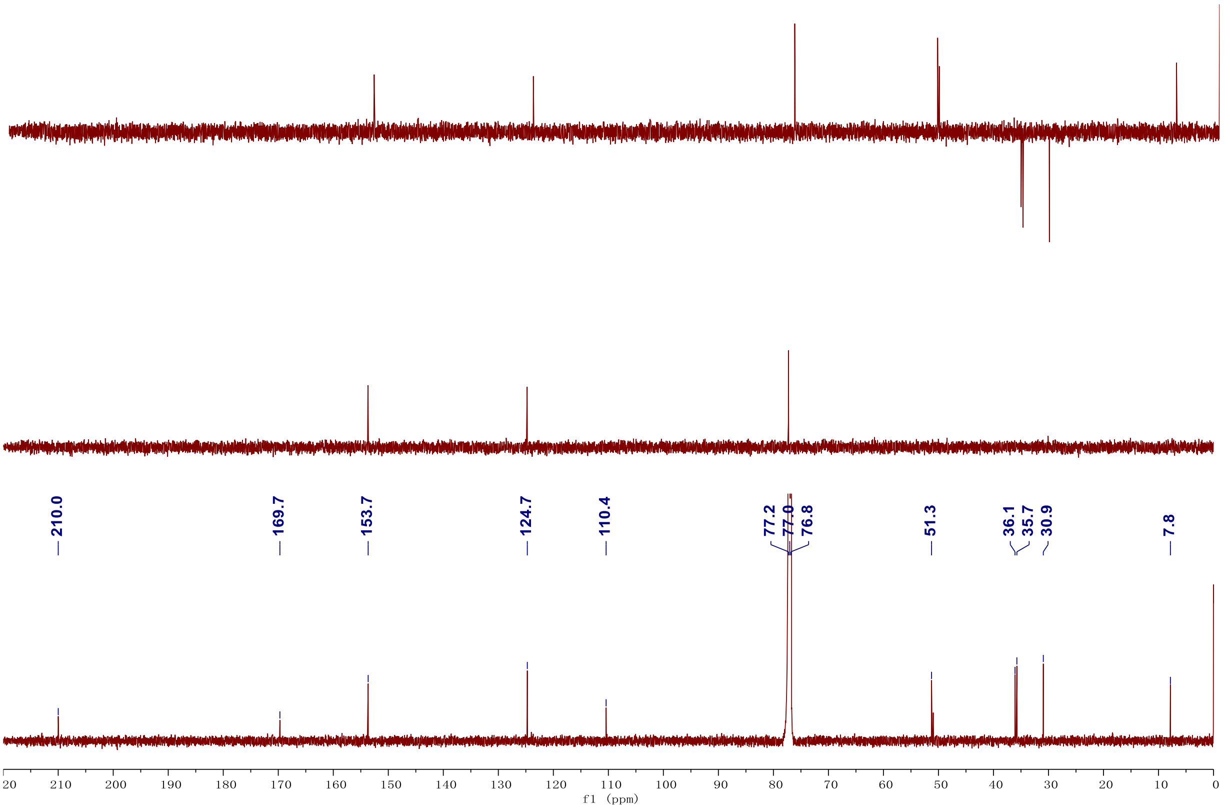

Figure S61. HSQC spectrum of compound **9**

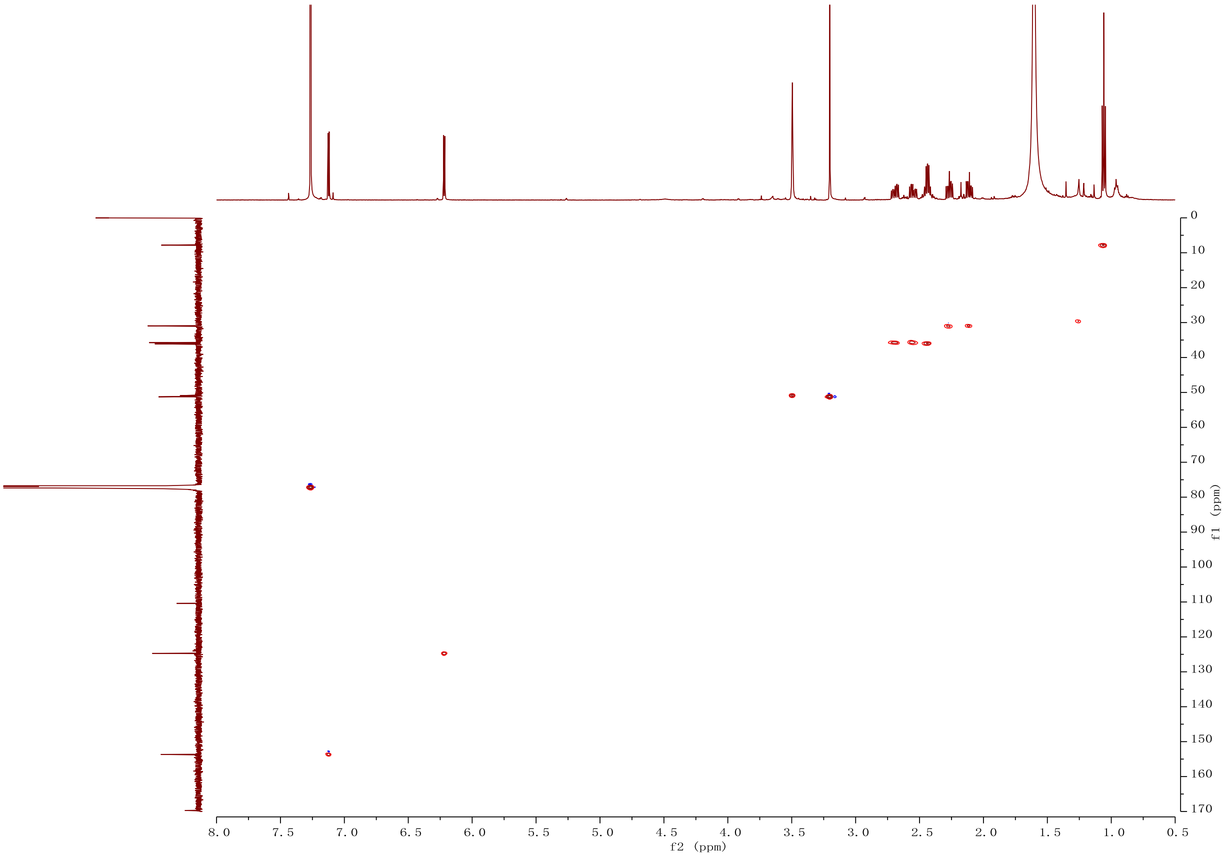

Figure S62. HMBC spectrum of compound **9**

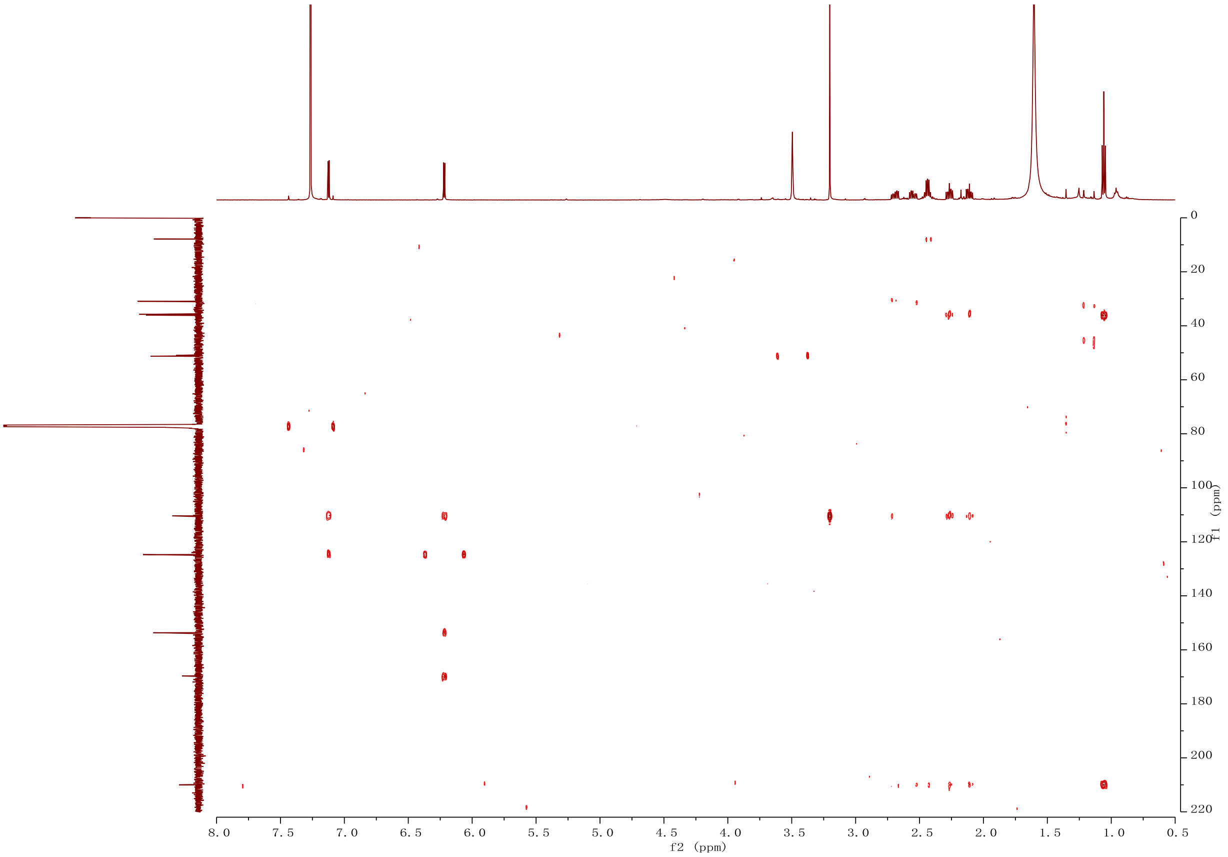

Figure S63. COSY spectrum of compound **9**

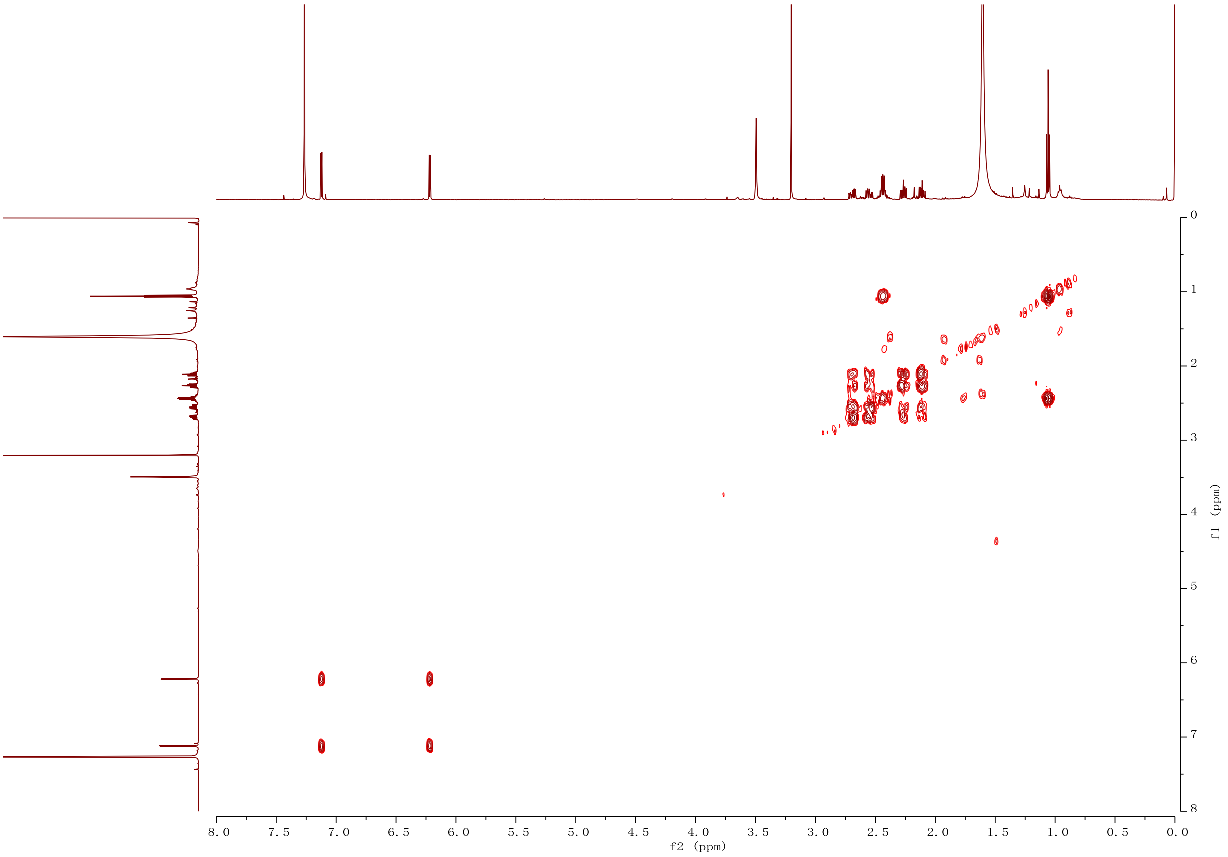

Figure S64. ROESY spectrum of compound **9**

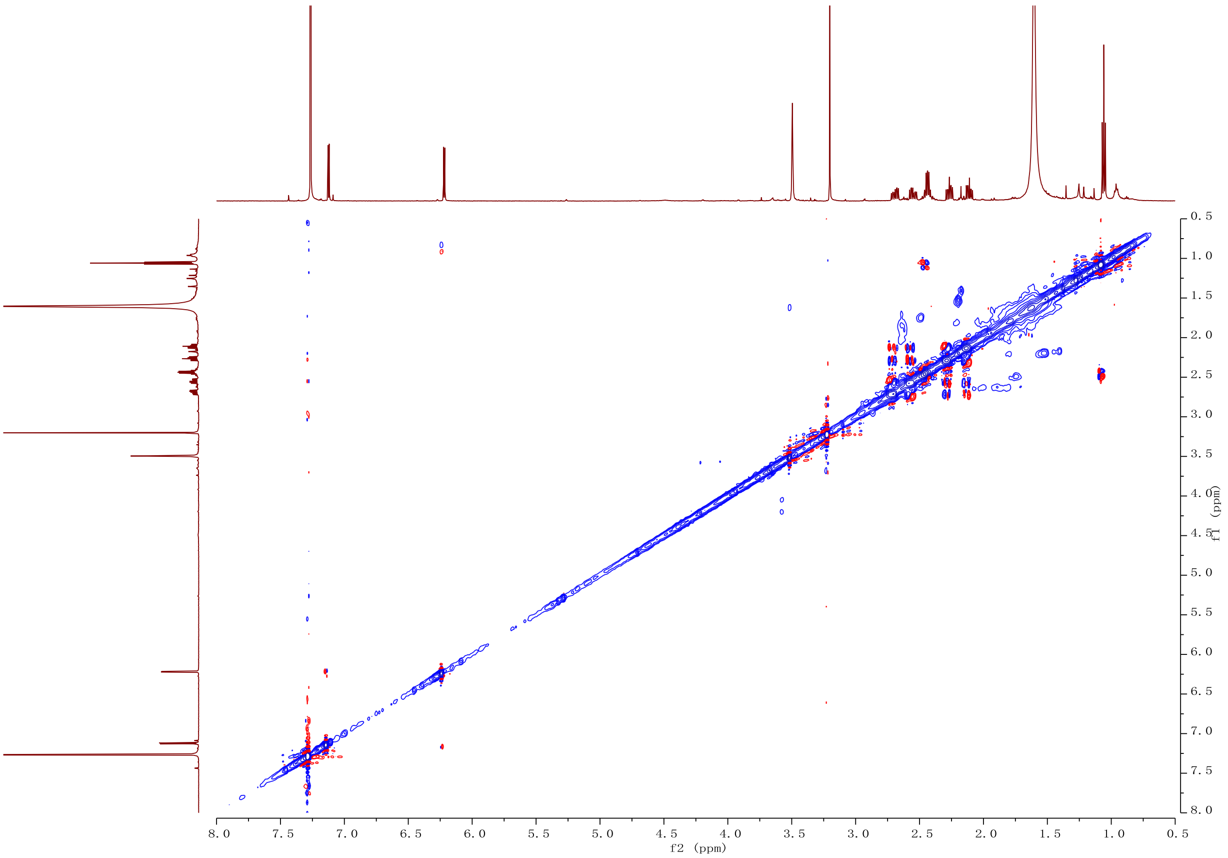

Figure S65. HR-ESI-MS of compound **9**

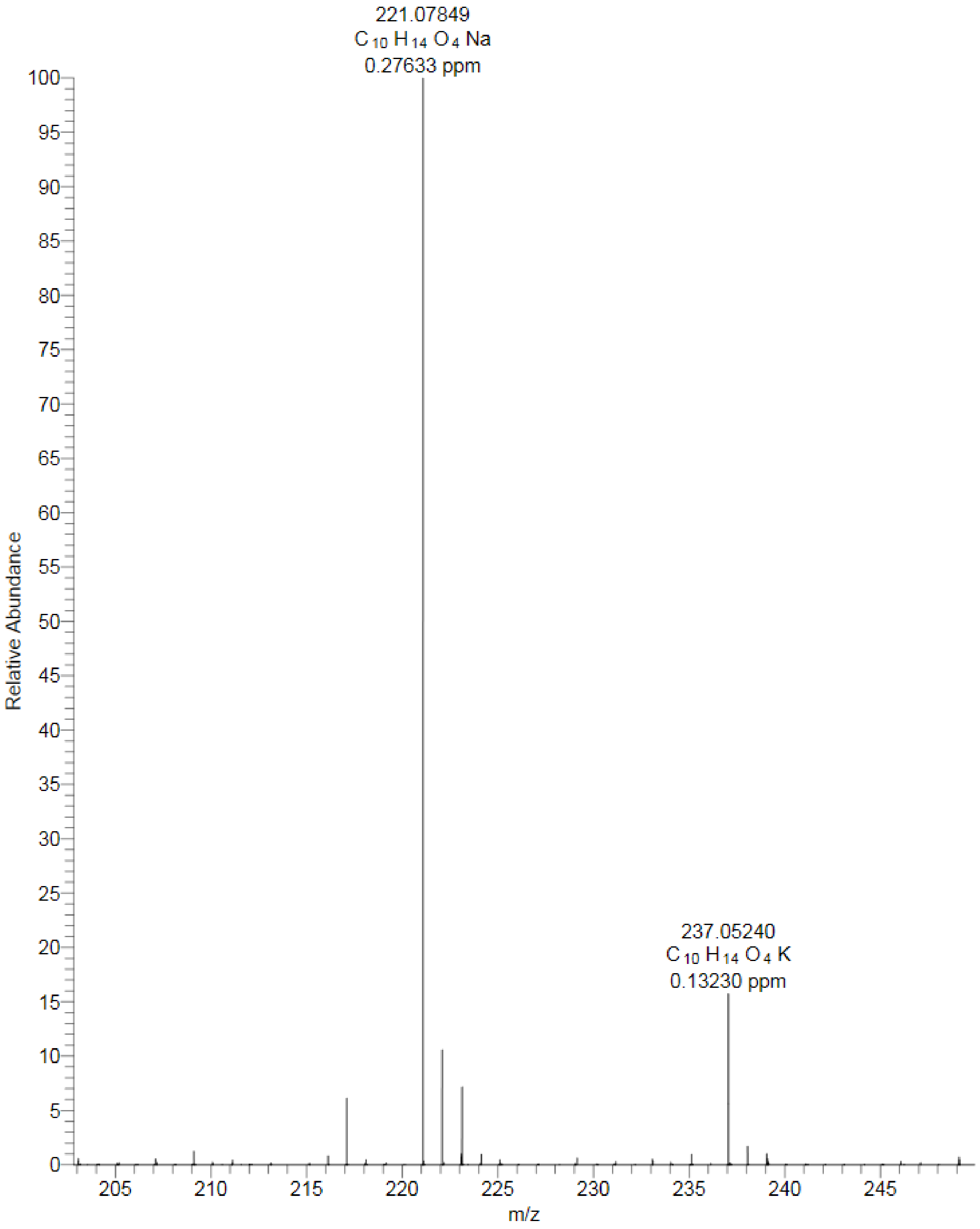

Figure S66. ^{1}H NMR (600 MHz, $CDCl_3$) spectrum of compound **10**

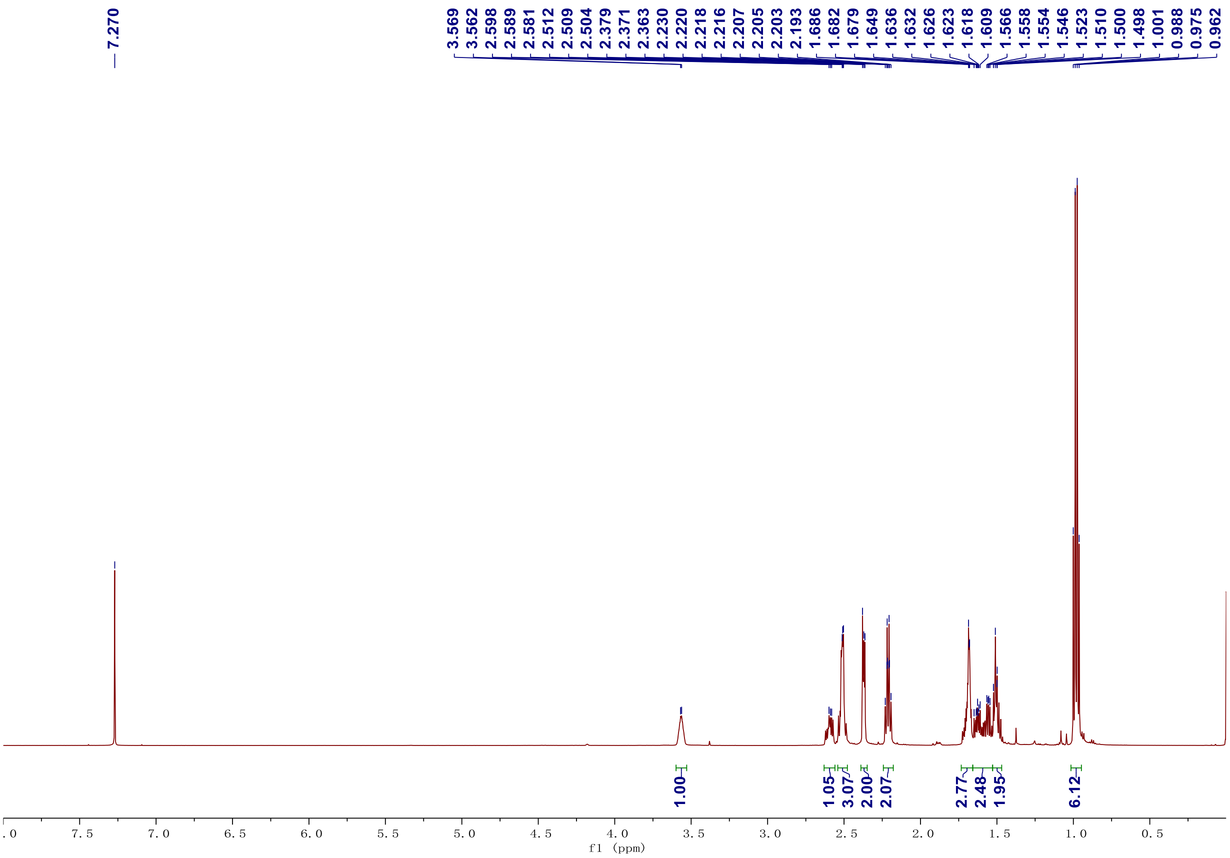

Figure S67. ^{13}C NMR (150 MHz, $CDCl_3$) spectrum of compound **10**

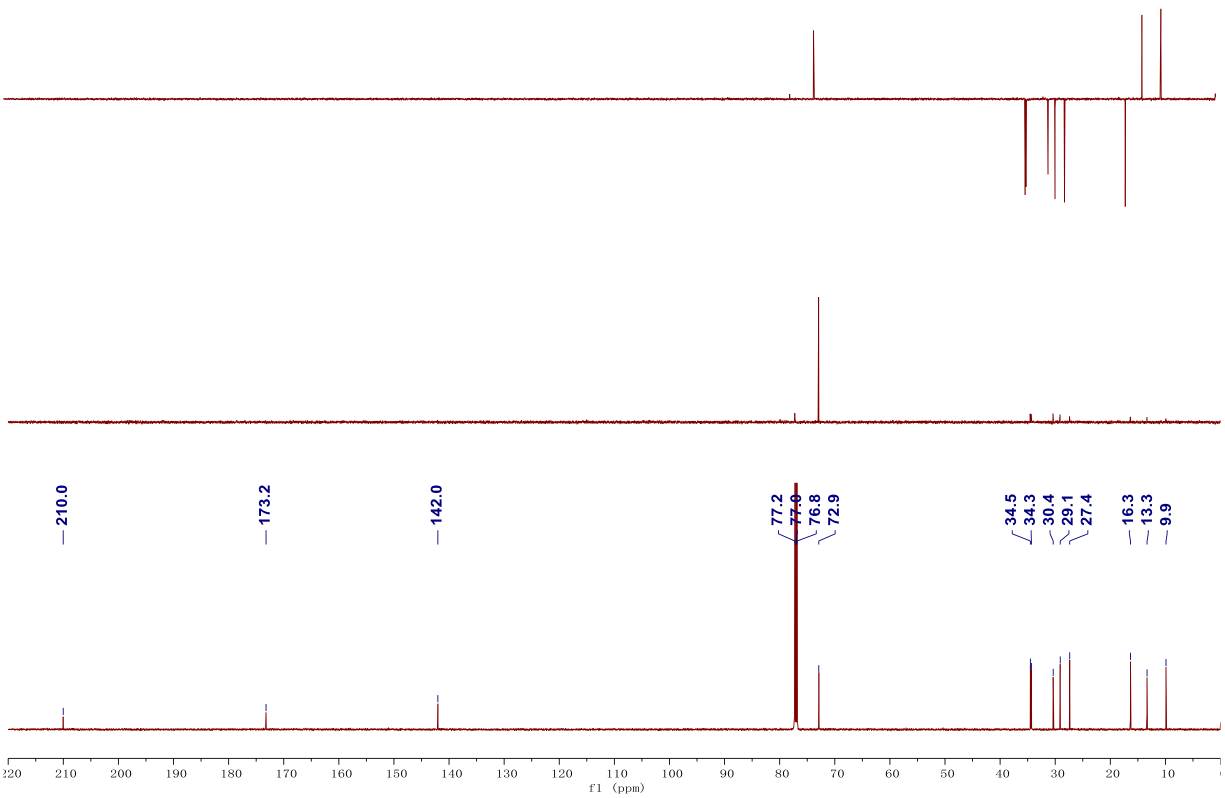

Figure S68. HSQC spectrum of compound **10**

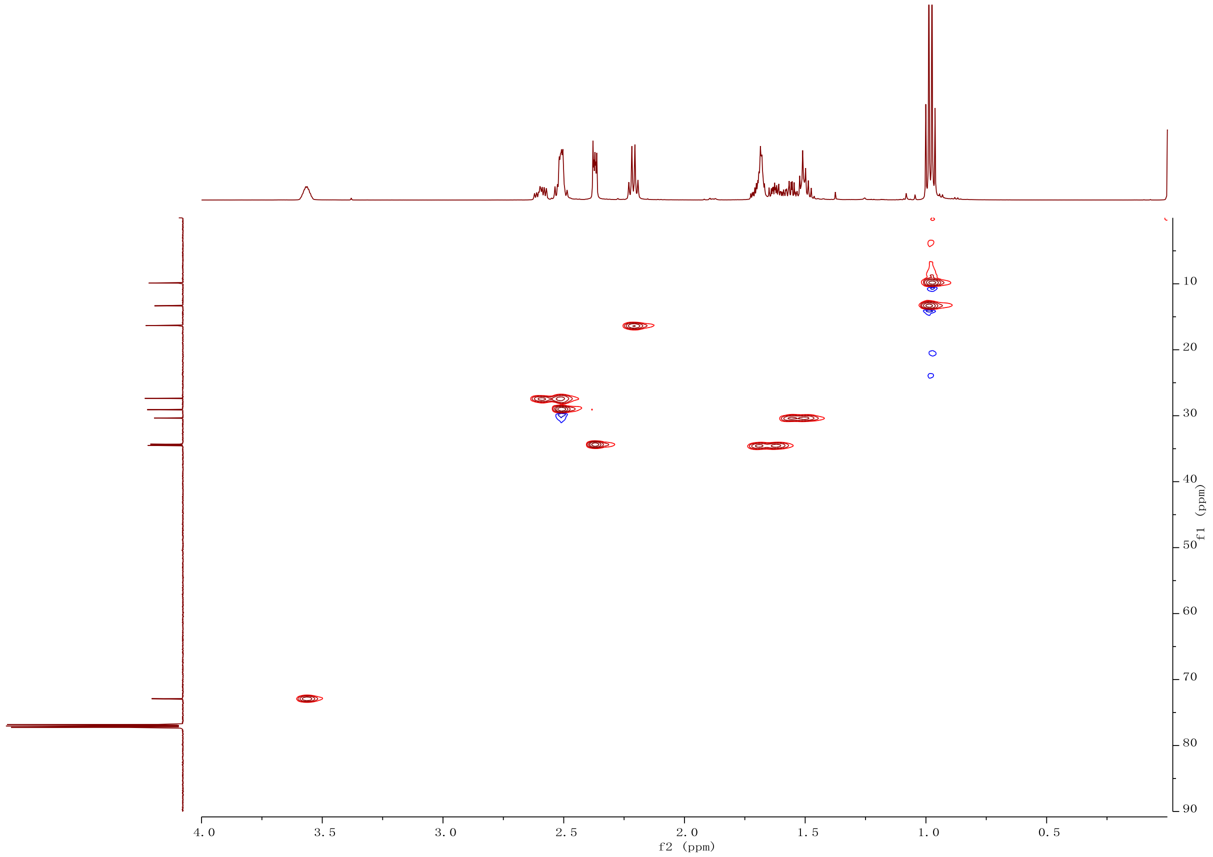

Figure S69. HMBC spectrum of compound **10**

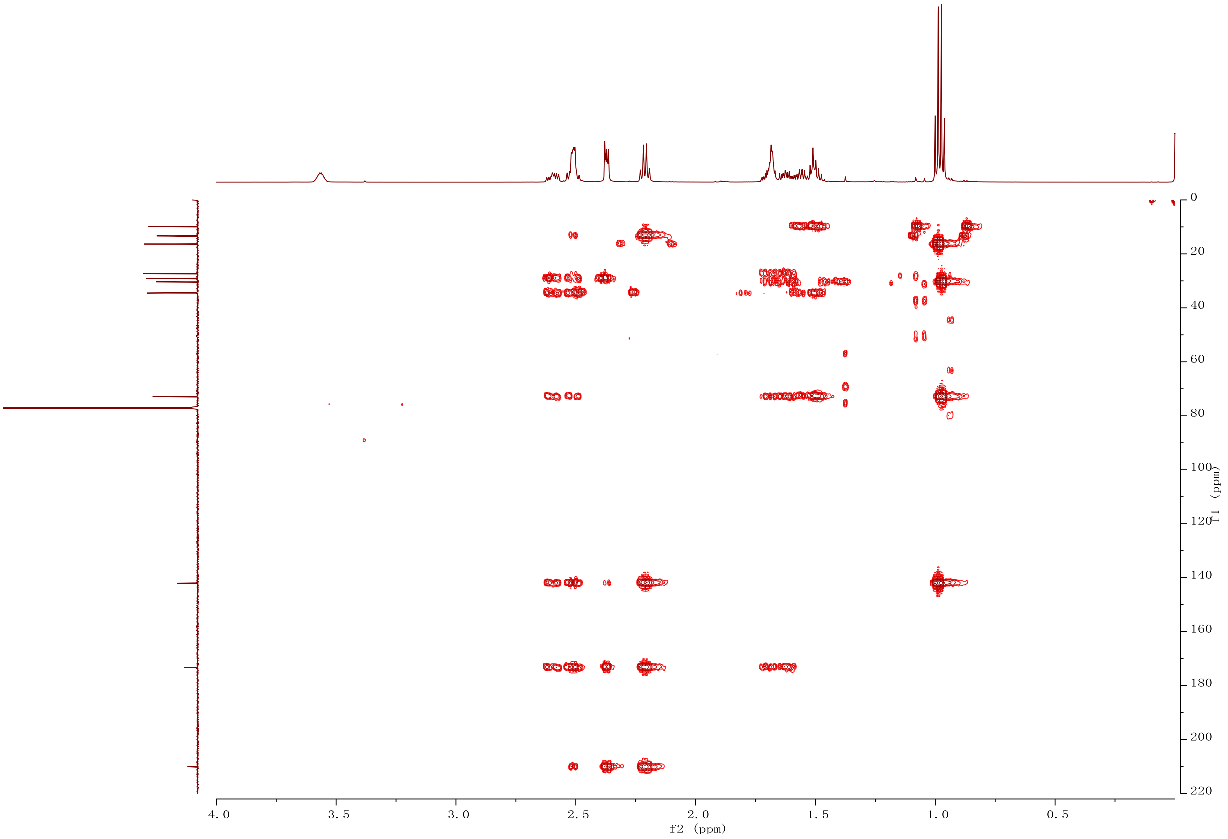

Figure S70. COSY spectrum of compound **10**

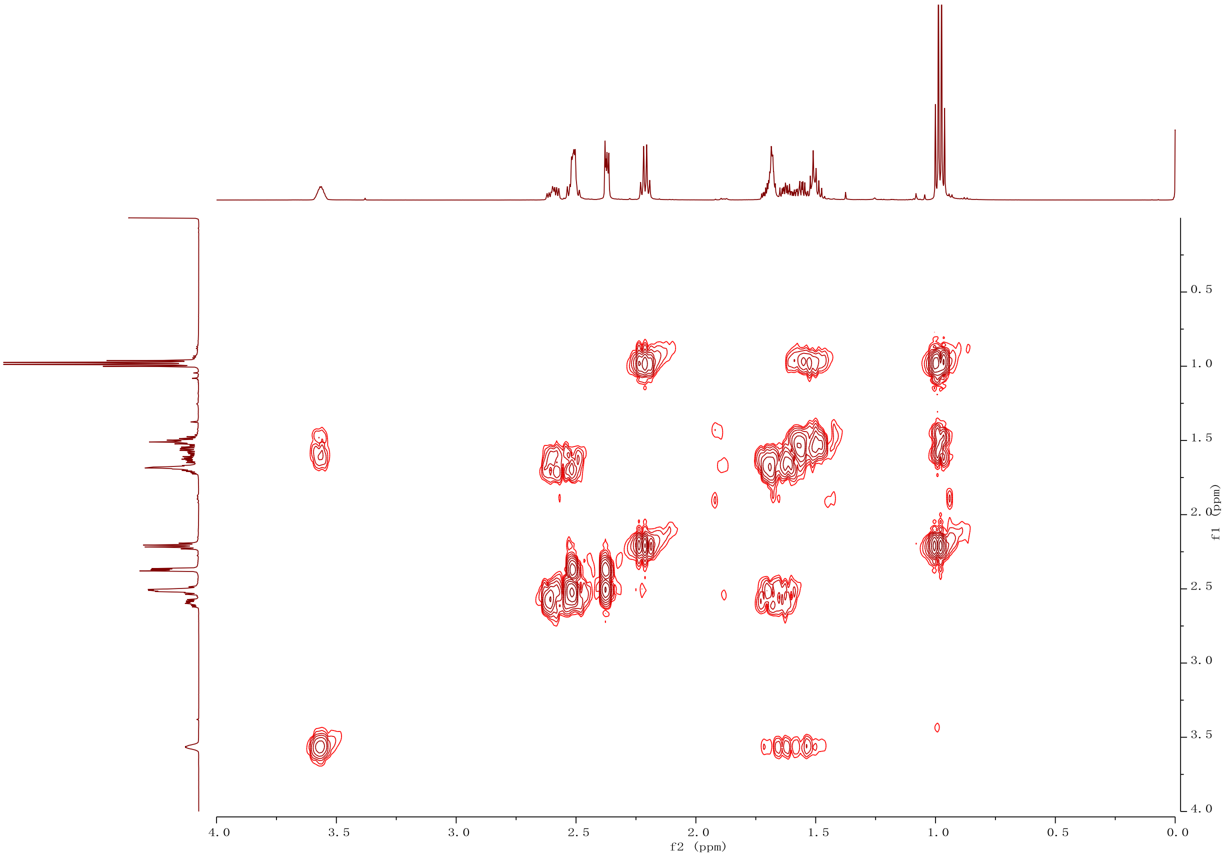

Figure S71. ROESY spectrum of compound **10**

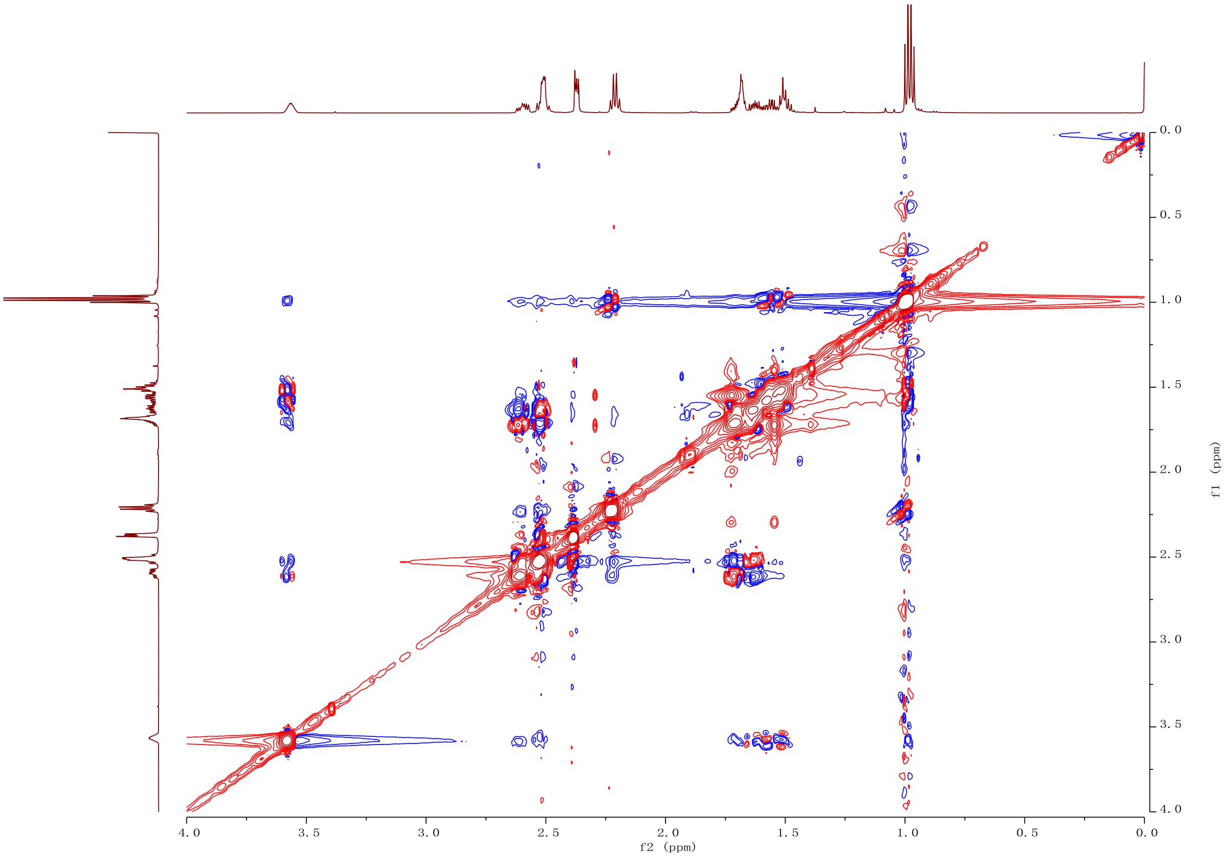

Figure S72. HR-ESI-MS of compound **10**

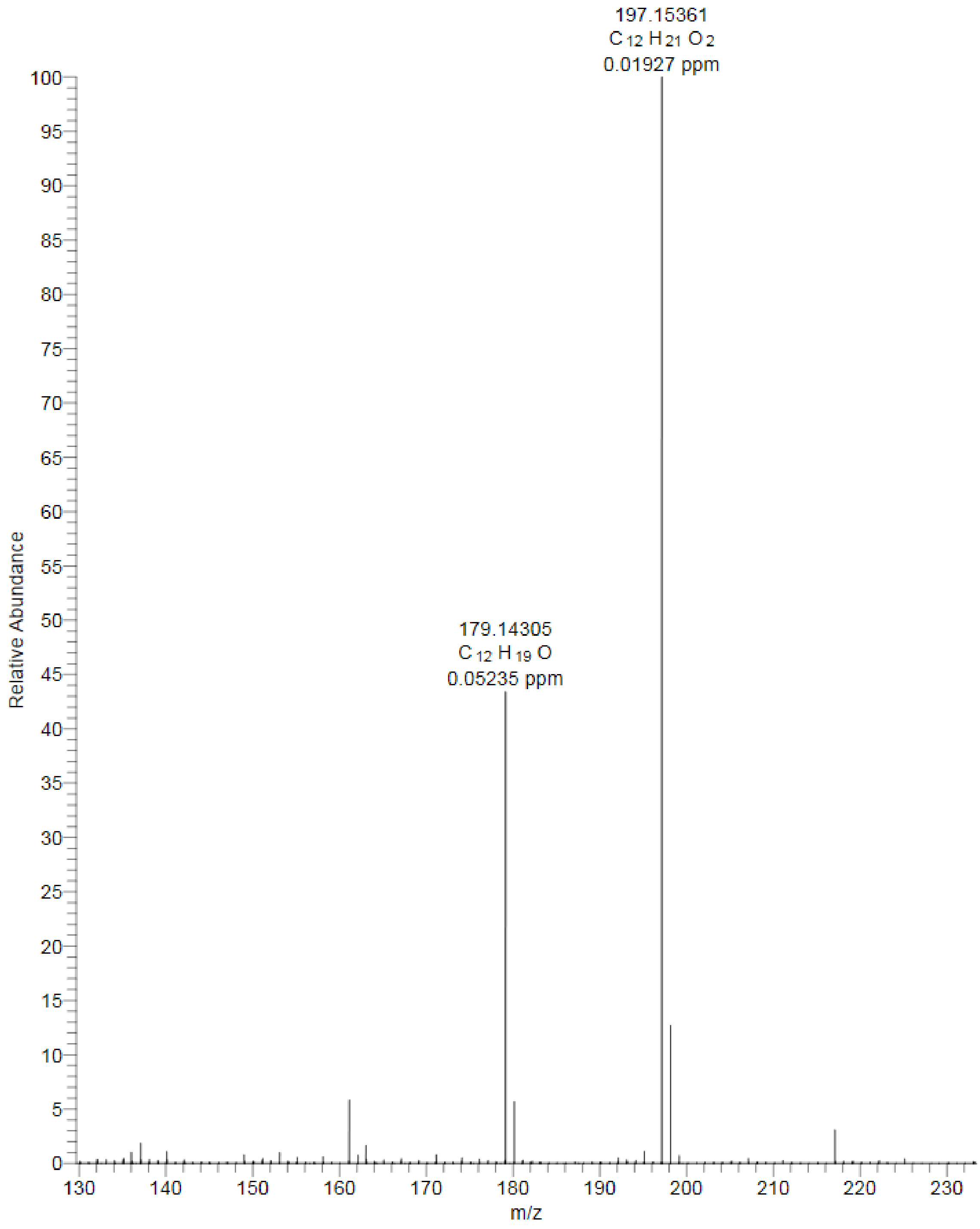

Section S2. Specific Optical Rotation Calculation for compound 6.

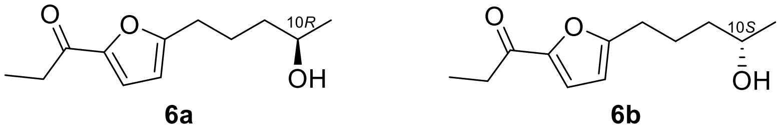

Two plausible configurations of 10*R* (**6a**) and 10*S* (**6b**) for compound **6**.

Systematic conformational analyses were performed via Spartan 16 using the MMFF94 molecular mechanics force field calculation.[1,2] All of the conformers were further optimized by the density functional theory (DFT) method at the B3LYP/TZVP level in Gaussian 09 program package.[3] These conformers were subjected to specific optical rotation calculations at the B3LYP/6-311++G(2d,p) level in methanol with PCM model. The calculated specific optical rotation data of these conformers were averaged according to the Boltzmann distribution theory and their relative Gibbs free energy.[4]

Table S1. *Energy analysis for conformers of* ***6aA-6aP*** *at B3LYP/TZVP level in the gas phase*

Species	*E'=E+ZPE*	*E*	*H*	*G*	*ΔG*	*ΔE*(kcal/mol)66	*PE*%	$[\alpha]_D$
**6aA**	-693.799030	-693.782619	-693.781675	-693.845162	0.000888	0.557228	5.72%	10.33
**6aB**	-693.798989	-693.782596	-693.781652	-693.845011	0.001039	0.651982	4.87%	9.07
**6aC**	-693.798356	-693.781777	-693.780833	-693.845353	0.000697	0.437374	7.00%	-21.89
**6aD**	-693.799206	-693.782633	-693.781689	-693.846050	0.000000	0.000000	14.65%	69.76
**6aE**	-693.797989	-693.781424	-693.780480	-693.845385	0.000665	0.417294	7.24%	22.79
**6aF**	-693.798982	-693.782630	-693.781686	-693.844973	0.001077	0.675828	4.68%	-24.71
**6aG**	-693.798040	-693.781463	-693.780519	-693.845033	0.001017	0.638177	4.99%	-14.46
**6aH**	-693.797876	-693.781284	-693.780340	-693.845040	0.001010	0.633785	5.02%	14.90
**6aI**	-693.798837	-693.782313	-693.781369	-693.845881	0.000169	0.106049	12.25%	-60.54
**6aJ**	-693.798729	-693.782198	-693.781254	-693.845468	0.000582	0.365211	7.91%	37.14
**6aK**	-693.797800	-693.781323	-693.780379	-693.844384	0.001666	1.045431	2.51%	-10.17
**6aL**	-693.798802	-693.782232	-693.781287	-693.845741	0.000309	0.193900	10.56%	-52.02
**6aM**	-693.797892	-693.781642	-693.780698	-693.843731	0.002319	1.455195	1.25%	-4.42
**6aN**	-693.798233	-693.781656	-693.780712	-693.845115	0.000935	0.586721	5.44%	26.30
**6aO**	-693.796970	-693.780376	-693.779432	-693.843745	0.002305	1.446409	1.27%	-7.17
**6aP**	-693.797505	-693.781081	-693.780137	-693.843926	0.002124	1.332830	1.54%	-5.59
**Total**								**-10.70**

Table S2. *Energy analysis for conformers of **6bA-6bR** at B3LYP/TZVP level in the gas phase*

Species	*E'*=*E*+*ZPE*	*E*	*H*	*G*	*ΔG*	*ΔE*(kcal/mol)	*PE*%	$[\alpha]_D$
**6bA**	-693.799030	-693.782619	-693.781675	-693.845162	0.000889	0.557856	5.06%	-9.22
**6bB**	-693.798988	-693.782595	-693.781651	-693.845009	0.001042	0.653865	4.31%	-8.07
**6bC**	-693.798356	-693.781777	-693.780833	-693.845354	0.000697	0.437374	6.21%	19.57
**6bD**	-693.799206	-693.782633	-693.781689	-693.846051	0.000000	0.000000	12.99%	-62.37
**6bE**	-693.797989	-693.781424	-693.780480	-693.845386	0.000665	0.417294	6.42%	-20.38
**6bF**	-693.798974	-693.782624	-693.781680	-693.844962	0.001089	0.683358	4.10%	21.63
**6bG**	-693.798982	-693.782630	-693.781686	-693.844974	0.001077	0.675828	4.15%	22.10
**6bH**	-693.798040	-693.781463	-693.780519	-693.845034	0.001017	0.638177	4.42%	12.93
**6bI**	-693.797876	-693.781284	-693.780340	-693.845041	0.001010	0.633785	4.46%	-13.33
**6bJ**	-693.798837	-693.782313	-693.781369	-693.845881	0.000170	0.106677	10.85%	54.07
**6bK**	-693.798729	-693.782198	-693.781254	-693.845467	0.000584	0.366466	7.00%	-33.13
**6bL**	-693.797800	-693.781323	-693.780379	-693.844384	0.001667	1.046058	2.22%	9.09
**6bM**	-693.798802	-693.782232	-693.781287	-693.845741	0.000310	0.194528	9.36%	46.46
**6bN**	-693.797713	-693.781160	-693.780215	-693.844157	0.001894	1.188503	1.75%	-3.23
**6bO**	-693.799198	-693.782875	-693.781931	-693.844834	0.001217	0.763679	3.58%	-16.87
**6bP**	-693.797885	-693.781236	-693.780292	-693.844842	0.001209	0.758659	3.61%	-7.86
**6bQ**	-693.797824	-693.781270	-693.780326	-693.844971	0.001080	0.677710	4.14%	18.09
**6bR**	-693.796785	-693.780208	-693.779264	-693.844017	0.002034	1.276354	1.51%	-5.27
**Total**								**24.21**

1. Shao, Y.; Molnar, L. F.; Jung, Y.; Kussmann, J.; Ochsenfeld, C.; Brown, S. T.; Gilbert, A. T. B.; Slipchenko, L. V.; Levchenko, S. V.; O'Neill, D. P.; DiStasio Jr, R. A.; Lochan, R. C.; Wang, T.; Beran, G. J. O.; Besley, N. A.; Herbert, J. M.; Yeh Lin, C.; Van Voorhis, T.; Hung Chien, S.; Sodt, A.; Steele, R. P.; Rassolov, V. A.; Maslen, P. E.; Korambath, P. P.; Adamson, R. D.; Austin, B.; Baker, J.; Byrd, E. F. C.; Dachsel, H.; Doerksen, R. J.; Dreuw, A.; Dunietz, B. D.; Dutoi, A. D.; Furlani, T. R.; Gwaltney, S. R.; Heyden, A.; Hirata, S.; Hsu, C.-P.; Kedziora, G.; Khalliulin, R. Z.; Klunzinger, P.; Lee, A. M.; Lee, M. S.; Liang, W.; Lotan, I.; Nair, N.; Peters, B.; Proynov, E. I.; Pieniazek, P. A.; Min Rhee, Y.; Ritchie, J.; Rosta, E.; David Sherrill, C.; Simmonett, A. C.; Subotnik, J. E.; Lee Woodcock Iii, H.; Zhang, W.; Bell, A. T.; Chakraborty, A. K.; Chipman, D. M.; Keil, F. J.; Warshel, A.; Hehre, W. J.; Schaefer Iii, H. F.; Kong, J.; Krylov, A. I.; Gill, P. M. W.; Head-Gordon, M., Advances in methods and algorithms in a modern quantum chemistry program package. *Phys. Chem. Chem. Phys.* **2006,** 8, (27), 3172-3191.
2. Hehre, W. J., *A guide to molecular mechanics and quantum chemical calculations*. Wavefunction, Inc.: Irvine, CA, 2003; p 1-812.
3. Frisch, M. J. T., G. W.; Schlegel, H. B.; Scuseria, G. E.; Robb, M. A.; Cheeseman, J. R.; Scalmani, G.; Barone, V.; Mennucci, B.; Petersson, G. A.; Nakatsuji, H.; Caricato, M.; Li, X.; Hratchian, H. P.; Izmaylov, A. F.; Bloino, J.; Zheng, G.; Sonnenberg, J. L.; Hada, M.; Ehara, M.; Toyota, K.; Fukuda, R.; Hasegawa, J.; Ishida, M.; Nakajima, T.; Honda, Y.; Kitao, O.; Nakai, H.; Vreven, T.; Montgomery, J. A.; Jr., Peralta, J. E.; Ogliaro, F.; Bearpark, M.; Heyd, J. J.; Brothers, E.; Kudin, K. N.; Staroverov, V. N.; Keith, T.; Kobayashi, R.; Normand, J.; Raghavachari, K.; Rendell, A.; Burant, J. C.; Iyengar, S. S.; Tomasi, J.; Cossi, M.; Rega, N.; Millam, J. M.; Klene, M.; Knox, J. E.; Cross, J. B.; Bakken, V.; Adamo, C.; Jaramillo, J.; Gomperts, R.; Stratmann, R. E.; Yazyev, O.; Austin, A. J.; Cammi, R.; Pomelli, C.; Ochterski, J. W.; Martin, R. L.; Morokuma, K.; Zakrzewski, V. G.; Voth, G. A.; Salvador, P.; Dannenberg, J. J.; Dapprich, S.; Daniels, A. D.; Farkas, O.; Foresman, J. B.; Ortiz, J. V.; Cioslowski, J.; and Fox, D. J. *Gaussian 09, revision D. 01*, Gaussian, Inc.: Wallingford CT., 2010.
4. Stephens, P. J.; Devlin, F. J.; Cheeseman, J. R.; Frisch, M. J., Calculation of optical rotation using density functional theory. *J. Phys. Chem. A* **2001,** 105, (22), 5356-5371.
